# Supplementary material for: Structured follow-up pathway to support people after transient ischaemic attack and minor stroke (SUPPORT TIA): protocol for a feasibility study and process evaluation
Source: BMJ Open. 2022 Jun 16;12(6):e060280. doi: 10.1136/bmjopen-2021-060280 (PMC9207897; doi:10.1136/bmjopen-2021-060280)
Supplement: Supplementary data [file bmjopen-2021-060280supp001.pdf]

Summary of intervention development, underpinned by the theoretical framework - Behaviour Change Wheel

- **Select target behaviour:** Healthcare provider identifying and addressing needs post-TIA/ minor stroke.
- **Identify what needs to change:**
  - Semi-structured interviews were conducted with healthcare providers and patients to identify influences on the target behaviour (results reported elsewhere). Transcripts were coded using the Theoretical Domains Framework (TDF).
- **Map TDF domains to "intervention functions"**
  - Four relevant intervention function identified: Education, Training, Environmental restructuring, Enablement.
- **Identify Behaviour Change Techniques (BCT):**
  - The BCT Taxonomy was used to identity appropriate BCT that mapped to relevant intervention functions.

The above process was informed by relevant literature; iterative feedback from patient partners and a multidisciplinary team (nurses, allied health professionals, GPs, consultants, researcher); and consideration of the APEASE criteria (Affordability, Practicality, Effectiveness and cost-effectiveness, Acceptability, Side-effects/safety, and Equity).

eTable 1: Summary of the barriers/ enablers mapped to Theoretical Domain Framework domains, intervention functions, Behaviour Change Taxonomies and intervention components.

Barriers (B)/ Enablers (E)	TDF	BCW intervention function	BCT	SUPPORT TIA Intervention component
HCPs' lack of knowledge of potential needs post-TIA/ minor stroke (B).<br><br>HCPs' perceived role in follow-up care influenced their approach to identifying and addressing needs (B/E).	Knowledge<br><br>Social professional role and identity<br><br>Goal	Education/ Training	*Information about health consequences*<br><br>*Information about social and environmental consequences*<br><br>*Information about emotional consequences*<br><br>*Instruction on how to perform the behaviour*<br><br>*Identity associated with changed behaviour*	Training for intervention providers

Lack of follow-up pathway following rapid hospital specialist review (B).<br><br>Time constraints (B).<br><br>Nurses and AHPs were more holistic in their approach to care (compared to doctors) and considered this part of their role (E).	Environmental context and resources<br><br>Social professional role and identity<br><br>Intentions	Environmental restructuring	*Restructure physical environment*	Structured nurse/AHP-led follow-up appointment
Checklists considered useful prompts to identify needs (E).		Environmental restructuring/ Enablement	*Prompt/ cues*	Needs checklist completed by participants prior to the appointment
HCPs' lack of knowledge of support services and resources to support needs (B).<br><br>Directories of support services facilitated identification of support services (if up-to-date) (E).	Knowledge<br><br>Environmental context and resources	Environmental restructuring/ Enablement	*Instruction on how to perform the behaviour*<br><br>*Adding objects to the environment*	Resources to support management of needs, including a website of resources and support services; list of local support services; and a self-management booklet
Patients feel unsupported after hospital (B).<br><br>Difficult for patients to process and retain information (B).<br><br>Patients attempted to access information and support themselves, but found it overwhelming, confusing, contradictory (B).	Social influences	Environmental restructuring/ Enablement	*Action planning*<br><br>*Goal setting*<br><br>*Problem solving*<br><br>*Pharmacological support*	Action plan
Restricted communication between primary and secondary care (B).<br><br>Variability in the speed and content of GP letters (B/E)	Environmental context and resources<br><br>Social influences	Environmental restructuring/ Enablement	*Action planning*<br><br>*Prompts/cues*	Structured GP letter
