# Supplementary material for: Structured follow-up pathway to support people after transient ischaemic attack and minor stroke (SUPPORT TIA): protocol for a feasibility study and process evaluation
Source: BMJ Open. 2022 Jun 16;12(6):e060280. doi: 10.1136/bmjopen-2021-060280 (PMC9207897; doi:10.1136/bmjopen-2021-060280)
Supplement: Supplementary data [file bmjopen-2021-060280supp002.pdf]

TNO | | | | |

My **information** and **support needs** after mini-stroke

This is a list of needs that some people have after mini stroke.

Please tick (✓) any areas you have difficulty with, even if you have only experienced it occasionally.

Your follow-up appointment is an opportunity to discuss any worries or problems you might be experiencing, and any advice or information you may need following your mini stroke (TIA) or minor stroke. Please use this list to help you to prepare for your appointment.

Information

1. I need more information about:	*Tick here*
• My diagnosis	
• My risk of stroke	
• Driving after my mini stroke	

Stroke prevention

2. I need advice on:	*Tick here*
• Medications for preventing another stroke	
• Medication side effects	
• Lifestyle change, such as exercise or diet, to prevent another stroke	

Effects of mini-stroke

3. Fatigue	*Tick here*
• I feel tired most of the time or I get easily tired	
• I find it difficult to concentrate and do things	
**4. Mood**	
• I feel anxious	
• I feel depressed	
• I experience anger, frustration or mood swings	
• I feel that my personality has changed	
**5. Memory and thinking**	
• I find it difficult to think, concentrate, or remember things	

Turner GM, *et al. BMJ Open* 2022; 12:e060280. doi: 10.1136/bmjopen-2021-060280

TNO | | | | |

**6. Communication**	
• I finding it difficult to understand / communicate with others	
• I have problems with speech, word finding or talking to others	
**7. Physical**	
• I experience muscle weakness or problems with balance	
• I have headaches	
• I am sensitive to noise or light	
**8. Incontinence**	
• I am having a problem controlling my bladder or bowels	
**9. Intimate relationships**	
• Since my mini stroke I have problems with sex	
**10. Work or education**	
• I am having problems at work or education	
• I would like support and advice on returning to work or education	
**11. Relationships with family or friends**	
• My personal relationships with my family or friends have become difficult or stressed	
**12. Social activities or daily tasks**	
• I find it difficult to take part in hobbies or leisure activities	
• I have difficulty doing daily tasks	

**Which of the needs you identified above is your biggest concern?**

**Is there anything else you are concerned about or would like more information about?**

Please bring this list to your follow-up appointment

Patient's Needs Checklist V1.0 date 19/01/2021 IRAS: 267063 Page **2** of **2**
