# Supplementary material for: Structured follow-up pathway to support people after transient ischaemic attack and minor stroke (SUPPORT TIA): protocol for a feasibility study and process evaluation
Source: BMJ Open. 2022 Jun 16;12(6):e060280. doi: 10.1136/bmjopen-2021-060280 (PMC9207897; doi:10.1136/bmjopen-2021-060280)
Supplement: Supplementary data [file bmjopen-2021-060280supp003.pdf]

eTable 2: Summary of proposed Patient Reported Outcome Measures (PROMs) for the main Randomised Controlled Trial.

Domain	PROM	Summary	Rationale
Health related quality of life	PROMIS-Global Health 10	**Questions:** 10<br>**Scoring:** 5-point Likert + 10 point Likert pain question. Scored using item-level calibrations- use the Health Measures Scoring Service<br>**MID:** none specified<br>**Permissions:** publicly available for use	Recommended by the International Consortium for Health Outcomes Measurement (2016) as part of a consensus stroke measure standard set
Health related quality of life	EQ-5D-5L	**Questions:** 5 + 1 VAS (20 cm)<br>**Scoring:** 5-point Likert scale and VAS. Score = 0-1<br>**MID:** for stroke: 0.08 to 0.12<br>**Permissions:** licence agreement needed (€600)	Used to calculate Quality Adjusted Life Years for health economics analyses
Anxiety/ depression	HADS	**Questions:** 14<br>**Scoring:** 4-point Likert Scale. 0-7 = Normal; 8-10 = Borderline abnormal; 11-21 = Abnormal<br>**MID:** none specified<br>**Permissions:** license agreement needed (0-1000 participants= £0.85 each)	Widely used in TIA/ minor stroke research, comprehensive psychometric evaluation
Fatigue	FAS	**Questions:** 10<br>**Scoring:** 5-point Likert Scale; Score = 10 (lowest fatigue) to 50 (highest fatigue). 10-21: no fatigue (normal), 22-50: substantial fatigue<br>**MID:** at least 4 points or 10% change of the baseline value<br>**Permissions:** must acknowledge the ild care foundation in the manuscript, FAS needs to be added as a keyword in the final publication, and PDF of the final publication must be send to the ild care foundation: info@ildcare.nl	Systematic review of fatigue scales for stroke scales identified the FAS had the best test-retest reliability
Self-efficacy	PAM-13	**Questions**: 13<br>**Scoring:** 5-point Likert Scale; Score 1-100: 4 Levels: Level 1 ≤47.0 (not believing activation is important), Level 2 47.1–55.1 (a lack of knowledge and confidence to take action), Level 3 55.2–67.0 (beginning to take action), level 4 ≥67.1 (taking action)<br>**MID:** not specified<br>**Permissions:** licence agreement NOT needed for feasibility studies	Used by NHS England
Medication adherence	MARS-5	**Questions:** 6<br>**Scoring:** 5-point Likert scale, score= 5 to 25 (higher scores= higher self-reported adherence)<br>**MID:** not specified<br>**Permissions:** Free access (subject to adequate citation) for Academic users	Validated in stroke patients, widely used
Satisfaction with care	Satisfaction with care question	Not applicable- non-validated bespoke question	

EQ-5D-5L: 5-level EuroQol 5-Dimensions; FAS: Fatigue Assessment Scale; HADS: Hospital Anxiety and Depression Scale; MARS-5: Medication adherence: Medication Adherence Rating Scale -5; MID: Minimally Important Difference; NHS: National Health Service; PAM-13: Patient Activation Measure-13; PDF: Portable Document Format; TIA: Transient Ischaemic Attack; VAS: Visual Analogue Scale
