# Supplementary material for: Structured follow-up pathway to support people after transient ischaemic attack and minor stroke (SUPPORT TIA): protocol for a feasibility study and process evaluation
Source: BMJ Open. 2022 Jun 16;12(6):e060280. doi: 10.1136/bmjopen-2021-060280 (PMC9207897; doi:10.1136/bmjopen-2021-060280)
Supplement: Supplementary data [file bmjopen-2021-060280supp004.pdf]

Protocol: additional information

Trial Steering Committee, Data Monitoring Committee and Study Oversight Committee

There will be no Trial Steering Committee or Data Monitoring Committee, the Study Oversight Committee will take the role of a joint Trial Steering Committee and Data Monitoring Committee.

The role of the independent Study Oversight Committee is to provide overall oversight of the trial, including the practical aspects of the study, as well as ensuring that the study is run in a way which is both safe for the patients and provides appropriate feasibility data to the sponsor and investigators.

The Study Oversight Committee will receive only aggregate data and will be blinded to the treatment allocation until the final analysis is presented. Members are independent of the investigators, their employing organisations, funders and sponsors. The Study Oversight Committee will report directly to the Trial Management Group.

Adverse events

The collection and reporting of Adverse Events will be in accordance with the UK Policy Framework for Health and Social Care Research and the requirements of the Health Research Authority. Definitions of different types of Adverse Events are listed in the table of abbreviations and definitions.

No risks are expected to arise from taking part in the trial. There are no Investigational Medicinal Products being used as part of the SUPPORT TIA trial. The intervention is considered low risk and consists of a nurse/AHP-led follow-up appointment which has been used in other populations and settings without evidence of harm. No Serious Adverse Events are anticipated as a unique consequence of participation in the SUPPORT TIA trial, but reporting requirements are clearly outlined in this section.

Adverse Events: There may be certain Adverse Events which are commonly expected in participants who have suffered a TIA or minor stroke. However, as these events are well characterised, it is highly unlikely that this trial will reveal any new safety information relating to this intervention. Therefore, we will not be collecting non-serious Adverse Events for this trial.

Serious Adverse Advents: Investigators should only report Serious Adverse Events which are attributable to the trial intervention. The above events are not considered related to the trial intervention and are therefore excluded from notification to the SUPPORT TIA Trial Office as Serious Adverse Events. These events should continue to be recorded in the medical records according to local practice. We are only reporting Serious Adverse Events which are attributable to the trial intervention; therefore, the control group will not be monitored.

Participant Withdrawal

Participants will be made aware at the beginning that they can freely withdraw (discontinue participation) from the trial (or part of) at any time. Types of withdrawal as defined are:

- The participant would like to withdraw from trial treatment, but is willing to be followed up in accordance with the schedule of assessments and, if applicable, using any central UK NHS bodies for long-term outcomes (i.e. the participant has agreed that data can be collected and used in the trial analysis).

- The participant would like to withdraw from trial treatment and does not wish to attend trial visits in accordance with the schedule of assessments but is willing to be followed up at standard clinic visits and, if applicable, using any central UK NHS bodies for long-term outcomes (i.e. the participant has agreed that data can be collected at standard clinic visits and used in the trial analysis, including data collected as part of long-term outcomes).
- The participant would like to withdraw from trial treatment and is not willing to be followed up in any way for the purposes of the trial and for no further data to be collected (i.e. only data collected prior to the withdrawal can be used in the trial analysis).

The details of withdrawal (date, reason and type of withdrawal) will be clearly documented in the study discontinuation form.

Note: participants involved in the qualitative sub-study may only withdraw from this part of the study up to the point of data analysis (five working days following the interview). After this point, it will not be possible to extract an individuals' interview data from the analyses.

Monitoring

Monitoring will be conducted as required following a risk assessment by the trials unit. Given the low-risk nature of this trial, central monitoring will be routine and no onsite monitoring is planned.

Onsite Monitoring: For this trial, no onsite monitoring is planned due to the low risk of the intervention and nature of the outcome data.

Central Monitoring: Trials staff will be in regular contact with the site research team to check on progress and address any queries that they may have. Recruitment rates, per site, will be monitored on a monthly basis. Trials staff will check incoming Informed Consent Forms and Case Report Forms for compliance with the protocol, data consistency, missing data and timing. Sites will be sent Data Clarification Forms requesting missing data or clarification of inconsistencies or discrepancies. Sites will be requested to send in copies of signed Informed Consent Forms and other documentation for in-house review for all participants providing explicit consent. Structured observations will be conducted as part of the trial of recruitment and consent procedures; intervention appointments; and end of study clinic appointments. Reports from these observations will be used to monitor protocol compliance.

Audit and Inspection: The Investigator will permit trial-related monitoring, audits, ethical review, and regulatory inspection(s) at their site, providing direct access to source data/documents. The investigator will comply with these visits and any required follow up. Sites are also requested to notify Birmingham Clinical Trials Unit (BCTU) of any relevant inspections.

Protocol amendments

If the Chief Investigator wishes to make a substantial amendment to the Research Ethics Committee (REC) application or the supporting documents, the Chief Investigator will submit a valid notice of amendment to the REC for consideration. It is the Sponsor's responsibility to decide whether an amendment is substantial or non-substantial for the purposes of submission to the REC.

Amendments will be notified to the REC and Health Research Authority (HRA), and communicated to the participating organisations (R&D office and local research team) departments of participating sites to assess whether the amendment affects the NHS permission for that site. The amendment history will be tracked to identify the most recent protocol version.

Turner GM, *et al. BMJ Open* 2022; 12:e060280. doi: 10.1136/bmjopen-2021-060280

Confidentiality

Personal data recorded on all documents will be regarded as strictly confidential and will be handled and stored in accordance with the Data Protection Act 2018.

Participants will be identified using their unique trial identification number in correspondence between the site and BCTU. Participants will give their explicit consent for the movement of their consent form, giving permission for BCTU to be sent a copy. This will be used to perform in-house monitoring of the consent process. Participants will provide their personal contact details to the central research team at BCTU so they are able to contact the participants for follow-up questionnaires.

The Investigator must maintain documents not for submission to BCTU in strict confidence. In the case of specific issues and/or queries from the regulatory authorities, it will be necessary to have access to the complete trial records, provided that participant confidentiality is protected.

BCTU will maintain the confidentiality of all participant's data and will not disclose information by which participants may be identified to any third party, with the exception of the transcription service. A professional transcription company that already works with the University of Birmingham will transcribe the audio files. This company will be required to sign a confidentiality agreement before any files are sent to them. A member of the research team will check the transcripts once received from the transcription company and remove any names/ identifiers from the documents. Once the accuracy of the transcriptions has been confirmed, the original recordings will be deleted.

Representatives of the SUPPORT TIA trial team and sponsor may be required to have access to participant's notes for quality assurance purposes but participants should be reassured that their confidentiality will be respected at all times.

For participants involved in the qualitative aspects of the study, we will ask their permission to audio record the study interview using a digital recording device. We will then ask a reputable company to produce a written version of the recording called a transcript. The transcript company will need to sign a confidentiality agreement before they do so. We will then anonymise the transcript, removing all identifying information. After this, we will delete the original recording. We will only use anonymised quotes from the transcript in any arising publications or reports.

The research team will hold personal contact data for participants wishing to receive a summary of the results of the study - we anticipate this will be made available within 12 months of completion of the study. We will delete participants' contact details when the data is archived, meaning no personal identifiable data, other than study consent forms, will be retained.

There is potential that participants may disclose information that either indicates a risk or harm to themselves or others, evidence of malpractice or criminality. Participants will be informed in the Participant Information Sheet that if they disclose any of these issues, this will be reported to appropriate authorities.

Dissemination: Authorship eligibility guidelines and any intended use of professional writers

Publication policy: Results of this trial will be submitted for publication in a peer reviewed journal. The manuscript will be prepared by the Chief Investigator and authorship will be determined by the trial publication policy. No professional writers will be used.

Informed consent materials: Model consent form

I have read and understood the participant information sheet (Version 3.0, Dated 06/09/2021).	
I have had the opportunity to take time to consider my involvement in the trial and I have had the chance to ask questions, all of which have been answered to my satisfaction.	
I understand that my involvement in the trial is voluntary, and I am free to withdraw at any time without the quality of my medical care or my legal rights being affected.	
I understand that if I decide to withdraw from the trial, any information that has already been collected and anonymised may be used for analysis and publication.	
I understand a copy of this consent form and my data collected during the trial will be transferred to the central trials office at the Birmingham Clinical Trials Unit (BCTU), part of the University of Birmingham.	
I understand that all information collected will be used for medical research only and that I will not be identified in any way in the analysis and reporting of results. The personal information will include name, gender, date of birth, contact details and NHS number as well as medical information and study outcome assessments. It will be held securely and confidentially at BCTU.<br>I give permission for the transfer and storage of this data.	
I understand that relevant sections of my medical notes, information related directly to my participation in this trial, and data collected during the trial may be looked at by individuals from the University BCTU trial team, representatives of the sponsor, regulatory authorities, and the NHS Trust/Health Board where it is relevant to my taking part in this research.<br>I give permission for these individuals to have access to my records.	
I understand that my data collected during the trial may be shared with academic collaborative third parties who will help with the study's analysis. Before sharing, my personal identifiers (e.g. name, date of birth, contact details) will be removed and replaced with a trial number.	
I understand that my name and contact details will be used by the study team to contact me regarding the study. This may include, but not limited to, request additional information such as missing data on questionnaires I have completed.	
I understand that the research team have a duty to inform appropriate authorities if I disclose information that either indicates a risk or harm to myself or others, evidence of malpractice or criminality. In this circumstance confidentiality may be broken.	
I give permission to my GP being informed about my participation in the SUPPORT TIA Trial	
I voluntarily agree to take part in this study.	
Optional: I agree to be contacted to be invited for an interview about my involvement in the study and agree to my contact details being passed on to the research team at the University of Birmingham for them to contact me about this interview	
Optional: I would like to be sent a summary of the findings from the study and consent to my contact details to be held until this summary has been sent.	

Name of Participant	Signature	Date (DD/MMM/YYYY)
Name of Person taking Consent	Signature	Date (DD/MMM/YYYY)

4
