# Supplementary material for: Structured follow-up pathway to support people after transient ischaemic attack and minor stroke (SUPPORT TIA): protocol for a feasibility study and process evaluation
Source: BMJ Open. 2022 Jun 16;12(6):e060280. doi: 10.1136/bmjopen-2021-060280 (PMC9207897; doi:10.1136/bmjopen-2021-060280)
Supplement: Author's manuscript [file bmjopen-2021-060280.draft_revisions.pdf]

BMJ Open is committed to open peer review. As part of this commitment we make the peer review history of every article we publish publicly available.

When an article is published we post the peer reviewers' comments and the authors' responses online. We also post the versions of the paper that were used during peer review. These are the versions that the peer review comments apply to.

The versions of the paper that follow are the versions that were submitted during the peer review process. They are not the versions of record or the final published versions. They should not be cited or distributed as the published version of this manuscript.

BMJ Open is an open access journal and the full, final, typeset and author-corrected version of record of the manuscript is available on our site with no access controls, subscription charges or pay-per-view fees (http://bmjopen.bmj.com).

If you have any questions on BMJ Open's open peer review process please email info.bmjopen@bmj.com

BMJ Open

Structured follow-up pathway to support people after transient ischaemic attack and minor stroke (SUPPORT TIA): Protocol for a feasibility study and process evaluation

Journal:	*BMJ Open*
Manuscript ID	bmjopen-2021-060280
Article Type:	Protocol
Date Submitted by the Author:	16-Dec-2021
Complete List of Authors:	Turner, Grace; Institute of Applied Health Research University of Birmingham; University of Birmingham<br>Jones, Rachael; Royal Wolverhampton NHS Trust<br>Collis, Phillip; Institute of Applied Health Research University of Birmingham; University of Birmingham<br>Patel, Smitaa; University of Birmingham, Birmingham Clinical Trials Unit<br>Jowett, Sue; University of Birmingham, Health Economics Unit<br>Tearne, Sarah ; University of Birmingham Clinical Trials Unit<br>Foy, Robbie; University of Leeds, Leeds Institute of Health Sciences<br>Atkins, Lou; UCL<br>Mant, Jonathan; University of Cambridge, General Practice and Primary Care Research Unit<br>Calvert , Melanie ; University of Birmingham, Centre for Patient Reported Outcomes Research and Institute of Applied Health Research
Keywords:	STROKE MEDICINE, REHABILITATION MEDICINE, Organisation of health services < HEALTH SERVICES ADMINISTRATION & MANAGEMENT, Protocols & guidelines < HEALTH SERVICES ADMINISTRATION & MANAGEMENT, Depression & mood disorders < PSYCHIATRY, QUALITATIVE RESEARCH

SCHOLARONE™
Manuscripts

Structured follow-up pathway to support people after transient ischaemic attack and minor stroke (SUPPORT TIA): Protocol for a feasibility study and process evaluation

Grace M Turner* 1, 2

Rachael Jones 3, 4

Phillip Collis 1, 2

Smitaa Patel 6

Sue Jowett 7

Sarah Tearne 6

Robbie Foy 8

Lou Atkins 9

Jonathan Mant 10

Melanie Calvert 1, 2, 11, 12, 13, 14

1 Institute of Applied Health Research, University of Birmingham, Birmingham, UK

2 Centre for Patient Reported Outcomes Research, University of Birmingham, Birmingham, UK

3 Royal Wolverhampton NHS Trust, Wolverhampton Rd, Heath Town, Wolverhampton, UK

4 Clinical Research Network West Midlands (CRN WM), UK

5 Patient partner, UK

6 Birmingham Clinical Trials Unit, University of Birmingham, Birmingham, UK

7 Health Economics Unit, University of Birmingham, Birmingham, UK

8 Leeds Institute of Health Sciences, University of Leeds, Leeds, UK

9 Centre for Behaviour Change, University College London, London, UK

10 Department of Public Health and Primary Care, University of Cambridge, Cambridge, UK

11 NIHR Surgical Reconstruction and Microbiology Research Centre, University Hospitals Birmingham NHS Foundation Trust and University of Birmingham, Birmingham, UK

12 NIHR Birmingham Biomedical Research Centre, University Hospitals Birmingham NHS Foundation Trust and University of Birmingham, Birmingham, UK

For peer review only - http://bmjopen.bmj.com/site/about/guidelines.xhtml

For peer review only

13 Birmingham Health Partners Centre for Regulatory Science and Innovation, University of Birmingham, Birmingham, UK

14 NIHR Applied Research Collaboration (ARC) West Midlands, University of Birmingham, Birmingham, UK

* Corresponding author: Dr Grace Turner; Institute of Applied Health Research, University of Birmingham, Birmingham, B15 2TT, UK; G.Turner.1@bham.ac.uk

Word count

2,353

Key words

Transient ischaemic attack; mini stroke; TIA; minor stroke; rehabilitation, stroke prevention, follow-up, Hospital Anxiety and Depression Scale, Patient-Reported Outcomes Measurement Information System, 5-level EuroQol 5-Dimensions, Fatigue Assessment Scale, Patient Activation Measure-13, Medication Adherence Rating Scale -5

For peer review only

Abstract

Introduction

Despite rapid specialist review in hospital, people who experience transient ischaemic attack (TIA) and minor stroke have limited follow-up, which means they often have unmet needs and feel abandoned. Care needs after TIA/minor stroke include information provision (diagnosis and stroke risk), stroke prevention (medication and lifestyle change), and holistic care (residual problems and return to work or usual activities). This protocol describes a feasibility study and process evaluation of an intervention to support people after TIA/minor stroke. The study aims to assess the feasibility and acceptability of (i) the intervention and (ii) a randomised controlled trial of this intervention.

Methods and analysis

This is a multicentre, randomised (1:1) feasibility study with a mixed-methods process evaluation. Sixty participants will be recruited from TIA clinics or stroke wards at three hospital sites (England). Intervention arm participants will be offered a nurse or allied health professional-led follow-up appointment four weeks after TIA/minor stroke. The multifaceted intervention includes: a needs checklist, action plan, resources to support management of needs, a general practitioner letter, and training to deliver the intervention. Control arm participants will receive usual care. Follow-up will be self-completed questionnaires (12- and 24-weeks) and a clinic appointment (24-weeks). Follow-up questionnaires will measure anxiety, depression, fatigue, health related quality of life, self-efficacy and medication adherence. Assessment of feasibility and acceptability will include quantitative process variables, structured observations of study processes, and interviews with a sub-sample of participants and site staff.

Ethics and dissemination

Favourable ethical opinion was gained from the Wales Research Ethics Committee (REC) 1 (23/02/2021, REC reference: 21/WA/0036). Study results will be published in peer-reviewed journals and presented at conferences. A lay summary and dissemination strategy will be co-designed with patient partners. The lay summary and journal publication will be distributed on social media.

Registration details

ISRCTN reference: ISRCTN39864003

For peer review only - http://bmjopen.bmj.com/site/about/guidelines.xhtml

Strengths and limitations of this study

- The multi-centre study will enable exploration of implementation of the intervention in the context of different sites.
- The process evaluation is underpinned by the National Institutes of Health's (NIH) Behavioural Change Consortium (BCC) treatment fidelity framework.
- Quantitative and qualitative methods will explore acceptability and how the intervention is implemented in practice.
- Participants must have the ability to converse in everyday English and read in English to participate, which may limit the generalisability of our findings.

For peer review only

Introduction

Transient ischaemic attack (TIA) and minor stroke are important risk factors for stroke. Over 46,000 people experience a first TIA or minor stroke per year and 510,000 people live with TIA and minor stroke in the United Kingdom.(1)

National guidelines promote long-term management that focuses on stroke prevention.(2-4) However, research shows TIA and minor stroke patients feel unsupported in stroke prevention – both medication and lifestyle change – and often lack basic understanding of their diagnosis, stroke risk and preventative medication.(5) Furthermore, many people experience residual impairments and unmet needs after TIA or minor stroke, including fatigue, psychological and cognitive problems.(6-9) Follow-up care is variable and often inadequate with patients feeling abandoned after hospital discharge.(5)

Care needs after TIA and minor stroke include information provision (diagnosis and stroke risk); stroke prevention (medication and lifestyle change); and holistic care (residual problems and return to work or usual activities).(5) However, there is no evidence for how to best support these patients after rapid specialist review in hospital. To address this, we developed a multifaceted intervention which aims to actively identify and address unmet needs after TIA and minor stroke: SUPPORT TIA (Structured follow-Up Pathway to imProve management Of Residual impairmenTs and patients' quality of life after Transient Ischaemic Attack and minor stroke). The components of the intervention are described in this protocol. In accordance with the Medical Research Council (MRC) guidance on developing and evaluating complex interventions,(10) we will evaluate the feasibility and acceptability of (i) the intervention and (ii) a randomised controlled trial (RCT) of this intervention. In addition, we will conduct a process evaluation to evaluate intervention fidelity and contextual influences on delivery.

Methods and analysis

Study design

The study is a multicentre, individual randomised feasibility study with a mixed-methods process evaluation. The trial is registered on the ISRCTN registry (Reference: ISRCTN39864003). The study design is summarised in Figure 1.

Patient and public involvement

A core group of three people who have experienced TIA or minor stroke have supported this study from inception, with *ad hoc* contributions from other members of the public with TIA or minor stroke. The group supported the initial development of the research question and funding application, which were informed by their priorities and experiences. The group was involved in: selection of outcome measures; development of study documents; and design of the trial, such as recruitment strategies and considering participant burden related to data collection and attending intervention appointments. The group was integral to the intervention development, in particular the website of support services and resources. The group will continue to support the delivery of the study and dissemination of findings. One member (PC) is a co-author and member of the Study

For peer review only - http://bmjopen.bmj.com/site/about/guidelines.xhtml

Oversight Committee. Patient and public involvement activities and impact will be reported using GRIPP2.(11)

Study objectives

Trial design and methods

a) Assess feasibility and acceptability of the trial design and methods, including: number of patients meeting eligibility criteria; consent and randomisation processes; recruitment and retention rates; piloting the health economics questionnaire; and data completeness;
b) Provide data to inform the sample size for a definitive randomised controlled trial (RCT);
c) Provide data to help inform selection of the primary outcome measure for a definitive RCT, including data completeness and correlation of the outcome measures with each other.

Intervention (process evaluation)

d) Investigate acceptability of the intervention for participants and intervention providers;
e) Test hypotheses relating to the theoretical underpinning of the intervention;
f) Assess if intervention providers are adequately trained to deliver the intervention;
g) Assess adherence to the intervention;
h) Assess contamination with the control group;
i) Define the "dose" of the intervention;
j) Explore how well intervention participants received and understood the intervention;
k) Explore to what extent the intervention was enacted as intended by patient participants (intervention group).

Study setting, eligibility and recruitment

Participants will be recruited from TIA clinics and stroke wards at three hospital sites in England. Participants will be adults who have experienced a first or recurrent TIA or minor stroke. The full eligibility criteria are detailed in Box 1.

Box 1: Eligibility criteria

Inclusion criteria
- Adults (aged ≥18 years);
- Resident in England;
- Diagnosis of confirmed TIA or minor stroke by a stroke consultant. TIA will be defined as a transient episode of neurological dysfunction caused by focal brain, spinal cord, or retinal ischemia, without acute infarction. Minor stroke will be defined as a modified Rankin scale score ≤1 or no change in modified Rankin scale score from pre-event (to account for people who were disabled prior to their TIA or minor stroke);
- Attending the TIA clinic or stroke ward for a new diagnosis of TIA or minor stroke, rather than for a follow-up appointment;
- Ability to converse in everyday English and read in English;
- Capacity to provide fully informed consent for participation in the trial.

Exclusion criteria
- History of full stroke;
- History of dementia;

For peer review only - http://bmjopen.bmj.com/site/about/guidelines.xhtml
For peer review only

- People who lack capacity to participate, such as if they have severe memory problems that mean they would not remember giving consent or if they have severe communication problems, not precluding patients who use electronic devices to communicate;
- Patients receiving early supported discharge or cardiac rehabilitation;
- Patients receiving any palliative care.

A member of the clinical team will screen patients' medical records and approach potentially eligible patients face-to-face or by phone. After confirming eligibility, potential participants will be invited to take part in the study. Informed consent may be taken face-to-face (for people approached in clinic), by post (for people who need more time to consider participation) or verbally (for people approached via phone). Verbal consent will be clearly documented in the participant's medical records and the participant will also be sent a postal consent form to compete.

Sample size

The study will aim to recruit 60 participants (30 in the intervention group, 30 in the control group). As this is a feasibility study, no formal sample size calculation has been performed.

Randomisation

Participants will be randomised 1:1 to either the intervention or control group. A minimisation algorithm will be used within an online randomisation system to ensure balance in the treatment allocation using the following variables: age at consent (<60 years, ≥60 years); sex (male, female); diagnosis (TIA, minor stroke); employment (employed, non-employed/retired).

A 'random element' will be included in the minimisation algorithm, so that each patient has a probability, of being randomised to the opposite treatment that they would have otherwise received.

Participants will be randomised at baseline; however, to prevent baseline patient reported outcomes being affected by study arm allocation, participants will be notified of their randomisation allocation after they have returned the 1-week questionnaire or at 3 weeks (if the 1-week questionnaire is not returned).

Intervention

Intervention development was underpinned by the Behaviour Change Wheel theoretical framework(12) and iteratively refined in collaboration with patient partners and a multidisciplinary team (Appendix 3, eTable 2)

The multifaceted intervention broadly comprises six components (Figure 2):

1. Training for nurses and allied health professionals (AHPs) delivering the intervention;
2. Structured nurse or AHP led follow-up appointment, 4 weeks after TIA or minor stroke;
3. Needs checklist completed by participants prior to the appointment;
4. Resources to support management of needs, including a website of resources and support services; list of local support services; and a self-management booklet;
5. Action plan;
6. Structured letter to GPs to improve the interface communication between secondary and primary care.

For peer review only - http://bmjopen.bmj.com/site/about/guidelines.xhtml

Participants will also receive usual care and a Stroke Association TIA information sheet.

Details of the intervention are described below in accordance with the template for intervention description and replication (TIDieR) checklist.(13) The logic model is depicted in Table 1.

Table 1: Logic model

Intervention component	Mechanism of change	Outputs	Potential outcomes
**1. Training for nurses or AHPs delivering the intervention.**	HCPs educated about potential needs after TIA or minor stroke, including health and social consequences. HCPs instructed how to identify and address potential unmet needs, including use of intervention materials.	↑ HCPs knowledge of potential needs and strategies to identify and address needs.	
**2. Structured nurse or AHP led follow-up appointment, 4 weeks after TIA or minor stroke.**	Service to provide patients with access to nurse or AHP follow-up.	Patients access holistic care and support for needs related to their TIA or minor stroke. Needs may be actioned immediately (e.g. driving information, reassurance) or an agreed plan for self-management or further support.	***Short term***<br>• ↑ satisfaction with care.<br>• Access relevant further support (e.g. GP, support service).<br>• Access relevant resources (e.g. websites, apps).<br>• ↑ knowledge and understanding of diagnosis and stroke risk.
**3. Needs checklist completed by participants prior to the appointment.**	Checklist provides patients with the opportunity to reflect on their needs and facilitates communication with the HCP.	Patients' needs are actively identified and acknowledged by the HCP.	***Medium term***<br>• ↑ medication adherence.<br>• ↑ confidence and ability to self-manage needs.
**4. Resources to support management of needs, including a website of resources and support services; list of local support services; and a self-management booklet.**	↑ knowledge of relevant support services and resources.	Patients are referred or signposted to relevant support services. Patients are recommended relevant resources for their individual needs (e.g. websites, apps).	***Long term***<br>• ↑ health related quality of life.<br>• ↓ or improved residual problems

For peer review only

For peer review only - http://bmjopen.bmj.com/site/about/guidelines.xhtml

**5. Action plan.**	Provides an opportunity for shared decision making and goal setting, and empowers patient to access services or resources and/or self-manage.	Patients are given clear actions to self-manage needs or access further support.	(e.g. anxiety, fatigue).<br>• ↓ in stroke risk factors.
**6. Structured GP letter**	↑ communication between secondary and primary care, and patients and GPs. Patients are empowered to access GP support.	GPs have better understanding of their patients' needs and care received. GPs receive clear recommendations for how to further support patient needs.	

AHP: Allied Health Professional; GP: General Practitioner; HCP: Healthcare provider; TIA: Transient Ischaemic Attack; ↑: increase; ↓: decrease

Materials and procedures

Participants randomised to receive the intervention will be invited to a nurse/AHP-led follow-up appointment. Prior to their appointment, participants will be asked to complete a needs checklist. The checklist comprises 12 potential needs which encompass information provision (diagnosis and stroke risk); secondary stroke prevention (medication and lifestyle change); and holistic care (psychological and psychosocial) (online supplementary appendix 1). The checklist is an adapted version of the Stroke Review Checklist(14) and was informed by the literature and earlier qualitative research,(5) co-designed with our patient partners.

The nurse/AHP will use the checklist to guide discussions to identify participants' unmet needs. If multiple needs are identified, priority will be given to addressing needs which the participant considers the most significant.

The nurse/AHP will address needs that can be resolved during the appointment, such as information about driving. For needs that cannot be immediately addressed, the nurse/AHP will, where appropriate, refer or signpost to support services and develop an action plan which will be agreed with the participant. To facilitate this, the intervention provider will be provided with a website of resources and support services and a list of local services.

The nurse/ AHP will take the participants' blood pressure and, if raised (≥140/90 mmHg), request for the participant's GP to review blood pressure in the action plan and GP letter.

If necessary, the nurse/AHP may invite the participant to attend another follow-up appointment, at a suitable time point, to monitor the participant's progress and revise the action plan if required. These additional follow-ups may be conducted by telephone, video call or face-to-face.

A letter will be sent to the participant's GP along with a copy of the agreed action plan. A letter template will include recommended GP actions, a summary of the appointment and actions taken.

The participant will be provided with a self-management booklet (an abridged version of the resources and services website) and a copy of the action plan and GP letter.

For peer review only - http://bmjopen.bmj.com/site/about/guidelines.xhtml

Intervention provider

The intervention will be delivered by a nurse or AHP with stroke expertise. The nurses and AHPs will attend training which will include education about potential needs after TIA and minor stroke, and how to deliver the intervention.

Setting and modes of delivery

The intervention appointments will be delivered at the site's TIA clinic, either face-to-face or remotely (e.g. telephone or video call). Face-to-face delivery will be preferable where possible.

When and how often

The intervention appointment will take place at four weeks (or up to six weeks). The appointment is expected to last approximately 30 minutes. One appointment will be offered initially; however, participants will have an option to attend additional follow-up if judged clinically necessary by the nurse or AHP.

Control arm

The control group will receive usual care and be given a Stroke Association TIA information sheet when they are informed about their allocation to the control arm.

Outcomes and data collection

Definitive RCT

Table 1 summarises the proposed patient reported, health economic and clinical outcome measures for a definitive RCT. Contact details, demographic information and medical history will be collected at baseline. Questionnaires comprising Patient Reported Outcome Measures (PROMs) (Table 2) will be completed by participants, either by post or electronically, at 1, 12 and 24 weeks. PROM rationale for assessment and psychometric properties are presented in eTable 1, see appendix 2. Questionnaires at 12 and 24 weeks will also collect health economics data. The first PROM completion will be at one week rather than baseline due to the nature of the PROM questions and to reduce burden on participants. Clinical data (Table 2) will be collected at an end of study clinic appointment at 24 weeks.

Table 2: summary of outcome measures for the main RCT.

	Data	Timepoint
**Baseline data**	Contact details<br>Demographic: date of birth, sex, ethnicity, employment status<br>Medical: diagnosis, date of TIA or minor stroke, modified Rankin scale score, length of stay, smoking status, alcohol consumption, height, weight, body mass index, comorbidities, medication, blood pressure, cholesterol	Baseline
**Patient reported outcome measure**	Health related quality of life: Patient-Reported Outcomes Measurement Information System (PROMIS)-Global Health 10<br>Health related quality of life: 5-level EuroQol 5-Dimensions (EQ-5D-5L)	1, 12 and 24 weeks

For peer review only - http://bmjopen.bmj.com/site/about/guidelines.xhtml

For peer review only

	Anxiety/ depression: Hospital Anxiety and Depression Scale (HADS)<br>Fatigue: Fatigue Assessment Scale (FAS)<br>Self-efficacy: Patient Activation Measure-13 (PAM-13)<br>Medication adherence: Medication Adherence Rating Scale -5 (MARS)<br>Satisfaction with care question	
**Health economics**	Use of healthcare services<br>Change in employment status, altered work hours and days off sick<br>Other costs incurred because of TIA or minor stroke	12 and 24 weeks
**Clinical data**	Body Mass Index<br>Blood pressure<br>Bloods: cholesterol<br>Medications	Baseline and 24 weeks

Feasibility study and process evaluation

The feasibility study and process evaluation outcomes are detailed in Tables 3 and 4. The process evaluation is underpinned by the National Institutes of Health's (NIH) Behavioural Change Consortium (BCC) treatment fidelity framework.(15) This framework includes five domains of treatment fidelity: Study Design, Training, Delivery, Receipt and Enactment.

Table 3: Feasibility outcomes and measurement of outcomes

Objective	Feasibility outcomes	Measurement of outcome
a) Assess feasibility and acceptability of the trial design and methods	Number of eligible/ineligible patients and reasons for ineligibility	Recruitment log
	Proportion of participants who consent face-to-face, verbal or postal	Registration log: method of consent
	Willingness of clinical staff to randomise patients	Interviews (clinical staff)
	Recruitment and attrition rates	Registration log
	Response rates and frequencies of missing data: participant completed questionnaires and case report forms	1, 12 and 24 week questionnaires<br>Case Report forms
	End of study clinic appointment attendance rates	End of Study Clinic Appointment Form
	Acceptability of the trial design	Interviews (participants and clinical staff)<br>Structured observations
b) Provide data to inform the sample size for a definitive randomised controlled trial	Standard deviations of continuous patient reported outcome measures at six months	Patient reported outcome measure scores
	Recruitment and attrition rates	Registration log

For peer review only - http://bmjopen.bmj.com/site/about/guidelines.xhtml

c) Provide data to help inform selection of the primary outcome measure for a definitive randomised controlled trial	Correlation of patient reported outcome measures	Patient reported outcome measure scores
	Patient reported outcome measure response rates and missing data	1, 12 and 24 week questionnaires

Table 4: Process evaluations outcomes and measurement of outcomes

NIH BCC domain	Objective	Outcome	Measurement of outcome
**Study design**	d) Investigate acceptability of the intervention for participants and intervention providers	Participants' and clinical staff's opinion on acceptability of the intervention	Interviews (participants and clinical staff)<br>Feedback questionnaire (intervention participants)
	e) Test hypotheses relating to the theoretical underpinning of the intervention	Participants' satisfaction with identification and management of needs	Interviews (participants and clinical staff)<br>Feedback questionnaire (intervention participants)
		Participants acting on agreed action plans and/or accessing support services	Interviews (participants)
**Training**	f) Assess if intervention providers are adequately trained to deliver the intervention	Intervention providers' understanding of the intervention components	Interviews (clinical staff)
**Delivery**	g) Assess adherence to the intervention	Intervention providers' adherence to and deviations from the intervention manual	Structured observations<br>Intervention log
	h) Assess contamination with the control group	Control group contamination	Interviews (participants and clinical staff)<br>Structured observations
	i) Define the "dose" of the intervention	Intervention follow-up appointment: attendance, length of appointment and number of appointments	Intervention log
**Receipt**	j) Explore how well intervention participants received and understood the intervention	Participants' perception of the intervention	Interviews (participants)<br>Feedback questionnaire (intervention participants)
**Enactment**	k) Explore to what extent the intervention was enacted as intended	Participants acting on agreed action plans and/or accessing support services	Interviews (intervention participants)

For peer review only - http://bmjopen.bmj.com/site/about/guidelines.xhtml

	by intervention participants

Case report forms

The following case report forms will collect data on feasibility outcomes: recruitment log (recruitment rates and reasons for ineligibility); registration log (method of consent: face-to-face/ verbal/ postal); intervention log (attendance rates, duration, number of appointments per participant); end of study clinic appointment form (attendance). Case report forms will be assessed for missing data.

Participant completed questionnaires

Participant completed questionnaires (1, 12 and 24 weeks) will be analysed for response rates and missing data. Standard deviations of continuous PROMs at six months and correlation of PROMs will inform the sample size and selection of outcome measures for the definitive RCT. The intervention feedback questionnaire will report acceptability of the intervention.

Structured observations

A member of the study team will observe the following study processes: recruitment and consent procedures; intervention appointments; and end of study clinic appointments. Both face-to-face and remote modes of delivery will be observed for these procedures if possible. Approximately three observations will be conducted for each of these procedures (one at each site). A pragmatic approach will be taken to select which sessions to observe based on the availability of the research and clinical teams. A checklist will be used to document adherence to the protocol and field notes will be collected.

Audit

At the end of the recruitment period, each site will perform an audit to identify the total number of confirmed TIA and minor stroke patients who attended the TIA clinic or stroke ward during the recruitment period. The age and sex of these patients will also be collected. This data will be used to compare average age and sex of patients recruited to the trial against patients not recruited.

Qualitative interviews

At the end of the study, semi-structured interviews will be conducted with a subset of participants and clinical staff involved in recruitment and/or intervention delivery. The sample size is anticipated to be 8-10 patients and 4-6 clinical staff. For patient participants, convenience sampling will be used initially; however, sampling will become increasingly purposeful to achieve variation in age (<60 years, ≥60 years) and diagnosis (TIA, minor stroke). For clinical staff, convenience sampling will be used. Interviews will explore acceptability of the intervention and trial design.

Analysis

Quantitative outcomes will be analysed using simple descriptive statistics (e.g. proportions and percentages, mean and standard deviations) and where appropriate, point estimates of effect sizes (e.g. mean differences and relative risks) and associated 95% confidence intervals. Analysis will be conducted using Stata v16.

For peer review only - http://bmjopen.bmj.com/site/about/guidelines.xhtml

For qualitative data, NVivo v12 will be used to manage, sort, code and organise the anonymised transcribed data. Interview transcripts will be analysed using directed thematic analysis informed by the research aims.(16)

The health economics analysis will assess completion rates, estimate resources required to deliver the intervention and report simple descriptive statistics for resource use and outcomes. Key resource use items not currently specified on the form but included by participants will also be identified. The information will inform the cost and outcome data collection and identification of unit costs for a larger trial.

Data will be made available upon reasonable request.

Progression criteria

The pre-defined progression criteria, detailed in Table 5, will be used to inform a decision on whether a full RCT is warranted and feasible. The criteria were agreed by the Study Oversight Committee and follow a traffic light system using quantitative measures supported by qualitative data.

Table 5: Progression criteria

Key uncertainties	Measures used	Progression criteria
**Trial design**		
Recruitment	% target sample size recruited	● ≥90%: proceed to a full-scale trial<br>● 70-89%: SOC will consider the feasibility of proceeding to a full-scale trial bearing in mind the data presented, representativeness of the sample, and possible steps to increase recruitment<br>● <70%: full-scale trial unlikely to be feasible
Randomisation*	% of consented participants randomised	● ≥90%: proceed to a full-scale trial<br>● 70-89%: SOC will consider the feasibility of proceeding to a full-scale trial bearing in mind the data presented, representativeness of the sample, and possible steps to address randomisation issues<br>● <70%: full-scale trial unlikely to be feasible
Return rate of 24-week questionnaire*	% of 24-week questionnaires returned	● ≥80%: proceed to a full-scale trial<br>● 50-79%: SOC will consider the feasibility of proceeding to a full-scale trial bearing in mind the data presented, representativeness of the sample, and possible steps to increase return rates<br>● <50%: full-scale trial unlikely to be feasible
**Intervention**		
Attendance	% of intervention arm	● ≥90%: proceed to a full-scale trial

For peer review only - http://bmjopen.bmj.com/site/about/guidelines.xhtml

rate*	participants attending first appointment	70-89%: SOC will consider the feasibility of proceeding to a full-scale trial bearing in mind the data presented, representativeness of the sample, and possible steps to increase attendance<br><70%: full-scale trial unlikely to be feasible
Delivery of the intervention	% completion of: checklists, action plans, GP letters; use of directory of support services; Issues regarding delivery of the intervention components and contamination explored in qualitative interviews	The SOC will consider the quantitative and qualitative data and make an overall judgement on whether the intervention content is delivered as intended
Acceptability	% of participants reporting acceptability of intervention components on intervention feedback questionnaire; Issues regarding acceptability of the intervention components explored in qualitative interviews	The SOC will consider the quantitative and qualitative data and make an overall judgement on whether the intervention is acceptable

* critical progression criteria: the trial is unlikely to be feasible if these criteria are not met, even if other criteria are satisfactory.
GP: General Practitioner; SOC: Study Oversite Committee; %: percent

Ethics and dissemination

Favourable ethical opinion was gained from the Wales Research Ethics Committee (REC) 1 (23/02/2021, REC reference: 21/WA/0036). Study results will be published in a peer-reviewed journal and presented at relevant conferences. A lay summary and dissemination strategy will be co-designed with our patient partners. The lay summary and peer review publication will be distributed on social media.

Authors' contributions

GT, MC, JM, RF and LA contributed to the conception of the study. The design of the feasibility study was led by GT with contributions from MC, JM, RF, LA, PC and SP, SJ and ST. The design of the intervention was led by GT with contributions from MC, JM, RF, LA, PC and RJ. GT wrote the first draft of the manuscript. All authors contributed to and approved the final version.

Acknowledgments

We thank the patient and public involvement group members who have provided valuable insight and feedback into the intervention and study design: Phil Collis, Lesley Thomson and Sally Hughes. We thank those involved in the development of the intervention with special mention to Kirsty Price and Joanne Chesson.

For peer review only - http://bmjopen.bmj.com/site/about/guidelines.xhtml

Funding statement

This work was supported by National Institute for Health Research (NIHR) Post-Doctoral Fellowship Scheme grant number PDF-2017-10-047. The views expressed are those of the author(s) and not necessarily those of the NIHR or the Department of Health and Social Care.

Competing interests

MC and JM are National Institute for Health Research (NIHR) Senior Investigators. MC also receives funding from the National Institute for Health Research (NIHR), UK Research and Innovation (UKRI), the NIHR Birmingham Biomedical Research Centre, NIHR Surgical Reconstruction and Microbiology Research Centre and NIHR ARC West Midlands at the University of Birmingham and University Hospitals Birmingham NHS Foundation Trust, UK SPINR (UKRI) Health Data Research UK, Innovate UK (part of UKRI), Macmillan Cancer Support, UCB Pharma, GSK, Gilead and Janssen. MC has received personal fees from Astellas, Aparito Ltd, CIA Oncology, Takeda, Merck, Daiichi Sankyo, Glaukos, GSK and the Patient-Centered Outcomes Research Institute (PCORI) outside the submitted work. The views expressed in this publication are those of the author(s) and not necessarily those of the NIHR or the Department of Health and Social Care. JM has done consultancy work for BMS/Pfizer, Omron, and Doctorlink Innovations Ltd. GMT, RJ, PC, SP, SJ, ST, RF and LA declare no conflict of interest.

References

1. Scarborough P, Peto V, Bhatnagar P, Kaur A, Leal J, Luengo-Fernandez R, et al. Stroke statistics. British Heart Foundation and Stroke Association: London; 2009.
2. European Stroke Organisation (ESO) Executive Committee. Guidelines for management of ischaemic stroke and transient ischaemic attack 2008. Cerebrovascular diseases (Basel, Switzerland). 2008;25(5):457-507.
3. Intercollegiate Stroke Working Party. National clinical guideline for stroke. London: Royal College of Physicians; 2016.
4. Kernan WN, Ovbiagele B, Black HR, Bravata DM, Chimowitz MI, Ezekowitz MD, et al. Guidelines for the Prevention of Stroke in Patients With Stroke and Transient Ischemic Attack. Stroke. 2014;45(7):2160-236.
5. Turner GM, McMullan C, Atkins L, Foy R, Mant J, Calvert M. TIA and minor stroke: a qualitative study of long-term impact and experiences of follow-up care. BMC Family Practice. 2019;20(1):176.
6. Moran GM, Fletcher B, Feltham MG, Calvert M, Sackley C, Marshall T. Fatigue, psychological and cognitive impairment following transient ischaemic attack and minor stroke: a systematic review. European journal of neurology. 2014;21(10):1258-67.
7. Sackley CM, Mant J, McManus RJ, Humphreys G, Sharp L, Mares K, et al. Functional and emotional outcomes after transient ischemic attack: A 12-month prospective controlled cohort study. International journal of stroke. 2019:1747493018823158.
8. Turner GM, Calvert M, Feltham MG, Ryan R, Marshall T. Ongoing impairments following transient ischaemic attack: retrospective cohort study. European journal of neurology. 2016;23(11):1642-50.
9. van Rooij FG, Kessels RP, Richard E, De Leeuw FE, van Dijk EJ. Cognitive Impairment in Transient Ischemic Attack Patients: A Systematic Review. Cerebrovascular diseases (Basel, Switzerland). 2016;42(1-2):1-9.

For peer review only - http://bmjopen.bmj.com/site/about/guidelines.xhtml

10. Skivington K, Matthews L, Simpson SA, Craig P, Baird J, Blazeby JM, et al. A new framework for developing and evaluating complex interventions: update of Medical Research Council guidance. BMJ. 2021;374:n2061.
11. Staniszewska S, Brett J, Simera I, Seers K, Mockford C, Goodlad S, et al. GRIPP2 reporting checklists: tools to improve reporting of patient and public involvement in research. BMJ. 2017;358:j3453.
12. Michie S, van Stralen MM, West R. The behaviour change wheel: A new method for characterising and designing behaviour change interventions. Implementation Science. 2011;6(1):42.
13. Hoffmann TC, Glasziou PP, Boutron I, Milne R, Perera R, Moher D, et al. Better reporting of interventions: template for intervention description and replication (TIDieR) checklist and guide. BMJ : British Medical Journal. 2014;348:g1687.
14. Turner GM, Mullis R, Lim L, Kreit L, Mant J. Using a checklist to facilitate management of long-term care needs after stroke: insights from focus groups and a feasibility study. BMC Family Practice. 2019;20(1):2.
15. Bellg AJ, Borrelli B, Resnick B, Hecht J, Minicucci DS, Ory M, et al. Enhancing treatment fidelity in health behavior change studies: best practices and recommendations from the NIH Behavior Change Consortium. Health Psychol. 2004;23(5):443-51.
16. Fereday J, Muir-Cochrane E. Demonstrating Rigor Using Thematic Analysis: A Hybrid Approach of Inductive and Deductive Coding and Theme Development. International Journal of Qualitative Methods. 2006;5(1):80-92.

Figure legends

Figure 1: Trial schema

Figure 2: Summary of the intervention components

For peer review only

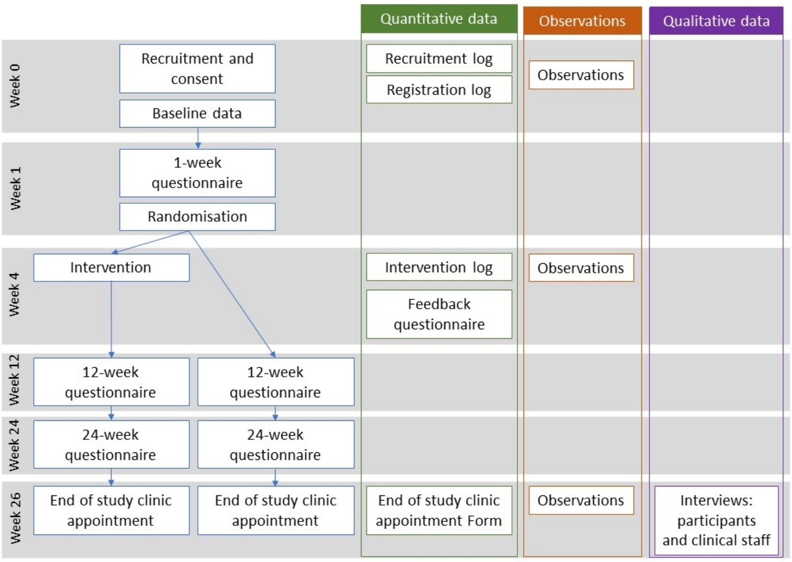

Figure 1

338x190mm (96 x 96 DPI)

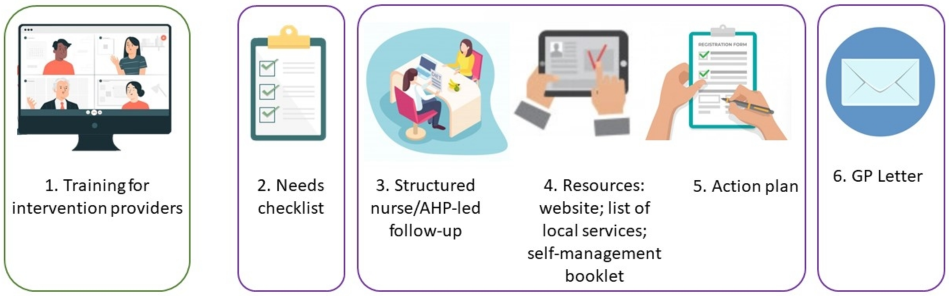

This figure has been designed using resources from Freepik.com

Figure 2

338x190mm (96 x 96 DPI)

TNO | | | | |

My **information** and **support needs** after mini-stroke

This is a list of needs that some people have after mini stroke.

Please tick (✓) any areas you have difficulty with, even if you have only experienced it occasionally.

Your follow-up appointment is an opportunity to discuss any worries or problems you might be experiencing, and any advice or information you may need following your mini stroke (TIA) or minor stroke. Please use this list to help you to prepare for your appointment.

Information

1. I need more information about:	*Tick here*
• My diagnosis	
• My risk of stroke	
• Driving after my mini stroke	

Stroke prevention

2. I need advice on:	*Tick here*
• Medications for preventing another stroke	
• Medication side effects	
• Lifestyle change, such as exercise or diet, to prevent another stroke	

Effects of mini-stroke

3. Fatigue	*Tick here*
• I feel tired most of the time or I get easily tired	
• I find it difficult to concentrate and do things	
**4. Mood**	
• I feel anxious	
• I feel depressed	
• I experience anger, frustration or mood swings	
• I feel that my personality has changed	
**5. Memory and thinking**	
• I find it difficult to think, concentrate, or remember things	

For peer review only - http://bmjopen.bmj.com/site/about/guidelines.xhtml

TNO | | | |

6. Communication	
• I finding it difficult to understand / communicate with others	
• I have problems with speech, word finding or talking to others	
**7. Physical**	
• I experience muscle weakness or problems with balance	
• I have headaches	
• I am sensitive to noise or light	
**8. Incontinence**	
• I am having a problem controlling my bladder or bowels	
**9. Intimate relationships**	
• Since my mini stroke I have problems with sex	
**10. Work or education**	
• I am having problems at work or education	
• I would like support and advice on returning to work or education	
**11. Relationships with family or friends**	
• My personal relationships with my family or friends have become difficult or stressed	
**12. Social activities or daily tasks**	
• I find it difficult to take part in hobbies or leisure activities	
• I have difficulty doing daily tasks	

Which of the needs you identified above is your biggest concern?

Is there anything else you are concerned about or would like more information about?

Please bring this list to your follow-up appointment

For peer review only - http://bmjopen.bmj.com/site/about/guidelines.xhtml

eTable 1: Summary of proposed PROMs for the main RCT.

Domain	PROM	Summary	Rationale
Health related quality of life	PROMIS-Global Health 10	**Questions:** 10<br>**Scoring:** 5-point Likert + 10 point Likert pain question. Scored using item-level calibrations- use the Health Measures Scoring Service (https://www.assessmentcenter.net/ac_scoringservice)<br>**MID:** none specified<br>**Permissions:** publicly available for use	Recommended by the International Consortium for Health Outcomes Measurement (2016) as part of a consensus stroke measure standard set (25)
Health related quality of life	EQ-5D-5L	**Questions:** 5 + 1 VAS (20 cm)<br>**Scoring:** 5-point Likert scale and VAS. Score = 0-1<br>**MID:** for stroke: 0.08 to 0.12 (26)<br>**Permissions:** licence agreement needed (€600)	Used to calculate Quality Adjusted Life Years for health economics analyses
Anxiety/ depression	HADS	**Questions:** 14<br>**Scoring:** 4-point Likert Scale. 0-7 = Normal; 8-10 = Borderline abnormal; 11-21 = Abnormal<br>**MID:** none specified<br>**Permissions:** license agreement needed (0-1000 participants= £0.85 each)	Widely used in TIA/ minor stroke research, comprehensive psychometric evaluation
Fatigue	FAS	**Questions:** 10<br>**Scoring:** 5-point Likert Scale; Score = 10 (lowest fatigue) to 50 (highest fatigue). 10-21: no fatigue (normal), 22-50: substantial fatigue<br>**MID:** at least 4 points or 10% change of the baseline value<br>**Permissions:** must acknowledge the ild care foundation in the manuscript, FAS needs to be added as a keyword in the final publication, and PDF of the final publication must be send to the ild care foundation: info@ildcare.nl	Systematic review of fatigue scales for stroke scales identified the FAS had the best test-retest reliability(27)
Self-efficacy	PAM-13	**Questions**: 13<br>**Scoring:** 5-point Likert Scale; Score 1-100: 4 Levels: Level 1 ≤47.0 (not believing activation is important), Level 2 47.1–55.1 (a lack of knowledge and confidence to take action), Level 3 55.2–67.0 (beginning to take action), level 4 ≥67.1 (taking action)<br>**MID:** not specified<br>**Permissions:** licence agreement NOT needed for feasibility studies	Used by NHS England
Medication adherence	MARS-5	**Questions:** 6<br>**Scoring:** 5-point Likert scale, score= 5 to 25 (higher scores= higher self-reported adherence)<br>**MID:** not specified<br>**Permissions:** Free access (subject to adequate citation) for Academic users	Validated in stroke patients, widely used
Satisfaction with care	Satisfaction with care question	Not applicable- non-validated bespoke question	

For peer review only

Summary of intervention development, underpinned by the theoretical framework - Behaviour Change Wheel

- **Select target behaviour:** Healthcare provider identifying and addressing needs post-TIA/ minor stroke.
- **Identify what needs to change:**
  - Semi-structured interviews were conducted with healthcare providers and patients to identify influences on the target behaviour (results reported elsewhere). Transcripts were coded using the Theoretical Domains Framework (TDF).
- **Map TDF domains to "intervention functions"**
  - Four relevant intervention function identified: Education, Training, Environmental restructuring, Enablement.
- **Identify Behaviour Change Techniques (BCT):**
  - The BCT Taxonomy was used to identity appropriate BCT that mapped to relevant intervention functions.

The above process was informed by relevant literature; iterative feedback from patient partners and a multidisciplinary team (nurses, allied health professionals, GPs, consultants, researcher); and consideration of the APEASE criteria (Affordability, Practicality, Effectiveness and cost-effectiveness, Acceptability, Side-effects/safety, and Equity).

eTable 2: Summary of the barriers/ enablers mapped to Theoretical Domain Framework domains, intervention functions, Behaviour Change Taxonomies and intervention components.

Barriers (B)/ Enablers (E)	TDF	BCW intervention function	BCT	SUPPORT TIA Intervention component
HCPs' lack of knowledge of potential needs post-TIA/ minor stroke (B).<br><br>HCPs' perceived role in follow-up care influenced their approach to identifying and addressing needs (B/E).	Knowledge<br><br>Social professional role and identity<br><br>Goal	Education/ Training	*Information about health consequences*<br><br>*Information about social and environmental consequences*<br><br>*Information about emotional consequences*<br><br>*Instruction on how to perform the behaviour*<br><br>*Identity associated with changed behaviour*	Training for intervention providers

For peer review only

Lack of follow-up pathway following rapid hospital specialist review (B).<br><br>Time constraints (B).<br><br>Nurses and AHPs were more holistic in their approach to care (compared to doctors) and considered this part of their role (E).	Environmental context and resources<br><br>Social professional role and identity<br><br>Intentions	Environmental restructuring	*Restructure physical environment*	Structured nurse/AHP-led follow-up appointment
Checklists considered useful prompts to identify needs (E).		Environmental restructuring/ Enablement	*Prompt/ cues*	Needs checklist completed by participants prior to the appointment
HCPs' lack of knowledge of support services and resources to support needs (B).<br><br>Directories of support services facilitated identification of support services (if up-to-date) (E).	Knowledge<br><br>Environmental context and resources	Environmental restructuring/ Enablement	*Instruction on how to perform the behaviour*<br><br>*Adding objects to the environment*	Resources to support management of needs, including a website of resources and support services; list of local support services; and a self-management booklet
Patients feel unsupported after hospital (B).<br><br>Difficult for patients to process and retain information (B).<br><br>Patients attempted to access information and support themselves, but found it overwhelming, confusing, contradictory (B).	Social influences	Environmental restructuring/ Enablement	*Action planning*<br><br>*Goal setting*<br><br>*Problem solving*<br><br>*Pharmacological support*	Action plan
Restricted communication between primary and secondary care (B).<br><br>Variability in the speed and content of GP letters (B/E)	Environmental context and resources<br><br>Social influences	Environmental restructuring/ Enablement	*Action planning*<br><br>*Prompts/cues*	Structured GP letter

For peer review only

SPIRIT

STANDARD PROTOCOL ITEMS: RECOMMENDATIONS FOR INTERVENTIONAL TRIALS

SPIRIT 2013 Checklist: Recommended items to address in a clinical trial protocol and related documents*

Section/item	Item No	Description	Page
**Administrative information**			
Title	1	Descriptive title identifying the study design, population, interventions, and, if applicable, trial acronym	1
Trial registration	2a	Trial identifier and registry name. If not yet registered, name of intended registry	3
	2b	All items from the World Health Organization Trial Registration Data Set	n/a
Protocol version	3	Date and version identifier	n/a
Funding	4	Sources and types of financial, material, and other support	15
Roles and responsibilities	5a	Names, affiliations, and roles of protocol contributors	1
	5b	Name and contact information for the trial sponsor	1
	5c	Role of study sponsor and funders, if any, in study design; collection, management, analysis, and interpretation of data; writing of the report; and the decision to submit the report for publication, including whether they will have ultimate authority over any of these activities	15
	5d	Composition, roles, and responsibilities of the coordinating centre, steering committee, endpoint adjudication committee, data management team, and other individuals or groups overseeing the trial, if applicable (see Item 21a for data monitoring committee)	REC protocol
**Introduction**			
Background and rationale	6a	Description of research question and justification for undertaking the trial, including summary of relevant studies (published and unpublished) examining benefits and harms for each intervention	5
	6b	Explanation for choice of comparators	9
Objectives	7	Specific objectives or hypotheses	5

For peer review only

Trial design	8	Description of trial design including type of trial (eg, parallel group, crossover, factorial, single group), allocation ratio, and framework (eg, superiority, equivalence, noninferiority, exploratory)	5
**Methods: Participants, interventions, and outcomes**			
Study setting	9	Description of study settings (eg, community clinic, academic hospital) and list of countries where data will be collected. Reference to where list of study sites can be obtained	6
Eligibility criteria	10	Inclusion and exclusion criteria for participants. If applicable, eligibility criteria for study centres and individuals who will perform the interventions (eg, surgeons, psychotherapists)	6
Interventions	11a	Interventions for each group with sufficient detail to allow replication, including how and when they will be administered	7-9
	11b	Criteria for discontinuing or modifying allocated interventions for a given trial participant (eg, drug dose change in response to harms, participant request, or improving/worsening disease)	n/a
	11c	Strategies to improve adherence to intervention protocols, and any procedures for monitoring adherence (eg, drug tablet return, laboratory tests)	n/a
	11d	Relevant concomitant care and interventions that are permitted or prohibited during the trial	n/a
Outcomes	12	Primary, secondary, and other outcomes, including the specific measurement variable (eg, systolic blood pressure), analysis metric (eg, change from baseline, final value, time to event), method of aggregation (eg, median, proportion), and time point for each outcome. Explanation of the clinical relevance of chosen efficacy and harm outcomes is strongly recommended	10-12
Participant timeline	13	Time schedule of enrolment, interventions (including any run-ins and washouts), assessments, and visits for participants. A schematic diagram is highly recommended (see Figure)	5 (figure 1)
Sample size	14	Estimated number of participants needed to achieve study objectives and how it was determined, including clinical and statistical assumptions supporting any sample size calculations	6
Recruitment	15	Strategies for achieving adequate participant enrolment to reach target sample size	6
**Methods: Assignment of interventions (for controlled trials)**			
Allocation:			7

Sequence generation	16a	Method of generating the allocation sequence (eg, computer-generated random numbers), and list of any factors for stratification. To reduce predictability of a random sequence, details of any planned restriction (eg, blocking) should be provided in a separate document that is unavailable to those who enrol participants or assign interventions	7
Allocation concealment mechanism	16b	Mechanism of implementing the allocation sequence (eg, central telephone; sequentially numbered, opaque, sealed envelopes), describing any steps to conceal the sequence until interventions are assigned	7
Implementation	16c	Who will generate the allocation sequence, who will enrol participants, and who will assign participants to interventions	7
Blinding (masking)	17a	Who will be blinded after assignment to interventions (eg, trial participants, care providers, outcome assessors, data analysts), and how	7
	17b	If blinded, circumstances under which unblinding is permissible, and procedure for revealing a participant's allocated intervention during the trial	7
**Methods: Data collection, management, and analysis**			
Data collection methods	18a	Plans for assessment and collection of outcome, baseline, and other trial data, including any related processes to promote data quality (eg, duplicate measurements, training of assessors) and a description of study instruments (eg, questionnaires, laboratory tests) along with their reliability and validity, if known. Reference to where data collection forms can be found, if not in the protocol	10-12
	18b	Plans to promote participant retention and complete follow-up, including list of any outcome data to be collected for participants who discontinue or deviate from intervention protocols	n/a
Data management	19	Plans for data entry, coding, security, and storage, including any related processes to promote data quality (eg, double data entry; range checks for data values). Reference to where details of data management procedures can be found, if not in the protocol	
Statistical methods	20a	Statistical methods for analysing primary and secondary outcomes. Reference to where other details of the statistical analysis plan can be found, if not in the protocol	13
	20b	Methods for any additional analyses (eg, subgroup and adjusted analyses)	n/a

	20c	Definition of analysis population relating to protocol non-adherence (eg, as randomised analysis), and any statistical methods to handle missing data (eg, multiple imputation)	n/a
**Methods: Monitoring**			
Data monitoring	21a	Composition of data monitoring committee (DMC); summary of its role and reporting structure; statement of whether it is independent from the sponsor and competing interests; and reference to where further details about its charter can be found, if not in the protocol. Alternatively, an explanation of why a DMC is not needed	REC protocol
	21b	Description of any interim analyses and stopping guidelines, including who will have access to these interim results and make the final decision to terminate the trial	n/a
Harms	22	Plans for collecting, assessing, reporting, and managing solicited and spontaneously reported adverse events and other unintended effects of trial interventions or trial conduct	REC protocol
Auditing	23	Frequency and procedures for auditing trial conduct, if any, and whether the process will be independent from investigators and the sponsor	REC protocol
**Ethics and dissemination**			
Research ethics approval	24	Plans for seeking research ethics committee/institutional review board (REC/IRB) approval	15
Protocol amendments	25	Plans for communicating important protocol modifications (eg, changes to eligibility criteria, outcomes, analyses) to relevant parties (eg, investigators, REC/IRBs, trial participants, trial registries, journals, regulators)	REC protocol
Consent or assent	26a	Who will obtain informed consent or assent from potential trial participants or authorised surrogates, and how (see Item 32)	6
	26b	Additional consent provisions for collection and use of participant data and biological specimens in ancillary studies, if applicable	n/a
Confidentiality	27	How personal information about potential and enrolled participants will be collected, shared, and maintained in order to protect confidentiality before, during, and after the trial	REC protocol
Declaration of interests	28	Financial and other competing interests for principal investigators for the overall trial and each study site	15
Access to data	29	Statement of who will have access to the final trial dataset, and disclosure of contractual agreements that limit such access for investigators	13

review only

Ancillary and post-trial care	30	Provisions, if any, for ancillary and post-trial care, and for compensation to those who suffer harm from trial participation	n/a
Dissemination policy	31a	Plans for investigators and sponsor to communicate trial results to participants, healthcare professionals, the public, and other relevant groups (eg, via publication, reporting in results databases, or other data sharing arrangements), including any publication restrictions	15
	31b	Authorship eligibility guidelines and any intended use of professional writers	REC protocol
	31c	Plans, if any, for granting public access to the full protocol, participant-level dataset, and statistical code	n/a
**Appendices**			
Informed consent materials	32	Model consent form and other related documentation given to participants and authorised surrogates	REC protocol
Biological specimens	33	Plans for collection, laboratory evaluation, and storage of biological specimens for genetic or molecular analysis in the current trial and for future use in ancillary studies, if applicable	n/a

*It is strongly recommended that this checklist be read in conjunction with the SPIRIT 2013 Explanation & Elaboration for important clarification on the items. Amendments to the protocol should be tracked and dated. The SPIRIT checklist is copyrighted by the SPIRIT Group under the Creative Commons "Attribution-NonCommercial-NoDerivs 3.0 Unported" license.

For peer review only

BMJ Open

Structured follow-up pathway to support people after transient ischaemic attack and minor stroke (SUPPORT TIA): Protocol for a feasibility study and process evaluation

Journal:	*BMJ Open*
Manuscript ID	bmjopen-2021-060280.R1
Article Type:	Protocol
Date Submitted by the Author:	22-Apr-2022
Complete List of Authors:	Turner, Grace; Institute of Applied Health Research University of Birmingham; University of Birmingham<br>Jones, Rachael; Royal Wolverhampton NHS Trust<br>Collis, Phillip; Institute of Applied Health Research University of Birmingham; University of Birmingham<br>Patel, Smitaa; University of Birmingham, Birmingham Clinical Trials Unit<br>Jowett, Sue; University of Birmingham, Health Economics Unit<br>Tearne, Sarah ; University of Birmingham Clinical Trials Unit<br>Foy, Robbie; University of Leeds, Leeds Institute of Health Sciences<br>Atkins, Lou; UCL<br>Mant, Jonathan; University of Cambridge, General Practice and Primary Care Research Unit<br>Calvert , Melanie ; University of Birmingham, Centre for Patient Reported Outcomes Research and Institute of Applied Health Research
**Primary Subject Heading**:	Cardiovascular medicine
Secondary Subject Heading:	Research methods
Keywords:	STROKE MEDICINE, REHABILITATION MEDICINE, Organisation of health services < HEALTH SERVICES ADMINISTRATION & MANAGEMENT, Protocols & guidelines < HEALTH SERVICES ADMINISTRATION & MANAGEMENT, Depression & mood disorders < PSYCHIATRY, QUALITATIVE RESEARCH

SCHOLARONE™
Manuscripts

Structured follow-up pathway to support people after transient ischaemic attack and minor stroke (SUPPORT TIA): Protocol for a feasibility study and process evaluation

Grace M Turner* 1, 2

Rachael Jones 3, 4

Phillip Collis 1, 2

Smitaa Patel 6

Sue Jowett 7

Sarah Tearne 6

Robbie Foy 8

Lou Atkins 9

Jonathan Mant 10

Melanie Calvert 1, 2, 11, 12, 13, 14

1 Institute of Applied Health Research, University of Birmingham, Birmingham, UK

2 Centre for Patient Reported Outcomes Research, University of Birmingham, Birmingham, UK

3 Royal Wolverhampton NHS Trust, Wolverhampton Rd, Heath Town, Wolverhampton, UK

4 Clinical Research Network West Midlands (CRN WM), UK

5 Patient partner, UK

6 Birmingham Clinical Trials Unit, University of Birmingham, Birmingham, UK

7 Health Economics Unit, University of Birmingham, Birmingham, UK

8 Leeds Institute of Health Sciences, University of Leeds, Leeds, UK

9 Centre for Behaviour Change, University College London, London, UK

10 Department of Public Health and Primary Care, University of Cambridge, Cambridge, UK

11 NIHR Surgical Reconstruction and Microbiology Research Centre, University Hospitals Birmingham NHS Foundation Trust and University of Birmingham, Birmingham, UK

12 NIHR Birmingham Biomedical Research Centre, University Hospitals Birmingham NHS Foundation Trust and University of Birmingham, Birmingham, UK

For peer review only

For peer review only - http://bmjopen.bmj.com/site/about/guidelines.xhtml

13 Birmingham Health Partners Centre for Regulatory Science and Innovation, University of Birmingham, Birmingham, UK

14 NIHR Applied Research Collaboration (ARC) West Midlands, University of Birmingham, Birmingham, UK

* Corresponding author: Dr Grace Turner; Institute of Applied Health Research, University of Birmingham, Birmingham, B15 2TT, UK; G.Turner.1@bham.ac.uk

Word count

2,353

Key words

Transient ischaemic attack; mini stroke; TIA; minor stroke; rehabilitation, stroke prevention, follow-up, Hospital Anxiety and Depression Scale, Patient-Reported Outcomes Measurement Information System, 5-level EuroQol 5-Dimensions, Fatigue Assessment Scale, Patient Activation Measure-13, Medication Adherence Rating Scale -5

For peer review only

Abstract

Introduction

People who experience transient ischaemic attack (TIA) and minor stroke have limited follow-up despite rapid specialist review in hospital. This means they often have unmet needs and feel abandoned following discharge. Care needs after TIA/minor stroke include information provision (diagnosis and stroke risk), stroke prevention (medication and lifestyle change), and holistic care (residual problems and return to work or usual activities). This protocol describes a feasibility study and process evaluation of an intervention to support people after TIA/minor stroke. The study aims to assess the feasibility and acceptability of (i) the intervention and (ii) the trial procedures for a future randomised controlled trial of this intervention.

Methods and analysis

This is a multicentre, randomised (1:1) feasibility study with a mixed-methods process evaluation. Sixty participants will be recruited from TIA clinics or stroke wards at three hospital sites (England). Intervention arm participants will be offered a nurse or allied health professional-led follow-up appointment four weeks after TIA/minor stroke. The multifaceted intervention includes: a needs checklist, action plan, resources to support management of needs, a general practitioner letter, and training to deliver the intervention. Control arm participants will receive usual care. Follow-up will be self-completed questionnaires (12- and 24-weeks) and a clinic appointment (24-weeks). Follow-up questionnaires will measure anxiety, depression, fatigue, health related quality of life, self-efficacy and medication adherence. The clinic appointment will collect body mass index, blood pressure, cholesterol and medication. Assessment of feasibility and acceptability will include quantitative process variables (such as recruitment and questionnaire response rates), structured observations of study processes, and interviews with a sub-sample of participants and clinical staff.

Ethics and dissemination

Favourable ethical opinion was gained from the Wales Research Ethics Committee (REC) 1 (23/02/2021, REC reference: 21/WA/0036). Study results will be published in peer-reviewed journals and presented at conferences. A lay summary and dissemination strategy will be co-designed with consumers. The lay summary and journal publication will be distributed on social media.

Registration details

ISRCTN reference: ISRCTN39864003

For peer review only - http://bmjopen.bmj.com/site/about/guidelines.xhtml

Strengths and limitations of this study

- The multi-centre study will enable exploration of implementation of the intervention in the context of different sites.
- The process evaluation is underpinned by the National Institutes of Health's (NIH) Behavioural Change Consortium (BCC) treatment fidelity framework.
- Quantitative and qualitative methods will explore acceptability and how the intervention is implemented in practice.
- Participants must have the ability to converse in everyday English and read in English to participate, which may limit the generalisability of our findings.

For peer review only

Introduction

Transient ischaemic attack (TIA) and minor stroke are important risk factors for stroke. Over 46,000 people experience a first TIA or minor stroke per year in the United Kingdom(1), 240,000 in the United States(2) and 0.31 million in China(3).

National guidelines promote long-term management that focuses on stroke prevention.(4-6) However, research shows TIA and minor stroke patients feel unsupported in stroke prevention – both medication and lifestyle change – and often lack basic understanding of their diagnosis, stroke risk and preventative medication.(7) Furthermore, many people experience a wide variety of residual impairments and unmet needs after TIA or minor stroke, including anxiety, mood/ emotional impact, fatigue, cognitive impairment, physical weakness, visual impairment and impaired speech.(8-17) TIA and minor stroke have been also reported to impact on people's ability to return to work, performance at work, social activities and family relationships.(12-19) Follow-up care is variable and often inadequate with patients feeling abandoned after hospital discharge.(7)

Care needs after TIA and minor stroke include information provision (diagnosis and stroke risk); stroke prevention (medication and lifestyle change); and holistic care (residual problems and return to work or usual activities).(7) However, there is no evidence for how to best support these patients after rapid specialist review in hospital. To address this, we developed a multifaceted intervention which aims to actively identify and address unmet needs after TIA and minor stroke: SUPPORT TIA (Structured follow-Up Pathway to imProve management Of Residual impairmenTs and patients' quality of life after Transient Ischaemic Attack and minor stroke). The components of the intervention are described in this protocol. In accordance with the Medical Research Council (MRC) guidance on developing and evaluating complex interventions,(20) we will evaluate the feasibility and acceptability of (i) the intervention and (ii) the trial procedures for a future randomised controlled trial (RCT) of this intervention. In addition, we will conduct a process evaluation to evaluate intervention fidelity and contextual influences on delivery.

Methods and analysis

Study design

The study is a multicentre, individual randomised feasibility study with a mixed-methods process evaluation. The trial is registered on the ISRCTN registry (Reference: ISRCTN39864003). The study is reported in accordance with the SPIRIT checklist(21) and the design is summarised in Figure 1.

The study opened for recruitment in September 2021 with planned completion by December 2022.

Patient and public involvement

A core group of three people who have experienced TIA or minor stroke have supported this study from inception, with *ad hoc* contributions from other members of the public with TIA or minor stroke. The group supported the initial development of the research question and funding application, which were informed by their priorities and experiences. The group was involved in:

For peer review only - http://bmjopen.bmj.com/site/about/guidelines.xhtml

selection of outcome measures; development of study documents; and design of the trial, such as recruitment strategies and considering participant burden related to data collection and attending intervention appointments. The group was integral to the intervention development, in particular the website of support services and resources. The group will continue to support the delivery of the study and dissemination of findings. One member (PC) is a co-author and member of the Study Oversight Committee. Patient and public involvement activities and impact will be reported using GRIPP2.(22)

Study objectives

Trial design and methods

a) Assess feasibility and acceptability of the trial design and methods, including: number of patients meeting eligibility criteria; consent and randomisation processes; recruitment and retention rates; piloting the health economics questionnaire; and data completeness;
b) Provide data to inform the sample size for a definitive randomised controlled trial (RCT);
c) Provide data to help inform selection of the primary outcome measure for a definitive RCT, including data completeness and correlation of the outcome measures with each other.

Intervention (process evaluation)

d) Investigate acceptability of the intervention for participants and intervention providers;
e) Test hypotheses relating to the theoretical underpinning of the intervention;
f) Assess if intervention providers are adequately trained to deliver the intervention;
g) Assess adherence to the intervention;
h) Assess contamination with the control group;
i) Define the "dose" of the intervention (i.e. attendance, length of appointment and number of appointments);
j) Explore how well intervention participants received and understood the intervention;
k) Explore to what extent the intervention was enacted as intended by patient participants (intervention group).

Study setting and eligibility criteria

Participants will be recruited from TIA clinics and stroke wards at three tertiary hospital sites in England, one in South East England (Berkshire) and two in North West England (Wigan and Liverpool). Participants will be adults who have experienced a first or recurrent TIA or minor stroke. The full eligibility criteria are detailed in Box 1.

Box 1: Eligibility criteria

Inclusion criteria

- Adults (aged ≥18 years);
- Resident in England;
- Diagnosis of confirmed TIA or minor stroke by a stroke consultant. TIA will be defined as a transient episode of neurological dysfunction caused by focal brain, spinal cord, or retinal ischemia, without acute infarction. Minor stroke will be defined as a modified Rankin scale score ≤1 or no change in modified Rankin scale score from pre-event (to account for people who were disabled prior to their TIA or minor stroke);

For peer review only - http://bmjopen.bmj.com/site/about/guidelines.xhtml

- Attending the TIA clinic or stroke ward for a new diagnosis of TIA or minor stroke, rather than for a follow-up appointment;
- Ability to converse in everyday English and read in English;
- Capacity to provide fully informed consent for participation in the trial.

Exclusion criteria

- History of full stroke (modified Rankin scale score >1);
- History of dementia;
- People who lack capacity to participate, such as if they have severe memory problems that mean they would not remember giving consent or if they have severe communication problems, not precluding patients who use electronic devices to communicate;
- Patients receiving early supported discharge or cardiac rehabilitation;
- Patients receiving any palliative care.

Intervention

Intervention development was underpinned by the Behaviour Change Wheel theoretical framework(23) and iteratively refined in collaboration with patient partners and a multidisciplinary team (Appendix 3, eTable 2)

The multifaceted intervention broadly comprises six components (Figure 2):

1. Training for nurses and allied health professionals (AHPs) delivering the intervention;
2. Structured nurse or AHP led follow-up appointment, 4 weeks after TIA or minor stroke;
3. Needs checklist completed by participants prior to the appointment;
4. Resources to support management of needs, including a website of resources and support services; list of local support services; and a self-management booklet;
5. Action plan;
6. Structured letter to GPs to improve the interface communication between secondary and primary care.

Participants will also receive usual care and a Stroke Association TIA information sheet. Follow-up for TIA and minor stroke is not standardised; therefore, usual care varies between hospitals, GP practices and individual clinicians. Typically, any secondary care follow-up is related to imaging and investigations to determine cause of the TIA/ minor stroke and inform stroke risk prediction; for example, carotid imaging or electrocardiogram. Follow-up in primary care usually focuses on secondary prevention, such as medication and lifestyle advice; however, presence and quality of primary care follow-up post-TIA/ minor stroke is variable.

Details of the intervention are described below in accordance with the template for intervention description and replication (TIDieR) checklist.(24) The logic model is depicted in Figure 3.

Figure 3: Logic model

For peer review only - http://bmjopen.bmj.com/site/about/guidelines.xhtml

Materials and procedures

Participants randomised to receive the intervention will be invited to a nurse/AHP-led follow-up appointment. Prior to their appointment, participants will be asked to complete a needs checklist, which will be posted to them prior to the appointment. The checklist comprises 12 potential needs which encompass information provision (diagnosis and stroke risk); secondary stroke prevention (medication and lifestyle change); and holistic care (psychological and psychosocial) (online supplementary appendix 1). The checklist is an adapted version of the Stroke Review Checklist(25) and was informed by the literature and earlier qualitative research,(7) co-designed with consumers.

The nurse/AHP will use the checklist to guide discussions to identify participants' unmet needs. If multiple needs are identified, priority will be given to addressing needs which the participant considers the most significant.

The nurse/AHP will address needs that can be resolved during the appointment, such as information about driving. For needs that cannot be immediately addressed, the nurse/AHP will, where appropriate, refer or signpost to support services and develop an action plan which will be agreed with the participant. Where possible, the nurse/AHP will make referrals; however, in some circumstances GP referral may be required, in which case this will be requested in the GP letter. To facilitate this, the intervention provider will be provided with a website of resources and support services and a list of local services.

The nurse/ AHP will take the participants' blood pressure and, if raised (≥140/90 mmHg), request for the participant's GP to review blood pressure in the action plan and GP letter.

If necessary, the nurse/AHP may invite the participant to attend another follow-up appointment, at a suitable time point, to monitor the participant's progress and revise the action plan if required. These additional follow-ups may be conducted by telephone, video call or face-to-face.

A letter will be sent to the participant's GP along with a copy of the agreed action plan. A letter template will include recommended GP actions, a summary of the appointment and actions taken.

The participant will be provided with a self-management booklet (an abridged version of the resources and services website) and a copy of the action plan and GP letter.

Intervention provider

The intervention will be delivered by a nurse or AHP, with stroke expertise, who are clinical staff at the participating hospital sites. It is anticipated that 1-2 intervention providers will be trained per site; however, this will depend on availability of clinical staff at sties. The nurses and AHPs will attend training which will include education about potential needs after TIA and minor stroke, and how to deliver the intervention. One training session, approximately 2.5 hours) will be provided remotely (via Zoom); however, ad hoc support and feedback will be encouraged after the training.

Setting and modes of delivery

The intervention appointments will be delivered at the site's TIA clinic, either face-to-face or remotely (e.g. telephone or video call). Face-to-face delivery will be preferable where possible.

When and how often

The intervention appointment will take place at four weeks (or up to six weeks). The appointment is expected to last approximately 30 minutes. One appointment will be offered initially; however, participants will have an option to attend additional follow-up if judged clinically necessary by the

For peer review only - http://bmjopen.bmj.com/site/about/guidelines.xhtml

nurse or AHP. There are no pre-determined criteria for further follow-up and the criteria used by nurses/AHPs will be recorded as part of the feasibility study to inform future refinement of the intervention.

Control arm

The control group will receive usual care and be given a Stroke Association TIA information sheet when they are informed about their allocation to the control arm.

Recruitment

A member of the clinical team will screen patients' medical records and approach potentially eligible patients face-to-face or by phone. After confirming eligibility, potential participants will be invited to take part in the study. Informed consent may be taken face-to-face (for people approached in clinic), by post (for people who need more time to consider participation) or verbally (for people approached via phone). Verbal consent will be clearly documented in the participant's medical records and the participant will also be sent a postal consent form to compete. Sites will receive a per-participant reimbursement for recruitment.

Sample size

The study will aim to recruit 60 participants (30 in the intervention group, 30 in the control group). As this is a feasibility study, no formal sample size calculation has been performed; however, the sample size is the estimated number that would be feasible to show that we can recruit these types of patients for this type of study.(26)

Randomisation

Participants will be randomised 1:1 to either the intervention or control group. A minimisation algorithm will be used within an online randomisation system to ensure balance in the treatment allocation using the following variables: age at consent (<60 years, ≥60 years); sex (male, female); diagnosis (TIA, minor stroke); employment (employed, non-employed/retired).

A 'random element' will be included in the minimisation algorithm, so that each patient has a probability, of being randomised to the opposite treatment that they would have otherwise received.

Participants will be randomised at baseline by clinical staff; however, to prevent baseline patient reported outcomes being affected by study arm allocation, participants will be notified of their randomisation allocation after they have returned the 1-week questionnaire or at 3 weeks (if the 1-week questionnaire is not returned).

Outcomes and data collection

Table 1 summarises the patient reported, health economic and clinical outcome measures. Contact details, demographic information and medical history will be collected at baseline from medical records or participant interview, by a member of clinical staff. Questionnaires comprising Patient Reported Outcome Measures (PROMs) (Table 2) will be completed by participants, either by post or electronically, at 1, 12 and 24 weeks. PROM rationale for assessment and psychometric properties are presented in eTable 1, see appendix 2. Questionnaires at 12 and 24 weeks will also collect health economics data. The first PROM completion will be at one week rather than baseline due to the nature of the PROM questions and to reduce burden on participants. Clinical data (Table 2) will be

For peer review only - http://bmjopen.bmj.com/site/about/guidelines.xhtml

collected at an end of study clinic appointment at 26 weeks by a research nurse or clinical staff. Where possible, this appointment will be face-to-face in the TIA clinic; however, may be delivered remotely if face-to-face is not an option.

Table 1: summary of patient reported, health economic and clinical outcome measures.

	Data	Timepoint
**Baseline data**	Contact details<br>Demographic: date of birth, sex, ethnicity, employment status<br>Medical: diagnosis, date of TIA or minor stroke, modified Rankin scale score, length of stay, smoking status, alcohol consumption, height, weight, body mass index, comorbidities, medication, blood pressure, cholesterol	Baseline
**Patient reported outcome measure**	Health related quality of life: Patient-Reported Outcomes Measurement Information System (PROMIS)-Global Health 10<br>Health related quality of life: 5-level EuroQol 5-Dimensions (EQ-5D-5L)<br>Anxiety/ depression: Hospital Anxiety and Depression Scale (HADS)<br>Fatigue: Fatigue Assessment Scale (FAS)<br>Self-efficacy: Patient Activation Measure-13 (PAM-13)<br>Medication adherence: Medication Adherence Rating Scale -5 (MARS)<br>Satisfaction with overall care after TIA/minor stroke question: 5-point Likert scale (very satisfied – very dissatisfied)	1, 12 and 24 weeks
**Health economics**	Use of healthcare services<br>Change in employment status, altered work hours and days off sick<br>Other costs incurred because of TIA or minor stroke	12 and 24 weeks
**Clinical data**	Body Mass Index<br>Blood pressure<br>Bloods: cholesterol<br>Medications	Baseline and 26 weeks

Feasibility outcomes and process evaluation

The feasibility study and process evaluation outcomes are detailed in Tables 2 and 3. The process evaluation is underpinned by the National Institutes of Health's (NIH) Behavioural Change Consortium (BCC) treatment fidelity framework.(27) This framework includes five domains of treatment fidelity: Study Design, Training, Delivery, Receipt and Enactment.

Table 2: Feasibility outcomes and measurement of outcomes

Objective	Feasibility outcomes	Measurement of outcome

For peer review only - http://bmjopen.bmj.com/site/about/guidelines.xhtml

a) Assess feasibility and acceptability of the trial design and methods	Number of eligible/ineligible patients and reasons for ineligibility	Recruitment log
	Proportion of participants who consent face-to-face, verbal or postal	Registration log: method of consent
	Willingness of clinical staff to randomise patients	Interviews (clinical staff involved in randomisation)
	Recruitment and attrition rates	Registration log
	Response rates and frequencies of missing data: participant completed questionnaires and case report forms	1, 12 and 24 week questionnaires<br>Case Report forms
	End of study clinic appointment attendance rates	End of Study Clinic Appointment Form
	Acceptability of the trial design	Interviews (participants and clinical staff)<br>Structured observations
b) Provide data to inform the sample size for a definitive randomised controlled trial	Standard deviations of continuous patient reported outcome measures at six months	Patient reported outcome measure scores
	Recruitment and attrition rates	Registration log
c) Provide data to help inform selection of the primary outcome measure for a definitive randomised controlled trial	Correlation of patient reported outcome measures	Patient reported outcome measure scores
	Patient reported outcome measure response rates and missing data	1, 12 and 24 week questionnaires

1

2 **Table 3: Process evaluations outcomes and measurement of outcomes**

NIH BCC domain	Objective	Outcome	Measurement of outcome
**Study design**	d) Investigate acceptability of the intervention for participants and intervention providers	Participants' and intervention providers' opinion on acceptability of the intervention	Interviews (participants and intervention providers)<br>Feedback questionnaire (intervention participants)
	e) Test hypotheses relating to the theoretical underpinning of the intervention	Participants' satisfaction with identification and management of needs	Interviews (participants and intervention providers)<br>Feedback questionnaire (intervention participants)
		Participants acting on agreed action plans and/or accessing support services	Interviews (participants)
**Training**	f) Assess if intervention providers are adequately	Intervention providers' understanding of the intervention components	Interviews (intervention providers)

For peer review only - http://bmjopen.bmj.com/site/about/guidelines.xhtml

For peer review only

	trained to deliver the intervention		
**Delivery**	g) Assess adherence to the intervention	Intervention providers' adherence to and deviations from the intervention manual	Structured observations<br>Intervention log
	h) Assess contamination with the control group	Control group contamination	Interviews (participants and clinical staff)<br>Structured observations
	i) Define the "dose" of the intervention	Intervention follow-up appointment: attendance, length of appointment and number of appointments	Intervention log
**Receipt**	j) Explore how well intervention participants received and understood the intervention	Participants' perception of the intervention	Interviews (participants)<br>Feedback questionnaire (intervention participants)
**Enactment**	k) Explore to what extent the intervention was enacted as intended by intervention participants	Participants acting on agreed action plans and/or accessing support services	Interviews (intervention participants)

Case report forms

The following case report forms will collect data on feasibility outcomes: recruitment log (recruitment rates and reasons for ineligibility); registration log (method of consent: face-to-face/ verbal/ postal); intervention log (attendance rates, duration, number of appointments per participant); end of study clinic appointment form (attendance). Case report forms will be assessed for missing data. The following intervention documents will capture information on needs, what was discussed and action plans: checklist, action plan and GP letter.

Participant completed questionnaires

Participant completed questionnaires (1, 12 and 24 weeks) will be analysed for response rates and missing data. Standard deviations of continuous PROMs at six months and correlation of PROMs will inform the sample size and selection of outcome measures for the definitive RCT. The intervention feedback questionnaire will report acceptability of the intervention. A paper copy of the feedback questionnaire and pre-paid envelope will be posted to participants after the intervention appointment. This questionnaire contains 5-point Likert scale questions (e.g. strongly agree – strongly disagree) and free text questions about experiences of the checklist, appointment and action plan.

Structured observations

A member of the study team will observe the following study processes: recruitment and consent procedures; intervention appointments; and end of study clinic appointments. Both face-to-face and remote modes of delivery will be observed for these procedures if possible. A target of three observations will be conducted for recruitment/ consent and end of study clinic appointments (one

For peer review only - http://bmjopen.bmj.com/site/about/guidelines.xhtml

at each site). A target of two intervention appointments will be observed per site (20%). More observations may be conducted if deemed necessary; for example, multiple clinical staff performing each procedure. A pragmatic approach will be taken to select which sessions to observe based on the availability of the research and clinical teams. A checklist will be used to document adherence to the protocol and field notes will be collected.

Audit

At the end of the recruitment period, each site will perform an audit to identify the total number of confirmed TIA and minor stroke patients who attended the TIA clinic or stroke ward during the recruitment period. The age and sex of these patients will also be collected. This data will be used to compare average age and sex of patients recruited to the trial against patients not recruited.

Qualitative interviews

At the end of the study, semi-structured interviews will be conducted with a subset of participants and clinical staff involved in recruitment and/or intervention delivery. The sample size is anticipated to be 8-10 patients and 4-6 clinical staff (including those involved in recruitment/consent, intervention delivery and end of study clinic appointments). For patient participants, convenience sampling will be used initially; however, sampling will become increasingly purposeful to achieve variation in age (<60 years, ≥60 years) and diagnosis (TIA, minor stroke). For clinical staff, convenience sampling will be used. Interviews will be conducted by GT, an experienced qualitative researcher. Interviews will be face-to-face (home/ hospital), telephone or video call, depending on the participants preference. Interviews will explore acceptability of the intervention and trial design. Semi-structured topic guides will include discussion of the following:

- Patient participants:
    - Intervention: intervention and trial design acceptability; how well intervention participants received and understood the intervention; extent to which intervention providers addressed needs; if the action plan was actioned; facilitators and barriers to enactment.
    - Control: trial design acceptability; intervention contamination.
    - Both: what care/support participants received; understanding what comprised usual care.
- Staff participants: acceptability of the trial design; experience of training day and understanding of the intervention; acceptability of delivering the intervention; facilitators and barriers to implementing both the trial design and the intervention; and experience of contamination with the control group.

Monitoring, adverse events and study oversight

Information on trial monitoring, adverse events and study oversight is presented in Appendix 4.

Analysis

Quantitative outcomes will be analysed using simple descriptive statistics (e.g. proportions and percentages, mean and standard deviations) and where appropriate, point estimates of effect sizes (e.g. mean differences and relative risks) and associated 95% confidence intervals. Analyses comparing the intervention and control groups will use the intention to treat principle, i.e. all participants will be analysed in the treatment group to which they were randomised irrespective of compliance or other protocol deviation. Analysis will be conducted using Stata v16.

For peer review only - http://bmjopen.bmj.com/site/about/guidelines.xhtml

For qualitative data, interviews will be audio recorded and transcribed verbatim. NVivo v12 will be used to manage, sort, code and organise the anonymised transcribed data. Interview transcripts will be analysed by GT using directed thematic analysis, using Braun and Clarke's 6-stage process,(28) informed by the research aims.(29)

The health economics analysis will assess completion rates, estimate resources required to deliver the intervention and report simple descriptive statistics for resource use and outcomes. Key resource use items not currently specified on the form but included by participants will also be identified. The information will inform the cost and outcome data collection and identification of unit costs for a larger trial.

Data will be made available upon reasonable request.

Progression criteria

The pre-defined progression criteria, detailed in Table 4, will be used to inform a decision on whether a full RCT is warranted and feasible. The criteria were agreed by the Study Oversight Committee and follow a traffic light system using quantitative measures supported by qualitative data.

Table 4: Progression criteria

Key uncertainties	Measures used	Progression criteria
**Trial design**		
Recruitment	% target sample size recruited	● ≥90%: proceed to a full-scale trial<br>● 70-89%: SOC will consider the feasibility of proceeding to a full-scale trial bearing in mind the data presented, representativeness of the sample, and possible steps to increase recruitment<br>● <70%: full-scale trial unlikely to be feasible
Randomisation*	% of consented participants randomised	● ≥90%: proceed to a full-scale trial<br>● 70-89%: SOC will consider the feasibility of proceeding to a full-scale trial bearing in mind the data presented, representativeness of the sample, and possible steps to address randomisation issues<br>● <70%: full-scale trial unlikely to be feasible
Return rate of 24-week questionnaire*	% of 24-week questionnaires returned	● ≥80%: proceed to a full-scale trial<br>● 50-79%: SOC will consider the feasibility of proceeding to a full-scale trial bearing in mind the data presented, representativeness of the sample, and possible steps to increase return rates<br>● <50%: full-scale trial unlikely to be feasible
**Intervention**		
Attendance	% of intervention arm	● ≥90%: proceed to a full-scale trial

For peer review only - http://bmjopen.bmj.com/site/about/guidelines.xhtml

rate*	participants attending first appointment	● 70-89%: SOC will consider the feasibility of proceeding to a full-scale trial bearing in mind the data presented, representativeness of the sample, and possible steps to increase attendance<br>● <70%: full-scale trial unlikely to be feasible
Delivery of the intervention	% completion of: checklists, action plans, GP letters; use of directory of support services; Issues regarding delivery of the intervention components and contamination explored in qualitative interviews	The SOC will consider the quantitative and qualitative data and make an overall judgement on whether the intervention content is delivered as intended
Acceptability	% of participants reporting acceptability of intervention components on intervention feedback questionnaire; Issues regarding acceptability of the intervention components explored in qualitative interviews	The SOC will consider the quantitative and qualitative data and make an overall judgement on whether the intervention is acceptable

* critical progression criteria: the trial is unlikely to be feasible if these criteria are not met, even if other criteria are satisfactory.
GP: General Practitioner; SOC: Study Oversite Committee; %: percent

For peer review only

Ethics and dissemination

Favourable ethical opinion was gained from the Wales Research Ethics Committee (REC) 1 (23/02/2021, REC reference: 21/WA/0036). Study results will be published in a peer-reviewed journal and presented at relevant conferences. A lay summary and dissemination strategy will be co-designed with consumers. The lay summary and peer review publication will be distributed on social media.

Authors' contributions

GT, MC, JM, RF and LA contributed to the conception of the study. The design of the feasibility study was led by GT with contributions from MC, JM, RF, LA, PC and SP, SJ and ST. The design of the intervention was led by GT with contributions from MC, JM, RF, LA, PC and RJ. GT wrote the first draft of the manuscript. All authors contributed to and approved the final version.

Acknowledgments

We thank the patient and public involvement group members who have provided valuable insight and feedback into the intervention and study design: Phil Collis, Lesley Thomson and Sally Hughes. We thank those involved in the development of the intervention with special mention to Kirsty Price and Joanne Chesson.

For peer review only - http://bmjopen.bmj.com/site/about/guidelines.xhtml

Funding statement

This work was supported by National Institute for Health Research (NIHR) Post-Doctoral Fellowship Scheme grant number PDF-2017-10-047. The views expressed are those of the author(s) and not necessarily those of the NIHR or the Department of Health and Social Care.

Competing interests

MC and JM are National Institute for Health Research (NIHR) Senior Investigators. MC also receives funding from the National Institute for Health Research (NIHR), UK Research and Innovation (UKRI), the NIHR Birmingham Biomedical Research Centre, NIHR Surgical Reconstruction and Microbiology Research Centre and NIHR ARC West Midlands at the University of Birmingham and University Hospitals Birmingham NHS Foundation Trust, UK SPINR (UKRI) Health Data Research UK, Innovate UK (part of UKRI), Macmillan Cancer Support, UCB Pharma, GSK, Gilead and Janssen. MC has received personal fees from Astellas, Aparito Ltd, CIA Oncology, Takeda, Merck, Daiichi Sankyo, Glaukos, GSK and the Patient-Centered Outcomes Research Institute (PCORI) outside the submitted work. The views expressed in this publication are those of the author(s) and not necessarily those of the NIHR or the Department of Health and Social Care. JM has done consultancy work for BMS/Pfizer, Omron, and Doctorlink Innovations Ltd. GMT, RJ, PC, SP, SJ, ST, RF and LA declare no conflict of interest.

References

1. Scarborough P, Peto V, Bhatnagar P, Kaur A, Leal J, Luengo-Fernandez R, et al. Stroke statistics. British Heart Foundation and Stroke Association: London; 2009.
2. American Stroke Association. Let's talk about Transient Ischemic Attack (TIA) [Available from: https://www.stroke.org/-/media/Stroke-Files/Lets-Talk-About-Stroke/Type-of-Stroke/LTAS_TransientIschemicAttack_2019.pdf.
3. Jiang B, Sun H, Ru X, Sun D, Chen Z, Liu H, et al. Prevalence, Incidence, Prognosis, Early Stroke Risk, and Stroke-Related Prognostic Factors of Definite or Probable Transient Ischemic Attacks in China, 2013. Front Neurol. 2017;8:309.
4. European Stroke Organisation (ESO) Executive Committee. Guidelines for management of ischaemic stroke and transient ischaemic attack 2008. Cerebrovascular diseases (Basel, Switzerland). 2008;25(5):457-507.
5. Intercollegiate Stroke Working Party. National clinical guideline for stroke. London: Royal College of Physicians; 2016.
6. Kernan WN, Ovbiagele B, Black HR, Bravata DM, Chimowitz MI, Ezekowitz MD, et al. Guidelines for the Prevention of Stroke in Patients With Stroke and Transient Ischemic Attack. Stroke. 2014;45(7):2160-236.
7. Turner GM, McMullan C, Atkins L, Foy R, Mant J, Calvert M. TIA and minor stroke: a qualitative study of long-term impact and experiences of follow-up care. BMC Family Practice. 2019;20(1):176.
8. Moran GM, Fletcher B, Feltham MG, Calvert M, Sackley C, Marshall T. Fatigue, psychological and cognitive impairment following transient ischaemic attack and minor stroke: a systematic review. European journal of neurology. 2014;21(10):1258-67.
9. Sackley CM, Mant J, McManus RJ, Humphreys G, Sharp L, Mares K, et al. Functional and emotional outcomes after transient ischemic attack: A 12-month prospective controlled cohort study. International journal of stroke. 2019:1747493018823158.

For peer review only - http://bmjopen.bmj.com/site/about/guidelines.xhtml

10. Turner GM, Calvert M, Feltham MG, Ryan R, Marshall T. Ongoing impairments following transient ischaemic attack: retrospective cohort study. European journal of neurology. 2016;23(11):1642-50.
11. van Rooij FG, Kessels RP, Richard E, De Leeuw FE, van Dijk EJ. Cognitive Impairment in Transient Ischemic Attack Patients: A Systematic Review. Cerebrovascular diseases (Basel, Switzerland). 2016;42(1-2):1-9.
12. Gibson J, Watkins C. People's experiences of the impact of transient ischaemic attack and its consequences: qualitative study. Journal of advanced nursing. 2012;68(8):1707-15.
13. Green TL, King KM. Experiences of male patients and wife-caregivers in the first year post-discharge following minor stroke: a descriptive qualitative study. International journal of nursing studies. 2009;46(9):1194-200.
14. Kamara S, Singh S. What are the patient-held illness beliefs after a transient ischaemic attack, and do they determine secondary prevention activities: an exploratory study in a North London General Practice. Primary health care research & development. 2012;13(2):165-74.
15. Rochette A, Desrosiers J, Bravo G, St-Cyr-Tribble D, Bourget A. Changes in participation after a mild stroke: quantitative and qualitative perspectives. Topics in stroke rehabilitation. 2007;14(3):59-68.
16. Spurgeon L, James G, Sackley C. Subjective experiences of transient ischaemic attack: a repertory grid approach. Disability and rehabilitation. 2013;35(26):2205-12.
17. Taule T, Raheim M. Life changed existentially: a qualitative study of experiences at 6-8 months after mild stroke. Disability and rehabilitation. 2014;36(25):2107-19.
18. Carlsson GE, Moller A, Blomstrand C. Managing an everyday life of uncertainty--a qualitative study of coping in persons with mild stroke. Disability and rehabilitation. 2009;31(10):773-82.
19. Croot EJ, Ryan TW, Read J, Campbell F, O'Cathain A, Venables G. Transient ischaemic attack: a qualitative study of the long term consequences for patients. BMC Fam Pract. 2014;15:174.
20. Skivington K, Matthews L, Simpson SA, Craig P, Baird J, Blazeby JM, et al. A new framework for developing and evaluating complex interventions: update of Medical Research Council guidance. BMJ. 2021;374:n2061.
21. Chan A-W TJ, Altman DG, Laupacis A, Gøtzsche PC, Krleža-Jerić K, Hróbjartsson A, Mann H, Dickersin K, Berlin J, Doré C, Parulekar W, Summerskill W, Groves T, Schulz K, Sox H, Rockhold FW, Rennie D, Moher D. SPIRIT 2013 Statement: Defining Standard Protocol Items for Clinical Trials. Annals of Internal Medicine. 2013;158(3):200-7.
22. Staniszewska S, Brett J, Simera I, Seers K, Mockford C, Goodlad S, et al. GRIPP2 reporting checklists: tools to improve reporting of patient and public involvement in research. BMJ. 2017;358:j3453.
23. Michie S, van Stralen MM, West R. The behaviour change wheel: A new method for characterising and designing behaviour change interventions. Implementation Science. 2011;6(1):42.
24. Hoffmann TC, Glasziou PP, Boutron I, Milne R, Perera R, Moher D, et al. Better reporting of interventions: template for intervention description and replication (TIDieR) checklist and guide. BMJ : British Medical Journal. 2014;348:g1687.
25. Turner GM, Mullis R, Lim L, Kreit L, Mant J. Using a checklist to facilitate management of long-term care needs after stroke: insights from focus groups and a feasibility study. BMC Family Practice. 2019;20(1):2.
26. Teare MD, Dimairo M, Shephard N, Hayman A, Whitehead A, Walters SJ. Sample size requirements to estimate key design parameters from external pilot randomised controlled trials: a simulation study. Trials. 2014;15(1):264.
27. Bellg AJ, Borrelli B, Resnick B, Hecht J, Minicucci DS, Ory M, et al. Enhancing treatment fidelity in health behavior change studies: best practices and recommendations from the NIH Behavior Change Consortium. Health Psychol. 2004;23(5):443-51.
28. Braun V, Clarke V. Using thematic analysis in psychology. Qualitative Research in Psychology. 2006;3(2):77-101.

For peer review only - http://bmjopen.bmj.com/site/about/guidelines.xhtml

29. Fereday J, Muir-Cochrane E. Demonstrating Rigor Using Thematic Analysis: A Hybrid Approach of Inductive and Deductive Coding and Theme Development. International Journal of Qualitative Methods. 2006;5(1):80-92.

Figure legends

Figure 1: Trial schema

Figure 2: Summary of the intervention components (AHP: Allied Health Professional; GP: General Practitioner)

Figure 3: Logic model

For peer review only

For peer review only - http://bmjopen.bmj.com/site/about/guidelines.xhtml

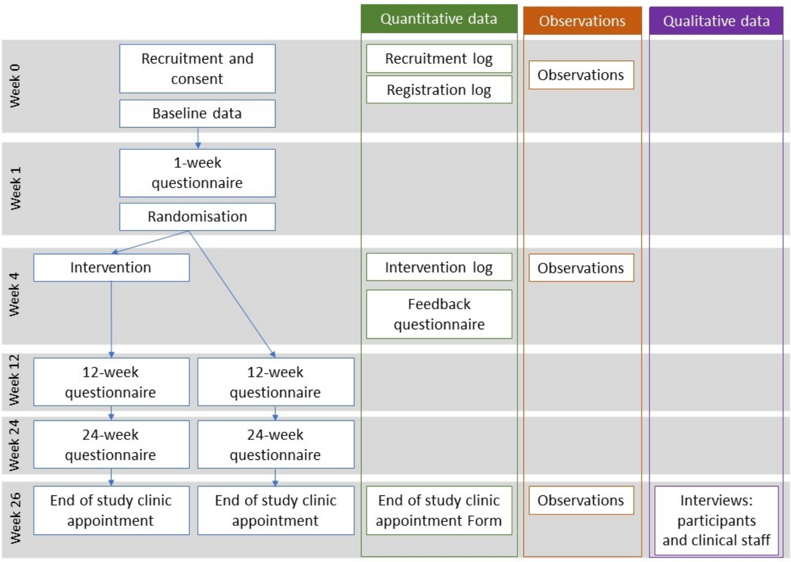

Figure 1

338x190mm (96 x 96 DPI)

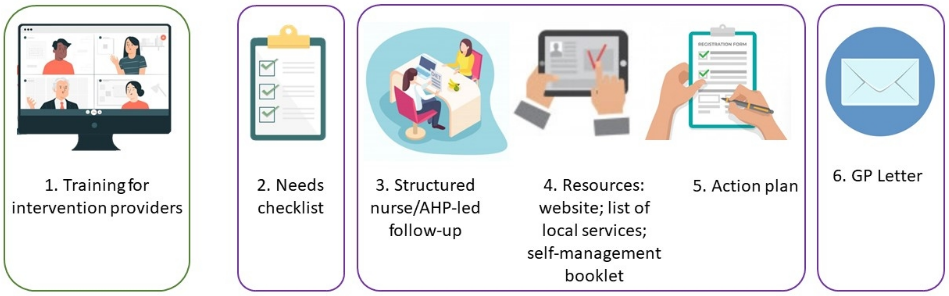

This figure has been designed using resources from Freepik.com

Figure 2

338x190mm (96 x 96 DPI)

Intervention component	Mechanism of change	Outputs	Potential outcomes
**1. Training for nurses or AHPs delivering the intervention.**	HCPs educated about potential needs after TIA or minor stroke, including health and social consequences.<br>HCPs instructed how to identify and address potential unmet needs, including use of intervention materials.	↑ HCPs knowledge of potential needs and strategies to identify and address needs.	***Short term***<br>• ↑ satisfaction with care.<br>• Access relevant further support (e.g. GP, support service).<br>• Access relevant resources (e.g. websites, apps).<br>• ↑ knowledge and understanding of diagnosis and stroke risk.<br><br>***Medium term***<br>• ↑ medication adherence.<br>• ↑ confidence and ability to self-manage needs.<br><br>***Long term***<br>• ↑ health related quality of life.<br>• ↓ or improved residual problems (e.g. anxiety, fatigue).<br>• ↓ in stroke risk factors.
**2. Structured nurse or AHP led follow-up appointment, 4 weeks after TIA or minor stroke.**	Service to provide patients with access to nurse or AHP follow-up.	Patients access holistic care and support for needs related to their TIA or minor stroke. Needs may be actioned immediately (e.g. driving information, reassurance) or an agreed plan for self-management or further support.	
**3. Needs checklist completed by participants prior to the appointment.**	Checklist provides patients with the opportunity to reflect on their needs and facilitates communication with the HCP.	Patients' needs are actively identified and acknowledged by the HCP.	
**4. Resources to support management of needs, including a website of resources and support services; list of local support services; and a self-management booklet.**	↑ knowledge of relevant support services and resources.	Patients are referred or signposted to relevant support services.<br>Patients are recommended relevant resources for their individual needs (e.g. websites, apps).	
**5. Action plan.**	Provides an opportunity for shared decision making and goal setting, and empowers patient to access services or resources and/or self-manage.	Patients are given clear actions to self-manage needs or access further support.	
**6. Structured GP letter**	↑ communication between secondary and primary care, and patients and GPs.<br>Patients are empowered to access GP support.	GPs have better understanding of their patients' needs and care received. GPs receive clear recommendations for how to further support patient needs.	

AHP: Allied Health Professional; GP: General Practitioner; HCP: Healthcare provider; TIA: Transient Ischaemic Attack; ↑: increase; ↓: decrease

Figure 3

233x160mm (96 x 96 DPI)

TNO

My **information** and **support needs** after mini-stroke

This is a list of needs that some people have after mini stroke.

Please tick (✓) any areas you have difficulty with, even if you have only experienced it occasionally.

Your follow-up appointment is an opportunity to discuss any worries or problems you might be experiencing, and any advice or information you may need following your mini stroke (TIA) or minor stroke. Please use this list to help you to prepare for your appointment.

For peer review only

Information

1. I need more information about:	*Tick here*
• My diagnosis	
• My risk of stroke	
• Driving after my mini stroke	

Stroke prevention

2. I need advice on:	*Tick here*
• Medications for preventing another stroke	
• Medication side effects	
• Lifestyle change, such as exercise or diet, to prevent another stroke	

Effects of mini-stroke

3. Fatigue	*Tick here*
• I feel tired most of the time or I get easily tired	
• I find it difficult to concentrate and do things	
**4. Mood**	
• I feel anxious	
• I feel depressed	
• I experience anger, frustration or mood swings	
• I feel that my personality has changed	
**5. Memory and thinking**	
• I find it difficult to think, concentrate, or remember things	

For peer review only - http://bmjopen.bmj.com/site/about/guidelines.xhtml

TNO | | | | |

**6. Communication**	
• I finding it difficult to understand / communicate with others	
• I have problems with speech, word finding or talking to others	
**7. Physical**	
• I experience muscle weakness or problems with balance	
• I have headaches	
• I am sensitive to noise or light	
**8. Incontinence**	
• I am having a problem controlling my bladder or bowels	
**9. Intimate relationships**	
• Since my mini stroke I have problems with sex	
**10. Work or education**	
• I am having problems at work or education	
• I would like support and advice on returning to work or education	
**11. Relationships with family or friends**	
• My personal relationships with my family or friends have become difficult or stressed	
**12. Social activities or daily tasks**	
• I find it difficult to take part in hobbies or leisure activities	
• I have difficulty doing daily tasks	

**Which of the needs you identified above is your biggest concern?**

**Is there anything else you are concerned about or would like more information about?**

Please bring this list to your follow-up appointment

For peer review only - http://bmjopen.bmj.com/site/about/guidelines.xhtml

eTable 1: Summary of proposed Patient Reported Outcome Measures (PROMs) for the main Randomised Controlled Trial.

Domain	PROM	Summary	Rationale
Health related quality of life	PROMIS-Global Health 10	**Questions:** 10<br>**Scoring:** 5-point Likert + 10 point Likert pain question. Scored using item-level calibrations- use the Health Measures Scoring Service<br>**MID:** none specified<br>**Permissions:** publicly available for use	Recommended by the International Consortium for Health Outcomes Measurement (2016) as part of a consensus stroke measure standard set
Health related quality of life	EQ-5D-5L	**Questions:** 5 + 1 VAS (20 cm)<br>**Scoring:** 5-point Likert scale and VAS. Score = 0-1<br>**MID:** for stroke: 0.08 to 0.12<br>**Permissions:** licence agreement needed (€600)	Used to calculate Quality Adjusted Life Years for health economics analyses
Anxiety/ depression	HADS	**Questions:** 14<br>**Scoring:** 4-point Likert Scale. 0-7 = Normal; 8-10 = Borderline abnormal; 11-21 = Abnormal<br>**MID:** none specified<br>**Permissions:** license agreement needed (0-1000 participants= £0.85 each)	Widely used in TIA/ minor stroke research, comprehensive psychometric evaluation
Fatigue	FAS	**Questions:** 10<br>**Scoring:** 5-point Likert Scale; Score = 10 (lowest fatigue) to 50 (highest fatigue). 10-21: no fatigue (normal), 22-50: substantial fatigue<br>**MID:** at least 4 points or 10% change of the baseline value<br>**Permissions:** must acknowledge the ild care foundation in the manuscript, FAS needs to be added as a keyword in the final publication, and PDF of the final publication must be send to the ild care foundation: info@ildcare.nl	Systematic review of fatigue scales for stroke scales identified the FAS had the best test-retest reliability
Self-efficacy	PAM-13	**Questions**: 13<br>**Scoring:** 5-point Likert Scale; Score 1-100: 4 Levels: Level 1 ≤47.0 (not believing activation is important), Level 2 47.1–55.1 (a lack of knowledge and confidence to take action), Level 3 55.2–67.0 (beginning to take action), level 4 ≥67.1 (taking action)<br>**MID:** not specified<br>**Permissions:** licence agreement NOT needed for feasibility studies	Used by NHS England
Medication adherence	MARS-5	**Questions:** 6<br>**Scoring:** 5-point Likert scale, score= 5 to 25 (higher scores= higher self-reported adherence)<br>**MID:** not specified<br>**Permissions:** Free access (subject to adequate citation) for Academic users	Validated in stroke patients, widely used
Satisfaction with care	Satisfaction with care question	Not applicable- non-validated bespoke question	

EQ-5D-5L: 5-level EuroQol 5-Dimensions; FAS: Fatigue Assessment Scale; HADS: Hospital Anxiety and Depression Scale; MARS-5: Medication adherence: Medication Adherence Rating Scale -5; MID: Minimally Important Difference; NHS: National Health Service; PAM-13: Patient Activation Measure-13; PDF: Portable Document Format; TIA: Transient Ischaemic Attack; VAS: Visual Analogue Scale

For peer review only

Summary of intervention development, underpinned by the theoretical framework - Behaviour Change Wheel

- **Select target behaviour:** Healthcare provider identifying and addressing needs post-TIA/ minor stroke.
- **Identify what needs to change:**
  - Semi-structured interviews were conducted with healthcare providers and patients to identify influences on the target behaviour (results reported elsewhere). Transcripts were coded using the Theoretical Domains Framework (TDF).
- **Map TDF domains to "intervention functions"**
  - Four relevant intervention function identified: Education, Training, Environmental restructuring, Enablement.
- **Identify Behaviour Change Techniques (BCT):**
  - The BCT Taxonomy was used to identity appropriate BCT that mapped to relevant intervention functions.

The above process was informed by relevant literature; iterative feedback from patient partners and a multidisciplinary team (nurses, allied health professionals, GPs, consultants, researcher); and consideration of the APEASE criteria (Affordability, Practicality, Effectiveness and cost-effectiveness, Acceptability, Side-effects/safety, and Equity).

eTable 2: Summary of the barriers/ enablers mapped to Theoretical Domain Framework domains, intervention functions, Behaviour Change Taxonomies and intervention components.

Barriers (B)/ Enablers (E)	TDF	BCW intervention function	BCT	SUPPORT TIA Intervention component
HCPs' lack of knowledge of potential needs post-TIA/ minor stroke (B).<br><br>HCPs' perceived role in follow-up care influenced their approach to identifying and addressing needs (B/E).	Knowledge<br><br>Social professional role and identity<br><br>Goal	Education/ Training	*Information about health consequences*<br><br>*Information about social and environmental consequences*<br><br>*Information about emotional consequences*<br><br>*Instruction on how to perform the behaviour*<br><br>*Identity associated with changed behaviour*	Training for intervention providers

For peer review only

Lack of follow-up pathway following rapid hospital specialist review (B).<br><br>Time constraints (B).<br><br>Nurses and AHPs were more holistic in their approach to care (compared to doctors) and considered this part of their role (E).	Environmental context and resources<br><br>Social professional role and identity<br><br>Intentions	Environmental restructuring	*Restructure physical environment*	Structured nurse/AHP-led follow-up appointment
Checklists considered useful prompts to identify needs (E).		Environmental restructuring/ Enablement	*Prompt/ cues*	Needs checklist completed by participants prior to the appointment
HCPs' lack of knowledge of support services and resources to support needs (B).<br><br>Directories of support services facilitated identification of support services (if up-to-date) (E).	Knowledge<br><br>Environmental context and resources	Environmental restructuring/ Enablement	*Instruction on how to perform the behaviour*<br><br>*Adding objects to the environment*	Resources to support management of needs, including a website of resources and support services; list of local support services; and a self-management booklet
Patients feel unsupported after hospital (B).<br><br>Difficult for patients to process and retain information (B).<br><br>Patients attempted to access information and support themselves, but found it overwhelming, confusing, contradictory (B).	Social influences	Environmental restructuring/ Enablement	*Action planning*<br><br>*Goal setting*<br><br>*Problem solving*<br><br>*Pharmacological support*	Action plan
Restricted communication between primary and secondary care (B).<br><br>Variability in the speed and content of GP letters (B/E)	Environmental context and resources<br><br>Social influences	Environmental restructuring/ Enablement	*Action planning*<br><br>*Prompts/cues*	Structured GP letter

For peer review only

Protocol: additional information

Trial Steering Committee, Data Monitoring Committee and Study Oversight Committee

There will be no Trial Steering Committee or Data Monitoring Committee, the Study Oversight Committee will take the role of a joint Trial Steering Committee and Data Monitoring Committee.

The role of the independent Study Oversight Committee is to provide overall oversight of the trial, including the practical aspects of the study, as well as ensuring that the study is run in a way which is both safe for the patients and provides appropriate feasibility data to the sponsor and investigators.

The Study Oversight Committee will receive only aggregate data and will be blinded to the treatment allocation until the final analysis is presented. Members are independent of the investigators, their employing organisations, funders and sponsors. The Study Oversight Committee will report directly to the Trial Management Group.

Adverse events

The collection and reporting of Adverse Events will be in accordance with the UK Policy Framework for Health and Social Care Research and the requirements of the Health Research Authority. Definitions of different types of Adverse Events are listed in the table of abbreviations and definitions.

No risks are expected to arise from taking part in the trial. There are no Investigational Medicinal Products being used as part of the SUPPORT TIA trial. The intervention is considered low risk and consists of a nurse/AHP-led follow-up appointment which has been used in other populations and settings without evidence of harm. No Serious Adverse Events are anticipated as a unique consequence of participation in the SUPPORT TIA trial, but reporting requirements are clearly outlined in this section.

Adverse Events: There may be certain Adverse Events which are commonly expected in participants who have suffered a TIA or minor stroke. However, as these events are well characterised, it is highly unlikely that this trial will reveal any new safety information relating to this intervention. Therefore, we will not be collecting non-serious Adverse Events for this trial.

Serious Adverse Advents: Investigators should only report Serious Adverse Events which are attributable to the trial intervention. The above events are not considered related to the trial intervention and are therefore excluded from notification to the SUPPORT TIA Trial Office as Serious Adverse Events. These events should continue to be recorded in the medical records according to local practice. We are only reporting Serious Adverse Events which are attributable to the trial intervention; therefore, the control group will not be monitored.

Participant Withdrawal

Participants will be made aware at the beginning that they can freely withdraw (discontinue participation) from the trial (or part of) at any time. Types of withdrawal as defined are:

- The participant would like to withdraw from trial treatment, but is willing to be followed up in accordance with the schedule of assessments and, if applicable, using any central UK NHS bodies for long-term outcomes (i.e. the participant has agreed that data can be collected and used in the trial analysis).

For peer review only - http://bmjopen.bmj.com/site/about/guidelines.xhtml

- The participant would like to withdraw from trial treatment and does not wish to attend trial visits in accordance with the schedule of assessments but is willing to be followed up at standard clinic visits and, if applicable, using any central UK NHS bodies for long-term outcomes (i.e. the participant has agreed that data can be collected at standard clinic visits and used in the trial analysis, including data collected as part of long-term outcomes).
- The participant would like to withdraw from trial treatment and is not willing to be followed up in any way for the purposes of the trial and for no further data to be collected (i.e. only data collected prior to the withdrawal can be used in the trial analysis).

The details of withdrawal (date, reason and type of withdrawal) will be clearly documented in the study discontinuation form.

Note: participants involved in the qualitative sub-study may only withdraw from this part of the study up to the point of data analysis (five working days following the interview). After this point, it will not be possible to extract an individuals' interview data from the analyses.

Monitoring

Monitoring will be conducted as required following a risk assessment by the trials unit. Given the low-risk nature of this trial, central monitoring will be routine and no onsite monitoring is planned.

Onsite Monitoring: For this trial, no onsite monitoring is planned due to the low risk of the intervention and nature of the outcome data.

Central Monitoring: Trials staff will be in regular contact with the site research team to check on progress and address any queries that they may have. Recruitment rates, per site, will be monitored on a monthly basis. Trials staff will check incoming Informed Consent Forms and Case Report Forms for compliance with the protocol, data consistency, missing data and timing. Sites will be sent Data Clarification Forms requesting missing data or clarification of inconsistencies or discrepancies. Sites will be requested to send in copies of signed Informed Consent Forms and other documentation for in-house review for all participants providing explicit consent. Structured observations will be conducted as part of the trial of recruitment and consent procedures; intervention appointments; and end of study clinic appointments. Reports from these observations will be used to monitor protocol compliance.

Audit and Inspection: The Investigator will permit trial-related monitoring, audits, ethical review, and regulatory inspection(s) at their site, providing direct access to source data/documents. The investigator will comply with these visits and any required follow up. Sites are also requested to notify Birmingham Clinical Trials Unit (BCTU) of any relevant inspections.

Protocol amendments

If the Chief Investigator wishes to make a substantial amendment to the Research Ethics Committee (REC) application or the supporting documents, the Chief Investigator will submit a valid notice of amendment to the REC for consideration. It is the Sponsor's responsibility to decide whether an amendment is substantial or non-substantial for the purposes of submission to the REC.

Amendments will be notified to the REC and Health Research Authority (HRA), and communicated to the participating organisations (R&D office and local research team) departments of participating sites to assess whether the amendment affects the NHS permission for that site. The amendment history will be tracked to identify the most recent protocol version.

For peer review only - http://bmjopen.bmj.com/site/about/guidelines.xhtml

Confidentiality

Personal data recorded on all documents will be regarded as strictly confidential and will be handled and stored in accordance with the Data Protection Act 2018.

Participants will be identified using their unique trial identification number in correspondence between the site and BCTU. Participants will give their explicit consent for the movement of their consent form, giving permission for BCTU to be sent a copy. This will be used to perform in-house monitoring of the consent process. Participants will provide their personal contact details to the central research team at BCTU so they are able to contact the participants for follow-up questionnaires.

The Investigator must maintain documents not for submission to BCTU in strict confidence. In the case of specific issues and/or queries from the regulatory authorities, it will be necessary to have access to the complete trial records, provided that participant confidentiality is protected.

BCTU will maintain the confidentiality of all participant's data and will not disclose information by which participants may be identified to any third party, with the exception of the transcription service. A professional transcription company that already works with the University of Birmingham will transcribe the audio files. This company will be required to sign a confidentiality agreement before any files are sent to them. A member of the research team will check the transcripts once received from the transcription company and remove any names/ identifiers from the documents. Once the accuracy of the transcriptions has been confirmed, the original recordings will be deleted.

Representatives of the SUPPORT TIA trial team and sponsor may be required to have access to participant's notes for quality assurance purposes but participants should be reassured that their confidentiality will be respected at all times.

For participants involved in the qualitative aspects of the study, we will ask their permission to audio record the study interview using a digital recording device. We will then ask a reputable company to produce a written version of the recording called a transcript. The transcript company will need to sign a confidentiality agreement before they do so. We will then anonymise the transcript, removing all identifying information. After this, we will delete the original recording. We will only use anonymised quotes from the transcript in any arising publications or reports.

The research team will hold personal contact data for participants wishing to receive a summary of the results of the study - we anticipate this will be made available within 12 months of completion of the study. We will delete participants' contact details when the data is archived, meaning no personal identifiable data, other than study consent forms, will be retained.

There is potential that participants may disclose information that either indicates a risk or harm to themselves or others, evidence of malpractice or criminality. Participants will be informed in the Participant Information Sheet that if they disclose any of these issues, this will be reported to appropriate authorities.

Dissemination: Authorship eligibility guidelines and any intended use of professional writers

Publication policy: Results of this trial will be submitted for publication in a peer reviewed journal. The manuscript will be prepared by the Chief Investigator and authorship will be determined by the trial publication policy. No professional writers will be used.

For peer review only - http://bmjopen.bmj.com/site/about/guidelines.xhtml

Informed consent materials: Model consent form

I have read and understood the participant information sheet (Version 3.0, Dated 06/09/2021).	
I have had the opportunity to take time to consider my involvement in the trial and I have had the chance to ask questions, all of which have been answered to my satisfaction.	
I understand that my involvement in the trial is voluntary, and I am free to withdraw at any time without the quality of my medical care or my legal rights being affected.	
I understand that if I decide to withdraw from the trial, any information that has already been collected and anonymised may be used for analysis and publication.	
I understand a copy of this consent form and my data collected during the trial will be transferred to the central trials office at the Birmingham Clinical Trials Unit (BCTU), part of the University of Birmingham.	
I understand that all information collected will be used for medical research only and that I will not be identified in any way in the analysis and reporting of results. The personal information will include name, gender, date of birth, contact details and NHS number as well as medical information and study outcome assessments. It will be held securely and confidentially at BCTU.<br>I give permission for the transfer and storage of this data.	
I understand that relevant sections of my medical notes, information related directly to my participation in this trial, and data collected during the trial may be looked at by individuals from the University BCTU trial team, representatives of the sponsor, regulatory authorities, and the NHS Trust/Health Board where it is relevant to my taking part in this research.<br>I give permission for these individuals to have access to my records.	
I understand that my data collected during the trial may be shared with academic collaborative third parties who will help with the study's analysis. Before sharing, my personal identifiers (e.g. name, date of birth, contact details) will be removed and replaced with a trial number.	
I understand that my name and contact details will be used by the study team to contact me regarding the study. This may include, but not limited to, request additional information such as missing data on questionnaires I have completed.	
I understand that the research team have a duty to inform appropriate authorities if I disclose information that either indicates a risk or harm to myself or others, evidence of malpractice or criminality. In this circumstance confidentiality may be broken.	
I give permission to my GP being informed about my participation in the SUPPORT TIA Trial	
I voluntarily agree to take part in this study.	
Optional: I agree to be contacted to be invited for an interview about my involvement in the study and agree to my contact details being passed on to the research team at the University of Birmingham for them to contact me about this interview	
Optional: I would like to be sent a summary of the findings from the study and consent to my contact details to be held until this summary has been sent.	

Name of Participant	Signature	Date (DD/MMM/YYYY)
Name of Person taking Consent	Signature	Date (DD/MMM/YYYY)

For peer review only - http://bmjopen.bmj.com/site/about/guidelines.xhtml

For peer review only

For peer review only - http://bmjopen.bmj.com/site/about/guidelines.xhtml

SPIRIT
STANDARD PROTOCOL ITEMS: RECOMMENDATIONS FOR INTERVENTIONAL TRIALS

SPIRIT 2013 Checklist: Recommended items to address in a clinical trial protocol and related documents*

Section/item	Item No	Description	Page
**Administrative information**			
Title	1	Descriptive title identifying the study design, population, interventions, and, if applicable, trial acronym	1
Trial registration	2a	Trial identifier and registry name. If not yet registered, name of intended registry	3
	2b	All items from the World Health Organization Trial Registration Data Set	n/a
Protocol version	3	Date and version identifier	n/a
Funding	4	Sources and types of financial, material, and other support	15
Roles and responsibilities	5a	Names, affiliations, and roles of protocol contributors	1
	5b	Name and contact information for the trial sponsor	1
	5c	Role of study sponsor and funders, if any, in study design; collection, management, analysis, and interpretation of data; writing of the report; and the decision to submit the report for publication, including whether they will have ultimate authority over any of these activities	15
	5d	Composition, roles, and responsibilities of the coordinating centre, steering committee, endpoint adjudication committee, data management team, and other individuals or groups overseeing the trial, if applicable (see Item 21a for data monitoring committee)	~~REC protocol~~ Appendix
**Introduction**			
Background and rationale	6a	Description of research question and justification for undertaking the trial, including summary of relevant studies (published and unpublished) examining benefits and harms for each intervention	5
	6b	Explanation for choice of comparators	9
Objectives	7	Specific objectives or hypotheses	5

For peer review only

For peer review only - http://bmjopen.bmj.com/site/about/guidelines.xhtml

Trial design	8	Description of trial design including type of trial (eg, parallel group, crossover, factorial, single group), allocation ratio, and framework (eg, superiority, equivalence, noninferiority, exploratory)	5
**Methods: Participants, interventions, and outcomes**			
Study setting	9	Description of study settings (eg, community clinic, academic hospital) and list of countries where data will be collected. Reference to where list of study sites can be obtained	6
Eligibility criteria	10	Inclusion and exclusion criteria for participants. If applicable, eligibility criteria for study centres and individuals who will perform the interventions (eg, surgeons, psychotherapists)	6
Interventions	11a	Interventions for each group with sufficient detail to allow replication, including how and when they will be administered	7-9
	11b	Criteria for discontinuing or modifying allocated interventions for a given trial participant (eg, drug dose change in response to harms, participant request, or improving/worsening disease)	n/a
	11c	Strategies to improve adherence to intervention protocols, and any procedures for monitoring adherence (eg, drug tablet return, laboratory tests)	n/a
	11d	Relevant concomitant care and interventions that are permitted or prohibited during the trial	n/a
Outcomes	12	Primary, secondary, and other outcomes, including the specific measurement variable (eg, systolic blood pressure), analysis metric (eg, change from baseline, final value, time to event), method of aggregation (eg, median, proportion), and time point for each outcome. Explanation of the clinical relevance of chosen efficacy and harm outcomes is strongly recommended	10-12
Participant timeline	13	Time schedule of enrolment, interventions (including any run-ins and washouts), assessments, and visits for participants. A schematic diagram is highly recommended (see Figure)	5 (figure 1)
Sample size	14	Estimated number of participants needed to achieve study objectives and how it was determined, including clinical and statistical assumptions supporting any sample size calculations	6
Recruitment	15	Strategies for achieving adequate participant enrolment to reach target sample size	6
**Methods: Assignment of interventions (for controlled trials)**			
Allocation:			7

For peer review only - http://bmjopen.bmj.com/site/about/guidelines.xhtml

Sequence generation	16a	Method of generating the allocation sequence (eg, computer-generated random numbers), and list of any factors for stratification. To reduce predictability of a random sequence, details of any planned restriction (eg, blocking) should be provided in a separate document that is unavailable to those who enrol participants or assign interventions	7
Allocation concealment mechanism	16b	Mechanism of implementing the allocation sequence (eg, central telephone; sequentially numbered, opaque, sealed envelopes), describing any steps to conceal the sequence until interventions are assigned	7
Implementation	16c	Who will generate the allocation sequence, who will enrol participants, and who will assign participants to interventions	7
Blinding (masking)	17a	Who will be blinded after assignment to interventions (eg, trial participants, care providers, outcome assessors, data analysts), and how	7
	17b	If blinded, circumstances under which unblinding is permissible, and procedure for revealing a participant's allocated intervention during the trial	7
**Methods: Data collection, management, and analysis**			
Data collection methods	18a	Plans for assessment and collection of outcome, baseline, and other trial data, including any related processes to promote data quality (eg, duplicate measurements, training of assessors) and a description of study instruments (eg, questionnaires, laboratory tests) along with their reliability and validity, if known. Reference to where data collection forms can be found, if not in the protocol	10-12
	18b	Plans to promote participant retention and complete follow-up, including list of any outcome data to be collected for participants who discontinue or deviate from intervention protocols	n/a
Data management	19	Plans for data entry, coding, security, and storage, including any related processes to promote data quality (eg, double data entry; range checks for data values). Reference to where details of data management procedures can be found, if not in the protocol	
Statistical methods	20a	Statistical methods for analysing primary and secondary outcomes. Reference to where other details of the statistical analysis plan can be found, if not in the protocol	13
	20b	Methods for any additional analyses (eg, subgroup and adjusted analyses)	n/a

For peer review only - http://bmjopen.bmj.com/site/about/guidelines.xhtml

	20c	Definition of analysis population relating to protocol non-adherence (eg, as randomised analysis), and any statistical methods to handle missing data (eg, multiple imputation)	n/a
**Methods: Monitoring**			
Data monitoring	21a	Composition of data monitoring committee (DMC); summary of its role and reporting structure; statement of whether it is independent from the sponsor and competing interests; and reference to where further details about its charter can be found, if not in the protocol. Alternatively, an explanation of why a DMC is not needed	Appendix ~~REC protocol~~
	21b	Description of any interim analyses and stopping guidelines, including who will have access to these interim results and make the final decision to terminate the trial	n/a
Harms	22	Plans for collecting, assessing, reporting, and managing solicited and spontaneously reported adverse events and other unintended effects of trial interventions or trial conduct	Appendix ~~REC protocol~~
Auditing	23	Frequency and procedures for auditing trial conduct, if any, and whether the process will be independent from investigators and the sponsor	Appendix ~~REC protocol~~
**Ethics and dissemination**			
Research ethics approval	24	Plans for seeking research ethics committee/institutional review board (REC/IRB) approval	15
Protocol amendments	25	Plans for communicating important protocol modifications (eg, changes to eligibility criteria, outcomes, analyses) to relevant parties (eg, investigators, REC/IRBs, trial participants, trial registries, journals, regulators)	Appendix ~~REC protocol~~
Consent or assent	26a	Who will obtain informed consent or assent from potential trial participants or authorised surrogates, and how (see Item 32)	6
	26b	Additional consent provisions for collection and use of participant data and biological specimens in ancillary studies, if applicable	n/a
Confidentiality	27	How personal information about potential and enrolled participants will be collected, shared, and maintained in order to protect confidentiality before, during, and after the trial	Appendix ~~REC protocol~~
Declaration of interests	28	Financial and other competing interests for principal investigators for the overall trial and each study site	15
Access to data	29	Statement of who will have access to the final trial dataset, and disclosure of contractual agreements that limit such access for investigators	13

For peer review only - http://bmjopen.bmj.com/site/about/guidelines.xhtml

Ancillary and post-trial care	30	Provisions, if any, for ancillary and post-trial care, and for compensation to those who suffer harm from trial participation	n/a
Dissemination policy	31a	Plans for investigators and sponsor to communicate trial results to participants, healthcare professionals, the public, and other relevant groups (eg, via publication, reporting in results databases, or other data sharing arrangements), including any publication restrictions	15
	31b	Authorship eligibility guidelines and any intended use of professional writers	Appendix REC protocol
	31c	Plans, if any, for granting public access to the full protocol, participant-level dataset, and statistical code	n/a
**Appendices**			
Informed consent materials	32	Model consent form and other related documentation given to participants and authorised surrogates	Appendix REC protocol
Biological specimens	33	Plans for collection, laboratory evaluation, and storage of biological specimens for genetic or molecular analysis in the current trial and for future use in ancillary studies, if applicable	n/a

*It is strongly recommended that this checklist be read in conjunction with the SPIRIT 2013 Explanation & Elaboration for important clarification on the items. Amendments to the protocol should be tracked and dated. The SPIRIT checklist is copyrighted by the SPIRIT Group under the Creative Commons "Attribution-NonCommercial-NoDerivs 3.0 Unported" license.

For peer review only

BMJ Open

Structured follow-up pathway to support people after transient ischaemic attack and minor stroke (SUPPORT TIA): Protocol for a feasibility study and process evaluation

Journal:	*BMJ Open*
Manuscript ID	bmjopen-2021-060280.R2
Article Type:	Protocol
Date Submitted by the Author:	13-May-2022

Complete List of Authors:	Turner, Grace; Institute of Applied Health Research University of Birmingham; University of Birmingham<br>Jones, Rachael; Royal Wolverhampton NHS Trust<br>Collis, Phillip; Institute of Applied Health Research University of Birmingham; University of Birmingham<br>Patel, Smitaa; University of Birmingham, Birmingham Clinical Trials Unit<br>Jowett, Sue; University of Birmingham, Health Economics Unit<br>Tearne, Sarah ; University of Birmingham Clinical Trials Unit<br>Foy, Robbie; University of Leeds, Leeds Institute of Health Sciences<br>Atkins, Lou; UCL<br>Mant, Jonathan; University of Cambridge, General Practice and Primary Care Research Unit<br>Calvert , Melanie ; University of Birmingham, Centre for Patient Reported Outcomes Research and Institute of Applied Health Research

**Primary Subject Heading**:	Cardiovascular medicine
Secondary Subject Heading:	Research methods
Keywords:	STROKE MEDICINE, REHABILITATION MEDICINE, Organisation of health services < HEALTH SERVICES ADMINISTRATION & MANAGEMENT, Protocols & guidelines < HEALTH SERVICES ADMINISTRATION & MANAGEMENT, Depression & mood disorders < PSYCHIATRY, QUALITATIVE RESEARCH

SCHOLARONE™
Manuscripts

Structured follow-up pathway to support people after transient ischaemic attack and minor stroke (SUPPORT TIA): Protocol for a feasibility study and process evaluation

Grace M Turner* 1, 2

Rachael Jones 3, 4

Phillip Collis 1, 2

Smitaa Patel 6

Sue Jowett 7

Sarah Tearne 6

Robbie Foy 8

Lou Atkins 9

Jonathan Mant 10

Melanie Calvert 1, 2, 11, 12, 13, 14

1 Institute of Applied Health Research, University of Birmingham, Birmingham, UK

2 Centre for Patient Reported Outcomes Research, University of Birmingham, Birmingham, UK

3 Royal Wolverhampton NHS Trust, Wolverhampton Rd, Heath Town, Wolverhampton, UK

4 Clinical Research Network West Midlands (CRN WM), UK

5 Patient partner, UK

6 Birmingham Clinical Trials Unit, University of Birmingham, Birmingham, UK

7 Health Economics Unit, University of Birmingham, Birmingham, UK

8 Leeds Institute of Health Sciences, University of Leeds, Leeds, UK

9 Centre for Behaviour Change, University College London, London, UK

10 Department of Public Health and Primary Care, University of Cambridge, Cambridge, UK

11 NIHR Surgical Reconstruction and Microbiology Research Centre, University Hospitals Birmingham NHS Foundation Trust and University of Birmingham, Birmingham, UK

12 NIHR Birmingham Biomedical Research Centre, University Hospitals Birmingham NHS Foundation Trust and University of Birmingham, Birmingham, UK

For peer review only

For peer review only - http://bmjopen.bmj.com/site/about/guidelines.xhtml

13 Birmingham Health Partners Centre for Regulatory Science and Innovation, University of Birmingham, Birmingham, UK

14 NIHR Applied Research Collaboration (ARC) West Midlands, University of Birmingham, Birmingham, UK

* Corresponding author: Dr Grace Turner; Institute of Applied Health Research, University of Birmingham, Birmingham, B15 2TT, UK; G.Turner.1@bham.ac.uk

Word count

2,353

Key words

Transient ischaemic attack; mini stroke; TIA; minor stroke; rehabilitation, stroke prevention, follow-up, Hospital Anxiety and Depression Scale, Patient-Reported Outcomes Measurement Information System, 5-level EuroQol 5-Dimensions, Fatigue Assessment Scale, Patient Activation Measure-13, Medication Adherence Rating Scale -5

For peer review only

Abstract

Introduction

People who experience transient ischaemic attack (TIA) and minor stroke have limited follow-up despite rapid specialist review in hospital. This means they often have unmet needs and feel abandoned following discharge. Care needs after TIA/minor stroke include information provision (diagnosis and stroke risk), stroke prevention (medication and lifestyle change), and holistic care (residual problems and return to work or usual activities). This protocol describes a feasibility study and process evaluation of an intervention to support people after TIA/minor stroke. The study aims to assess the feasibility and acceptability of (i) the intervention and (ii) the trial procedures for a future randomised controlled trial of this intervention.

Methods and analysis

This is a multicentre, randomised (1:1) feasibility study with a mixed-methods process evaluation. Sixty participants will be recruited from TIA clinics or stroke wards at three hospital sites (England). Intervention arm participants will be offered a nurse or allied health professional-led follow-up appointment four weeks after TIA/minor stroke. The multifaceted intervention includes: a needs checklist, action plan, resources to support management of needs, a general practitioner letter, and training to deliver the intervention. Control arm participants will receive usual care. Follow-up will be self-completed questionnaires (12- and 24-weeks) and a clinic appointment (24-weeks). Follow-up questionnaires will measure anxiety, depression, fatigue, health related quality of life, self-efficacy and medication adherence. The clinic appointment will collect body mass index, blood pressure, cholesterol and medication. Assessment of feasibility and acceptability will include quantitative process variables (such as recruitment and questionnaire response rates), structured observations of study processes, and interviews with a sub-sample of participants and clinical staff.

Ethics and dissemination

Favourable ethical opinion was gained from the Wales Research Ethics Committee (REC) 1 (23/02/2021, REC reference: 21/WA/0036). Study results will be published in peer-reviewed journals and presented at conferences. A lay summary and dissemination strategy will be co-designed with consumers. The lay summary and journal publication will be distributed on social media.

Registration details

ISRCTN reference: ISRCTN39864003

For peer review only - http://bmjopen.bmj.com/site/about/guidelines.xhtml

Strengths and limitations of this study

- The multi-centre study will enable exploration of implementation of the intervention in the context of different sites.
- The process evaluation is underpinned by the National Institutes of Health's (NIH) Behavioural Change Consortium (BCC) treatment fidelity framework.
- Quantitative and qualitative methods will explore acceptability and how the intervention is implemented in practice.
- Participants must have the ability to converse in everyday English and read in English to participate, which may limit the generalisability of our findings.

For peer review only - http://bmjopen.bmj.com/site/about/guidelines.xhtml

Introduction

Transient ischaemic attack (TIA) and minor stroke are important risk factors for stroke. Over 46,000 people experience a first TIA or minor stroke per year in the United Kingdom(1), 240,000 in the United States(2) and 0.31 million in China(3).

National guidelines promote long-term management that focuses on stroke prevention.(4-6) However, research shows TIA and minor stroke patients feel unsupported in stroke prevention – both medication and lifestyle change – and often lack basic understanding of their diagnosis, stroke risk and preventative medication.(7) Furthermore, many people experience a wide variety of residual impairments and unmet needs after TIA or minor stroke, including anxiety, mood/ emotional impact, fatigue, cognitive impairment, physical weakness, visual impairment and impaired speech.(8-17) TIA and minor stroke have been also reported to impact on people's ability to return to work, performance at work, social activities and family relationships.(12-19) Follow-up care is variable and often inadequate with patients feeling abandoned after hospital discharge.(7)

Care needs after TIA and minor stroke include information provision (diagnosis and stroke risk); stroke prevention (medication and lifestyle change); and holistic care (residual problems and return to work or usual activities).(7) However, there is no evidence for how to best support these patients after rapid specialist review in hospital. To address this, we developed a multifaceted intervention which aims to actively identify and address unmet needs after TIA and minor stroke: SUPPORT TIA (Structured follow-Up Pathway to imProve management Of Residual impairmenTs and patients' quality of life after Transient Ischaemic Attack and minor stroke). The components of the intervention are described in this protocol. In accordance with the Medical Research Council (MRC) guidance on developing and evaluating complex interventions,(20) we will evaluate the feasibility and acceptability of (i) the intervention and (ii) the trial procedures for a future randomised controlled trial (RCT) of this intervention. In addition, we will conduct a process evaluation to evaluate intervention fidelity and contextual influences on delivery.

Methods and analysis

Study design

The study is a multicentre, individual randomised feasibility study with a mixed-methods process evaluation. The trial is registered on the ISRCTN registry (Reference: ISRCTN39864003). The study is reported in accordance with the SPIRIT checklist(21) and the design is summarised in Figure 1.

The study opened for recruitment in September 2021 with planned completion by December 2022.

Patient and public involvement

A core group of three people who have experienced TIA or minor stroke have supported this study from inception, with *ad hoc* contributions from other members of the public with TIA or minor stroke. The group supported the initial development of the research question and funding application, which were informed by their priorities and experiences. The group was involved in:

For peer review only - http://bmjopen.bmj.com/site/about/guidelines.xhtml

selection of outcome measures; development of study documents; and design of the trial, such as recruitment strategies and considering participant burden related to data collection and attending intervention appointments. The group was integral to the intervention development, in particular the website of support services and resources. The group will continue to support the delivery of the study and dissemination of findings. One member (PC) is a co-author and member of the Study Oversight Committee. Patient and public involvement activities and impact will be reported using GRIPP2.(22)

Study objectives

Trial design and methods

a) Assess feasibility and acceptability of the trial design and methods, including: number of patients meeting eligibility criteria; consent and randomisation processes; recruitment and retention rates; piloting the health economics questionnaire; and data completeness;
b) Provide data to inform the sample size for a definitive randomised controlled trial (RCT);
c) Provide data to help inform selection of the primary outcome measure for a definitive RCT, including data completeness and correlation of the outcome measures with each other.

Intervention (process evaluation)

d) Investigate acceptability of the intervention for participants and intervention providers;
e) Test hypotheses relating to the theoretical underpinning of the intervention;
f) Assess if intervention providers are adequately trained to deliver the intervention;
g) Assess adherence to the intervention;
h) Assess contamination with the control group;
i) Define the "dose" of the intervention (i.e. attendance, length of appointment and number of appointments);
j) Explore how well intervention participants received and understood the intervention;
k) Explore to what extent the intervention was enacted as intended by patient participants (intervention group).

Study setting and eligibility criteria

Participants will be recruited from TIA clinics and stroke wards at three tertiary hospital sites in England, one in South East England (Berkshire) and two in North West England (Wigan and Liverpool). Participants will be adults who have experienced a first or recurrent TIA or minor stroke. The full eligibility criteria are detailed in Box 1.

Box 1: Eligibility criteria

Inclusion criteria

- Adults (aged ≥18 years);
- Resident in England;
- Diagnosis of confirmed TIA or minor stroke by a stroke consultant. TIA will be defined as a transient episode of neurological dysfunction caused by focal brain, spinal cord, or retinal ischemia, without acute infarction.(23) Minor stroke will be defined as a modified Rankin scale score ≤1 or no change in modified Rankin scale score from pre-event (to account for people who were disabled prior to their TIA or minor stroke)*;

For peer review only

- Attending the TIA clinic or stroke ward for a new diagnosis of TIA or minor stroke, rather than for a follow-up appointment;
- Ability to converse in everyday English and read in English;
- Capacity to provide fully informed consent for participation in the trial.

Exclusion criteria

- History of full stroke (modified Rankin scale score >1);
- History of dementia;
- People who lack capacity to participate, such as if they have severe memory problems that mean they would not remember giving consent or if they have severe communication problems, not precluding patients who use electronic devices to communicate;
- Patients receiving early supported discharge or cardiac rehabilitation;
- Patients receiving any palliative care.

* There is no standardised definition of minor stroke. Our criteria were selected as a practical definition to identify people with good functional recovery after stroke.(24)

Intervention

Intervention development was underpinned by the Behaviour Change Wheel theoretical framework(25) and iteratively refined in collaboration with patient partners and a multidisciplinary team (Appendix 1, eTable 1)

The multifaceted intervention broadly comprises six components (Figure 2):

1. Training for nurses and allied health professionals (AHPs) delivering the intervention;
2. Structured nurse or AHP led follow-up appointment, 4 weeks after TIA or minor stroke;
3. Needs checklist completed by participants prior to the appointment;
4. Resources to support management of needs, including a website of resources and support services; list of local support services; and a self-management booklet;
5. Action plan;
6. Structured letter to GPs to improve the interface communication between secondary and primary care.

Participants will also receive usual care and a Stroke Association TIA information sheet. Follow-up for TIA and minor stroke is not standardised; therefore, usual care varies between hospitals, GP practices and individual clinicians. Typically, any secondary care follow-up is related to imaging and investigations to determine cause of the TIA/ minor stroke and inform stroke risk prediction; for example, carotid imaging or electrocardiogram. Follow-up in primary care usually focuses on secondary prevention, such as medication and lifestyle advice; however, presence and quality of primary care follow-up post-TIA/ minor stroke is variable.

Details of the intervention are described below in accordance with the template for intervention description and replication (TIDieR) checklist.(26) The logic model is depicted in Figure 3.

Figure 3: Logic model

For peer review only - http://bmjopen.bmj.com/site/about/guidelines.xhtml

Materials and procedures

Participants randomised to receive the intervention will be invited to a nurse/AHP-led follow-up appointment. Prior to their appointment, participants will be asked to complete a needs checklist, which will be posted to them prior to the appointment. The checklist comprises 12 potential needs which encompass information provision (diagnosis and stroke risk); secondary stroke prevention (medication and lifestyle change); and holistic care (psychological and psychosocial) (online supplementary appendix 2). The checklist is an adapted version of the Stroke Review Checklist(27) and was informed by the literature and earlier qualitative research,(7) co-designed with consumers.

The nurse/AHP will use the checklist to guide discussions to identify participants' unmet needs. If multiple needs are identified, priority will be given to addressing needs which the participant considers the most significant.

The nurse/AHP will address needs that can be resolved during the appointment, such as information about driving. For needs that cannot be immediately addressed, the nurse/AHP will, where appropriate, refer or signpost to support services and develop an action plan which will be agreed with the participant. Where possible, the nurse/AHP will make referrals; however, in some circumstances GP referral may be required, in which case this will be requested in the GP letter. To facilitate this, the intervention provider will be provided with a website of resources and support services and a list of local services.

The nurse/ AHP will take the participants' blood pressure and, if raised (≥140/90 mmHg), request for the participant's GP to review blood pressure in the action plan and GP letter.

If necessary, the nurse/AHP may invite the participant to attend another follow-up appointment, at a suitable time point, to monitor the participant's progress and revise the action plan if required. These additional follow-ups may be conducted by telephone, video call or face-to-face.

A letter will be sent to the participant's GP along with a copy of the agreed action plan. A letter template will include recommended GP actions, a summary of the appointment and actions taken.

The participant will be provided with a self-management booklet (an abridged version of the resources and services website) and a copy of the action plan and GP letter.

Intervention provider

The intervention will be delivered by a nurse or AHP, with stroke expertise, who are clinical staff at the participating hospital sites. It is anticipated that 1-2 intervention providers will be trained per site; however, this will depend on availability of clinical staff at sties. The nurses and AHPs will attend training which will include education about potential needs after TIA and minor stroke, and how to deliver the intervention. One training session, approximately 2.5 hours) will be provided remotely (via Zoom); however, ad hoc support and feedback will be encouraged after the training.

Setting and modes of delivery

The intervention appointments will be delivered at the site's TIA clinic, either face-to-face or remotely (e.g. telephone or video call). Face-to-face delivery will be preferable where possible.

When and how often

The intervention appointment will take place at four weeks (or up to six weeks). The appointment is expected to last approximately 30 minutes. One appointment will be offered initially; however, participants will have an option to attend additional follow-up if judged clinically necessary by the

For peer review only - http://bmjopen.bmj.com/site/about/guidelines.xhtml

nurse or AHP. There are no pre-determined criteria for further follow-up and the criteria used by nurses/AHPs will be recorded as part of the feasibility study to inform future refinement of the intervention.

Control arm

The control group will receive usual care and be given a Stroke Association TIA information sheet when they are informed about their allocation to the control arm.

Recruitment

A member of the clinical team will screen patients' medical records and approach potentially eligible patients face-to-face or by phone. After confirming eligibility, potential participants will be invited to take part in the study. Informed consent may be taken face-to-face (for people approached in clinic), by post (for people who need more time to consider participation) or verbally (for people approached via phone). Verbal consent will be clearly documented in the participant's medical records and the participant will also be sent a postal consent form to compete. Sites will receive a per-participant reimbursement for recruitment.

Sample size

The study will aim to recruit 60 participants (30 in the intervention group, 30 in the control group). As this is a feasibility study, no formal sample size calculation has been performed; however, the sample size is the estimated number that would be feasible to show that we can recruit these types of patients for this type of study.(28)

Randomisation

Participants will be randomised 1:1 to either the intervention or control group. A minimisation algorithm will be used within an online randomisation system to ensure balance in the treatment allocation using the following variables: age at consent (<60 years, ≥60 years); sex (male, female); diagnosis (TIA, minor stroke); employment (employed, non-employed/retired).

A 'random element' will be included in the minimisation algorithm, so that each patient has a probability, of being randomised to the opposite treatment that they would have otherwise received.

Participants will be randomised at baseline by clinical staff; however, to prevent baseline patient reported outcomes being affected by study arm allocation, participants will be notified of their randomisation allocation after they have returned the 1-week questionnaire or at 3 weeks (if the 1-week questionnaire is not returned). Participants will be notified of their allocation by a letter in the post, which will be sent by the research team at the Trials Unit. Due to the nature of the intervention, it is not possible to blind participants or clinicians delivering the intervention.

Outcomes and data collection

Table 1 summarises the patient reported, health economic and clinical outcome measures. Contact details, demographic information and medical history will be collected at baseline from medical records or participant interview, by a member of clinical staff. Questionnaires comprising Patient Reported Outcome Measures (PROMs) (Table 2) will be completed by participants, either by post or electronically, at 1, 12 and 24 weeks. PROM rationale for assessment and psychometric properties are presented in eTable 2, see appendix 3. Questionnaires at 12 and 24 weeks will also collect health

For peer review only - http://bmjopen.bmj.com/site/about/guidelines.xhtml

economics data. The first PROM completion will be at one week rather than baseline due to the nature of the PROM questions and to reduce burden on participants. Clinical data (Table 2) will be collected at an end of study clinic appointment at 26 weeks by a research nurse or clinical staff. Where possible, this appointment will be face-to-face in the TIA clinic; however, may be delivered remotely if face-to-face is not an option.

Table 1: summary of patient reported, health economic and clinical outcome measures.

	Data	Timepoint
**Baseline data**	Contact details<br>Demographic: date of birth, sex, ethnicity, employment status<br>Medical: diagnosis, date of TIA or minor stroke, modified Rankin scale score, length of stay, smoking status, alcohol consumption, height, weight, body mass index, comorbidities, medication, blood pressure, cholesterol	Baseline
**Patient reported outcome measure**	Health related quality of life: Patient-Reported Outcomes Measurement Information System (PROMIS)-Global Health 10<br>Health related quality of life: 5-level EuroQol 5-Dimensions (EQ-5D-5L)<br>Anxiety/ depression: Hospital Anxiety and Depression Scale (HADS)<br>Fatigue: Fatigue Assessment Scale (FAS)<br>Self-efficacy: Patient Activation Measure-13 (PAM-13)<br>Medication adherence: Medication Adherence Rating Scale -5 (MARS)<br>Satisfaction with overall care after TIA/minor stroke question: 5-point Likert scale (very satisfied – very dissatisfied)	1, 12 and 24 weeks
**Health economics**	Use of healthcare services<br>Change in employment status, altered work hours and days off sick<br>Other costs incurred because of TIA or minor stroke	12 and 24 weeks
**Clinical data**	Body Mass Index<br>Blood pressure<br>Bloods: cholesterol<br>Medications	Baseline and 26 weeks

Feasibility outcomes and process evaluation

The feasibility study and process evaluation outcomes are detailed in Tables 2 and 3. The process evaluation is underpinned by the National Institutes of Health's (NIH) Behavioural Change Consortium (BCC) treatment fidelity framework.(29) This framework includes five domains of treatment fidelity: Study Design, Training, Delivery, Receipt and Enactment.

Table 2: Feasibility outcomes and measurement of outcomes

For peer review only - http://bmjopen.bmj.com/site/about/guidelines.xhtml

Objective	Feasibility outcomes	Measurement of outcome
a) Assess feasibility and acceptability of the trial design and methods	Number of eligible/ineligible patients and reasons for ineligibility	Recruitment log
	Proportion of participants who consent face-to-face, verbal or postal	Registration log: method of consent
	Willingness of clinical staff to randomise patients	Interviews (clinical staff involved in randomisation)
	Recruitment and attrition rates	Registration log
	Response rates and frequencies of missing data: participant completed questionnaires and case report forms	1, 12 and 24 week questionnaires<br>Case Report forms
	End of study clinic appointment attendance rates	End of Study Clinic Appointment Form
	Acceptability of the trial design	Interviews (participants and clinical staff)<br>Structured observations
b) Provide data to inform the sample size for a definitive randomised controlled trial	Standard deviations of continuous patient reported outcome measures at six months	Patient reported outcome measure scores
	Recruitment and attrition rates	Registration log
c) Provide data to help inform selection of the primary outcome measure for a definitive randomised controlled trial	Correlation of patient reported outcome measures	Patient reported outcome measure scores
	Patient reported outcome measure response rates and missing data	1, 12 and 24 week questionnaires

Table 3: Process evaluations outcomes and measurement of outcomes

NIH BCC domain	Objective	Outcome	Measurement of outcome
**Study design**	d) Investigate acceptability of the intervention for participants and intervention providers	Participants' and intervention providers' opinion on acceptability of the intervention	Interviews (participants and intervention providers)<br>Feedback questionnaire (intervention participants)
	e) Test hypotheses relating to the theoretical underpinning of the intervention	Participants' satisfaction with identification and management of needs	Interviews (participants and intervention providers)<br>Feedback questionnaire (intervention participants)
		Participants acting on agreed action plans and/or accessing support services	Interviews (participants)

For peer review only - http://bmjopen.bmj.com/site/about/guidelines.xhtml

**Training**	f) Assess if intervention providers are adequately trained to deliver the intervention	Intervention providers' understanding of the intervention components	Interviews (intervention providers)
**Delivery**	g) Assess adherence to the intervention	Intervention providers' adherence to and deviations from the intervention manual	Structured observations<br>Intervention log
	h) Assess contamination with the control group	Control group contamination	Interviews (participants and clinical staff)<br>Structured observations
	i) Define the "dose" of the intervention	Intervention follow-up appointment: attendance, length of appointment and number of appointments	Intervention log
**Receipt**	j) Explore how well intervention participants received and understood the intervention	Participants' perception of the intervention	Interviews (participants)<br>Feedback questionnaire (intervention participants)
**Enactment**	k) Explore to what extent the intervention was enacted as intended by intervention participants	Participants acting on agreed action plans and/or accessing support services	Interviews (intervention participants)

Case report forms

The following case report forms will collect data on feasibility outcomes: recruitment log (recruitment rates and reasons for ineligibility); registration log (method of consent: face-to-face/ verbal/ postal); intervention log (attendance rates, duration, number of appointments per participant); end of study clinic appointment form (attendance). Case report forms will be assessed for missing data. The following intervention documents will capture information on needs, what was discussed and action plans: checklist, action plan and GP letter.

Participant completed questionnaires

Participant completed questionnaires (1, 12 and 24 weeks) will be analysed for response rates and missing data. Standard deviations of continuous PROMs at six months and correlation of PROMs will inform the sample size and selection of outcome measures for the definitive RCT. The intervention feedback questionnaire will report acceptability of the intervention. A paper copy of the feedback questionnaire and pre-paid envelope will be posted to participants after the intervention appointment. This questionnaire contains 5-point Likert scale questions (e.g. strongly agree – strongly disagree) and free text questions about experiences of the checklist, appointment and action plan.

Structured observations

A member of the study team will observe the following study processes: recruitment and consent procedures; intervention appointments; and end of study clinic appointments. Both face-to-face and

For peer review only - http://bmjopen.bmj.com/site/about/guidelines.xhtml

remote modes of delivery will be observed for these procedures if possible. A target of three observations will be conducted for recruitment/ consent and end of study clinic appointments (one at each site). A target of two intervention appointments will be observed per site (20%). More observations may be conducted if deemed necessary; for example, multiple clinical staff performing each procedure. A pragmatic approach will be taken to select which sessions to observe based on the availability of the research and clinical teams. A checklist will be used to document adherence to the protocol and field notes will be collected.

Audit

At the end of the recruitment period, each site will perform an audit to identify the total number of confirmed TIA and minor stroke patients who attended the TIA clinic or stroke ward during the recruitment period. The age and sex of these patients will also be collected. This data will be used to compare average age and sex of patients recruited to the trial against patients not recruited.

Qualitative interviews

At the end of the study, semi-structured interviews will be conducted with a subset of participants and clinical staff involved in recruitment and/or intervention delivery. The sample size is anticipated to be 8-10 patients and 4-6 clinical staff (including those involved in recruitment/consent, intervention delivery and end of study clinic appointments). For patient participants, convenience sampling will be used initially; however, sampling will become increasingly purposeful to achieve variation in age (<60 years, ≥60 years) and diagnosis (TIA, minor stroke). For clinical staff, convenience sampling will be used. Interviews will be conducted by GT, an experienced qualitative researcher. Interviews will be face-to-face (home/ hospital), telephone or video call, depending on the participants preference. Interviews will explore acceptability of the intervention and trial design. Semi-structured topic guides will include discussion of the following:

- Patient participants:
    - Intervention: intervention and trial design acceptability; how well intervention participants received and understood the intervention; extent to which intervention providers addressed needs; if the action plan was actioned; facilitators and barriers to enactment.
    - Control: trial design acceptability; intervention contamination.
    - Both: what care/support participants received; understanding what comprised usual care.
- Staff participants: acceptability of the trial design; experience of training day and understanding of the intervention; acceptability of delivering the intervention; facilitators and barriers to implementing both the trial design and the intervention; and experience of contamination with the control group.

Monitoring, adverse events and study oversight

Information on trial monitoring, adverse events and study oversight is presented in Appendix 4.

Analysis

Quantitative outcomes will be analysed using simple descriptive statistics (e.g. proportions and percentages, mean and standard deviations) and where appropriate, point estimates of effect sizes (e.g. mean differences and relative risks) and associated 95% confidence intervals. Analyses comparing the intervention and control groups will use the intention to treat principle, i.e. all

For peer review only - http://bmjopen.bmj.com/site/about/guidelines.xhtml

participants will be analysed in the treatment group to which they were randomised irrespective of compliance or other protocol deviation. Analysis will be conducted using Stata v16.

For qualitative data, interviews will be audio recorded and transcribed verbatim. NVivo v12 will be used to manage, sort, code and organise the anonymised transcribed data. Interview transcripts will be analysed by GT using directed thematic analysis, using Braun and Clarke's 6-stage process,(30) informed by the research aims.(31)

The health economics analysis will assess completion rates, estimate resources required to deliver the intervention and report simple descriptive statistics for resource use and outcomes. Key resource use items not currently specified on the form but included by participants will also be identified. The information will inform the cost and outcome data collection and identification of unit costs for a larger trial.

As this project is a training fellowship, the fellow (GT) will conduct the analysis and will have access to the whole dataset in order to conduct the trial. Therefore, it is not possible to conducted blinded analyses.

Data will be made available upon reasonable request.

Progression criteria

The pre-defined progression criteria, detailed in Table 4, will be used to inform a decision on whether a full RCT is warranted and feasible. The criteria were agreed by the Study Oversight Committee and follow a traffic light system using quantitative measures supported by qualitative data.

Table 4: Progression criteria

Key uncertainties	Measures used	Progression criteria
**Trial design**		
Recruitment	% target sample size recruited	● ≥90%: proceed to a full-scale trial<br>● 70-89%: SOC will consider the feasibility of proceeding to a full-scale trial bearing in mind the data presented, representativeness of the sample, and possible steps to increase recruitment<br>● <70%: full-scale trial unlikely to be feasible
Randomisation*	% of consented participants randomised	● ≥90%: proceed to a full-scale trial<br>● 70-89%: SOC will consider the feasibility of proceeding to a full-scale trial bearing in mind the data presented, representativeness of the sample, and possible steps to address randomisation issues<br>● <70%: full-scale trial unlikely to be feasible
Return rate of 24-week questionnaire*	% of 24-week questionnaires returned	● ≥80%: proceed to a full-scale trial<br>● 50-79%: SOC will consider the feasibility of proceeding to a full-scale trial bearing in mind

For peer review only - http://bmjopen.bmj.com/site/about/guidelines.xhtml

		the data presented, representativeness of the sample, and possible steps to increase return rates<br>● <50%: full-scale trial unlikely to be feasible
**Intervention**		
Attendance rate*	% of intervention arm participants attending first appointment	● ≥90%: proceed to a full-scale trial<br>● 70-89%: SOC will consider the feasibility of proceeding to a full-scale trial bearing in mind the data presented, representativeness of the sample, and possible steps to increase attendance<br>● <70%: full-scale trial unlikely to be feasible
Delivery of the intervention	% completion of: checklists, action plans, GP letters; use of directory of support services; Issues regarding delivery of the intervention components and contamination explored in qualitative interviews	The SOC will consider the quantitative and qualitative data and make an overall judgement on whether the intervention content is delivered as intended
Acceptability	% of participants reporting acceptability of intervention components on intervention feedback questionnaire; Issues regarding acceptability of the intervention components explored in qualitative interviews	The SOC will consider the quantitative and qualitative data and make an overall judgement on whether the intervention is acceptable

* critical progression criteria: the trial is unlikely to be feasible if these criteria are not met, even if other criteria are satisfactory.
GP: General Practitioner; SOC: Study Oversite Committee; %: percent

Ethics and dissemination

Favourable ethical opinion was gained from the Wales Research Ethics Committee (REC) 1 (23/02/2021, REC reference: 21/WA/0036). Study results will be published in a peer-reviewed journal and presented at relevant conferences. A lay summary and dissemination strategy will be co-designed with consumers. The lay summary and peer review publication will be distributed on social media.

Authors' contributions

GT, MC, JM, RF and LA contributed to the conception of the study. The design of the feasibility study was led by GT with contributions from MC, JM, RF, LA, PC and SP, SJ and ST. The design of the intervention was led by GT with contributions from MC, JM, RF, LA, PC and RJ. GT wrote the first draft of the manuscript. All authors contributed to and approved the final version.

For peer review only

For peer review only - http://bmjopen.bmj.com/site/about/guidelines.xhtml

Acknowledgments

We thank the patient and public involvement group members who have provided valuable insight and feedback into the intervention and study design: Phil Collis, Lesley Thomson and Sally Hughes. We thank those involved in the development of the intervention with special mention to Kirsty Price and Joanne Chesson.

Funding statement

This work was supported by National Institute for Health Research (NIHR) Post-Doctoral Fellowship Scheme grant number PDF-2017-10-047. The views expressed are those of the author(s) and not necessarily those of the NIHR or the Department of Health and Social Care.

Competing interests

MC and JM are National Institute for Health Research (NIHR) Senior Investigators. MC also receives funding from the National Institute for Health Research (NIHR), UK Research and Innovation (UKRI), the NIHR Birmingham Biomedical Research Centre, NIHR Surgical Reconstruction and Microbiology Research Centre and NIHR ARC West Midlands at the University of Birmingham and University Hospitals Birmingham NHS Foundation Trust, UK SPINR (UKRI) Health Data Research UK, Innovate UK (part of UKRI), Macmillan Cancer Support, UCB Pharma, GSK, Gilead and Janssen. MC has received personal fees from Astellas, Aparito Ltd, CIA Oncology, Takeda, Merck, Daiichi Sankyo, Glaukos, GSK and the Patient-Centered Outcomes Research Institute (PCORI) outside the submitted work. The views expressed in this publication are those of the author(s) and not necessarily those of the NIHR or the Department of Health and Social Care. JM has done consultancy work for BMS/Pfizer, Omron, and Doctorlink Innovations Ltd. GMT, RJ, PC, SP, SJ, ST, RF and LA declare no conflict of interest.

References

1. Scarborough P, Peto V, Bhatnagar P, Kaur A, Leal J, Luengo-Fernandez R, et al. Stroke statistics. British Heart Foundation and Stroke Association: London; 2009.
2. American Stroke Association. Let's talk about Transient Ischemic Attack (TIA) [Available from: https://www.stroke.org/-/media/Stroke-Files/Lets-Talk-About-Stroke/Type-of-Stroke/LTAS_TransientIschemicAttack_2019.pdf.
3. Jiang B, Sun H, Ru X, Sun D, Chen Z, Liu H, et al. Prevalence, Incidence, Prognosis, Early Stroke Risk, and Stroke-Related Prognostic Factors of Definite or Probable Transient Ischemic Attacks in China, 2013. Front Neurol. 2017;8:309.
4. European Stroke Organisation (ESO) Executive Committee. Guidelines for management of ischaemic stroke and transient ischaemic attack 2008. Cerebrovascular diseases (Basel, Switzerland). 2008;25(5):457-507.
5. Intercollegiate Stroke Working Party. National clinical guideline for stroke. London: Royal College of Physicians; 2016.
6. Kernan WN, Ovbiagele B, Black HR, Bravata DM, Chimowitz MI, Ezekowitz MD, et al. Guidelines for the Prevention of Stroke in Patients With Stroke and Transient Ischemic Attack. Stroke. 2014;45(7):2160-236.
7. Turner GM, McMullan C, Atkins L, Foy R, Mant J, Calvert M. TIA and minor stroke: a qualitative study of long-term impact and experiences of follow-up care. BMC Family Practice. 2019;20(1):176.

For peer review only - http://bmjopen.bmj.com/site/about/guidelines.xhtml

8. Moran GM, Fletcher B, Feltham MG, Calvert M, Sackley C, Marshall T. Fatigue, psychological and cognitive impairment following transient ischaemic attack and minor stroke: a systematic review. European journal of neurology. 2014;21(10):1258-67.
9. Sackley CM, Mant J, McManus RJ, Humphreys G, Sharp L, Mares K, et al. Functional and emotional outcomes after transient ischemic attack: A 12-month prospective controlled cohort study. International journal of stroke. 2019:1747493018823158.
10. Turner GM, Calvert M, Feltham MG, Ryan R, Marshall T. Ongoing impairments following transient ischaemic attack: retrospective cohort study. European journal of neurology. 2016;23(11):1642-50.
11. van Rooij FG, Kessels RP, Richard E, De Leeuw FE, van Dijk EJ. Cognitive Impairment in Transient Ischemic Attack Patients: A Systematic Review. Cerebrovascular diseases (Basel, Switzerland). 2016;42(1-2):1-9.
12. Gibson J, Watkins C. People's experiences of the impact of transient ischaemic attack and its consequences: qualitative study. Journal of advanced nursing. 2012;68(8):1707-15.
13. Green TL, King KM. Experiences of male patients and wife-caregivers in the first year post-discharge following minor stroke: a descriptive qualitative study. International journal of nursing studies. 2009;46(9):1194-200.
14. Kamara S, Singh S. What are the patient-held illness beliefs after a transient ischaemic attack, and do they determine secondary prevention activities: an exploratory study in a North London General Practice. Primary health care research & development. 2012;13(2):165-74.
15. Rochette A, Desrosiers J, Bravo G, St-Cyr-Tribble D, Bourget A. Changes in participation after a mild stroke: quantitative and qualitative perspectives. Topics in stroke rehabilitation. 2007;14(3):59-68.
16. Spurgeon L, James G, Sackley C. Subjective experiences of transient ischaemic attack: a repertory grid approach. Disability and rehabilitation. 2013;35(26):2205-12.
17. Taule T, Raheim M. Life changed existentially: a qualitative study of experiences at 6-8 months after mild stroke. Disability and rehabilitation. 2014;36(25):2107-19.
18. Carlsson GE, Moller A, Blomstrand C. Managing an everyday life of uncertainty--a qualitative study of coping in persons with mild stroke. Disability and rehabilitation. 2009;31(10):773-82.
19. Croot EJ, Ryan TW, Read J, Campbell F, O'Cathain A, Venables G. Transient ischaemic attack: a qualitative study of the long term consequences for patients. BMC Fam Pract. 2014;15:174.
20. Skivington K, Matthews L, Simpson SA, Craig P, Baird J, Blazeby JM, et al. A new framework for developing and evaluating complex interventions: update of Medical Research Council guidance. BMJ. 2021;374:n2061.
21. Chan A-W TJ, Altman DG, Laupacis A, Gøtzsche PC, Krleža-Jerić K, Hróbjartsson A, Mann H, Dickersin K, Berlin J, Doré C, Parulekar W, Summerskill W, Groves T, Schulz K, Sox H, Rockhold FW, Rennie D, Moher D. SPIRIT 2013 Statement: Defining Standard Protocol Items for Clinical Trials. Annals of Internal Medicine. 2013;158(3):200-7.
22. Staniszewska S, Brett J, Simera I, Seers K, Mockford C, Goodlad S, et al. GRIPP2 reporting checklists: tools to improve reporting of patient and public involvement in research. BMJ. 2017;358:j3453.
23. Easton JD, Saver JL, Albers GW, Alberts MJ, Chaturvedi S, Feldmann E, et al. Definition and evaluation of transient ischemic attack: a scientific statement for healthcare professionals from the American Heart Association/American Stroke Association Stroke Council; Council on Cardiovascular Surgery and Anesthesia; Council on Cardiovascular Radiology and Intervention; Council on Cardiovascular Nursing; and the Interdisciplinary Council on Peripheral Vascular Disease. The American Academy of Neurology affirms the value of this statement as an educational tool for neurologists. Stroke. 2009;40(6):2276-93.
24. Fischer U, Baumgartner A, Arnold M, Nedeltchev K, Gralla J, Marco De Marchis G, et al. What Is a Minor Stroke? Stroke. 2010;41(4):661-6.

For peer review only - http://bmjopen.bmj.com/site/about/guidelines.xhtml

25. Michie S, van Stralen MM, West R. The behaviour change wheel: A new method for characterising and designing behaviour change interventions. Implementation Science. 2011;6(1):42.
26. Hoffmann TC, Glasziou PP, Boutron I, Milne R, Perera R, Moher D, et al. Better reporting of interventions: template for intervention description and replication (TIDieR) checklist and guide. BMJ : British Medical Journal. 2014;348:g1687.
27. Turner GM, Mullis R, Lim L, Kreit L, Mant J. Using a checklist to facilitate management of long-term care needs after stroke: insights from focus groups and a feasibility study. BMC Family Practice. 2019;20(1):2.
28. Teare MD, Dimairo M, Shephard N, Hayman A, Whitehead A, Walters SJ. Sample size requirements to estimate key design parameters from external pilot randomised controlled trials: a simulation study. Trials. 2014;15(1):264.
29. Bellg AJ, Borrelli B, Resnick B, Hecht J, Minicucci DS, Ory M, et al. Enhancing treatment fidelity in health behavior change studies: best practices and recommendations from the NIH Behavior Change Consortium. Health Psychol. 2004;23(5):443-51.
30. Braun V, Clarke V. Using thematic analysis in psychology. Qualitative Research in Psychology. 2006;3(2):77-101.
31. Fereday J, Muir-Cochrane E. Demonstrating Rigor Using Thematic Analysis: A Hybrid Approach of Inductive and Deductive Coding and Theme Development. International Journal of Qualitative Methods. 2006;5(1):80-92.

Figure legends

Figure 1: Trial schema

Figure 2: Summary of the intervention components (AHP: Allied Health Professional; GP: General Practitioner)

Figure 3: Logic model

For peer review only

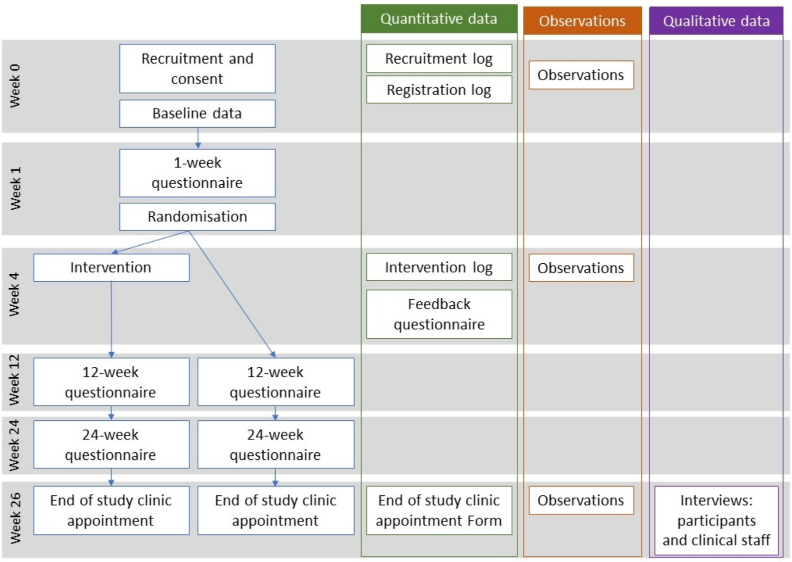

Figure 1

338x190mm (96 x 96 DPI)

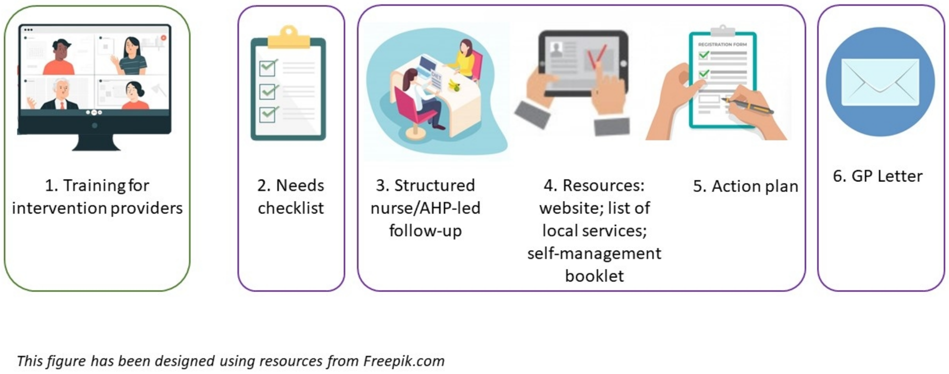

Figure 2

338x190mm (96 x 96 DPI)

Intervention component	Mechanism of change	Outputs	Potential outcomes
**1. Training for nurses or AHPs delivering the intervention.**	HCPs educated about potential needs after TIA or minor stroke, including health and social consequences.<br>HCPs instructed how to identify and address potential unmet needs, including use of intervention materials.	↑ HCPs knowledge of potential needs and strategies to identify and address needs.	***Short term***<br>• ↑ satisfaction with care.<br>• Access relevant further support (e.g. GP, support service).<br>• Access relevant resources (e.g. websites, apps).<br>• ↑ knowledge and understanding of diagnosis and stroke risk.<br>***Medium term***<br>• ↑ medication adherence.<br>• ↑ confidence and ability to self-manage needs.<br>***Long term***<br>• ↑ health related quality of life.<br>• ↓ or improved residual problems (e.g. anxiety, fatigue).<br>• ↓ in stroke risk factors.
**2. Structured nurse or AHP led follow-up appointment, 4 weeks after TIA or minor stroke.**	Service to provide patients with access to nurse or AHP follow-up.	Patients access holistic care and support for needs related to their TIA or minor stroke. Needs may be actioned immediately (e.g. driving information, reassurance) or an agreed plan for self-management or further support.	
**3. Needs checklist completed by participants prior to the appointment.**	Checklist provides patients with the opportunity to reflect on their needs and facilitates communication with the HCP.	Patients' needs are actively identified and acknowledged by the HCP.	
**4. Resources to support management of needs, including a website of resources and support services; list of local support services; and a self-management booklet.**	↑ knowledge of relevant support services and resources.	Patients are referred or signposted to relevant support services.<br>Patients are recommended relevant resources for their individual needs (e.g. websites, apps).	
**5. Action plan.**	Provides an opportunity for shared decision making and goal setting, and empowers patient to access services or resources and/or self-manage.	Patients are given clear actions to self-manage needs or access further support.	
**6. Structured GP letter**	↑ communication between secondary and primary care, and patients and GPs. Patients are empowered to access GP support.	GPs have better understanding of their patients' needs and care received. GPs receive clear recommendations for how to further support patient needs.	

AHP: Allied Health Professional; GP: General Practitioner; HCP: Healthcare provider; TIA: Transient Ischaemic Attack; ↑: increase; ↓: decrease

Figure 3

233x160mm (96 x 96 DPI)

Summary of intervention development, underpinned by the theoretical framework - Behaviour Change Wheel

- **Select target behaviour:** Healthcare provider identifying and addressing needs post-TIA/ minor stroke.
- **Identify what needs to change:**
  - Semi-structured interviews were conducted with healthcare providers and patients to identify influences on the target behaviour (results reported elsewhere). Transcripts were coded using the Theoretical Domains Framework (TDF).
- **Map TDF domains to "intervention functions"**
  - Four relevant intervention function identified: Education, Training, Environmental restructuring, Enablement.
- **Identify Behaviour Change Techniques (BCT):**
  - The BCT Taxonomy was used to identity appropriate BCT that mapped to relevant intervention functions.

The above process was informed by relevant literature; iterative feedback from patient partners and a multidisciplinary team (nurses, allied health professionals, GPs, consultants, researcher); and consideration of the APEASE criteria (Affordability, Practicality, Effectiveness and cost-effectiveness, Acceptability, Side-effects/safety, and Equity).

eTable 1: Summary of the barriers/ enablers mapped to Theoretical Domain Framework domains, intervention functions, Behaviour Change Taxonomies and intervention components.

Barriers (B)/ Enablers (E)	TDF	BCW intervention function	BCT	SUPPORT TIA Intervention component
HCPs' lack of knowledge of potential needs post-TIA/ minor stroke (B).<br><br>HCPs' perceived role in follow-up care influenced their approach to identifying and addressing needs (B/E).	Knowledge<br><br>Social professional role and identity<br><br>Goal	Education/ Training	*Information about health consequences*<br><br>*Information about social and environmental consequences*<br><br>*Information about emotional consequences*<br><br>*Instruction on how to perform the behaviour*<br><br>*Identity associated with changed behaviour*	Training for intervention providers

For peer review only

Lack of follow-up pathway following rapid hospital specialist review (B).<br><br>Time constraints (B).<br><br>Nurses and AHPs were more holistic in their approach to care (compared to doctors) and considered this part of their role (E).	Environmental context and resources<br><br>Social professional role and identity<br><br>Intentions	Environmental restructuring	*Restructure physical environment*	Structured nurse/AHP-led follow-up appointment
Checklists considered useful prompts to identify needs (E).		Environmental restructuring/ Enablement	*Prompt/ cues*	Needs checklist completed by participants prior to the appointment
HCPs' lack of knowledge of support services and resources to support needs (B).<br><br>Directories of support services facilitated identification of support services (if up-to-date) (E).	Knowledge<br><br>Environmental context and resources	Environmental restructuring/ Enablement	*Instruction on how to perform the behaviour*<br><br>*Adding objects to the environment*	Resources to support management of needs, including a website of resources and support services; list of local support services; and a self-management booklet
Patients feel unsupported after hospital (B).<br><br>Difficult for patients to process and retain information (B).<br><br>Patients attempted to access information and support themselves, but found it overwhelming, confusing, contradictory (B).	Social influences	Environmental restructuring/ Enablement	*Action planning*<br><br>*Goal setting*<br><br>*Problem solving*<br><br>*Pharmacological support*	Action plan
Restricted communication between primary and secondary care (B).<br><br>Variability in the speed and content of GP letters (B/E)	Environmental context and resources<br><br>Social influences	Environmental restructuring/ Enablement	*Action planning*<br><br>*Prompts/cues*	Structured GP letter

For peer review only

TNO | | | |

My **information** and **support needs** after mini-stroke

This is a list of needs that some people have after mini stroke.

Please tick (✓) any areas you have difficulty with, even if you have only experienced it occasionally.

Your follow-up appointment is an opportunity to discuss any worries or problems you might be experiencing, and any advice or information you may need following your mini stroke (TIA) or minor stroke. Please use this list to help you to prepare for your appointment.

Information

1. I need more information about:	*Tick here*
• My diagnosis	
• My risk of stroke	
• Driving after my mini stroke	

Stroke prevention

2. I need advice on:	*Tick here*
• Medications for preventing another stroke	
• Medication side effects	
• Lifestyle change, such as exercise or diet, to prevent another stroke	

Effects of mini-stroke

3. Fatigue	*Tick here*
• I feel tired most of the time or I get easily tired	
• I find it difficult to concentrate and do things	
**4. Mood**	
• I feel anxious	
• I feel depressed	
• I experience anger, frustration or mood swings	
• I feel that my personality has changed	
**5. Memory and thinking**	
• I find it difficult to think, concentrate, or remember things	

For peer review only - http://bmjopen.bmj.com/site/about/guidelines.xhtml
For peer review only

TNO

6. Communication	
• I finding it difficult to understand / communicate with others	
• I have problems with speech, word finding or talking to others	
**7. Physical**	
• I experience muscle weakness or problems with balance	
• I have headaches	
• I am sensitive to noise or light	
**8. Incontinence**	
• I am having a problem controlling my bladder or bowels	
**9. Intimate relationships**	
• Since my mini stroke I have problems with sex	
**10. Work or education**	
• I am having problems at work or education	
• I would like support and advice on returning to work or education	
**11. Relationships with family or friends**	
• My personal relationships with my family or friends have become difficult or stressed	
**12. Social activities or daily tasks**	
• I find it difficult to take part in hobbies or leisure activities	
• I have difficulty doing daily tasks	

Which of the needs you identified above is your biggest concern?

Is there anything else you are concerned about or would like more information about?

Please bring this list to your follow-up appointment

For peer review only - http://bmjopen.bmj.com/site/about/guidelines.xhtml

eTable 2: Summary of proposed Patient Reported Outcome Measures (PROMs) for the main Randomised Controlled Trial.

Domain	PROM	Summary	Rationale
Health related quality of life	PROMIS-Global Health 10	**Questions:** 10<br>**Scoring:** 5-point Likert + 10 point Likert pain question. Scored using item-level calibrations- use the Health Measures Scoring Service<br>**MID:** none specified<br>**Permissions:** publicly available for use	Recommended by the International Consortium for Health Outcomes Measurement (2016) as part of a consensus stroke measure standard set
Health related quality of life	EQ-5D-5L	**Questions:** 5 + 1 VAS (20 cm)<br>**Scoring:** 5-point Likert scale and VAS. Score = 0-1<br>**MID:** for stroke: 0.08 to 0.12<br>**Permissions:** licence agreement needed (€600)	Used to calculate Quality Adjusted Life Years for health economics analyses
Anxiety/ depression	HADS	**Questions:** 14<br>**Scoring:** 4-point Likert Scale. 0-7 = Normal; 8-10 = Borderline abnormal; 11-21 = Abnormal<br>**MID:** none specified<br>**Permissions:** license agreement needed (0-1000 participants= £0.85 each)	Widely used in TIA/ minor stroke research, comprehensive psychometric evaluation
Fatigue	FAS	**Questions:** 10<br>**Scoring:** 5-point Likert Scale; Score = 10 (lowest fatigue) to 50 (highest fatigue). 10-21: no fatigue (normal), 22-50: substantial fatigue<br>**MID:** at least 4 points or 10% change of the baseline value<br>**Permissions:** must acknowledge the ild care foundation in the manuscript, FAS needs to be added as a keyword in the final publication, and PDF of the final publication must be send to the ild care foundation: info@ildcare.nl	Systematic review of fatigue scales for stroke scales identified the FAS had the best test-retest reliability
Self-efficacy	PAM-13	**Questions**: 13<br>**Scoring:** 5-point Likert Scale; Score 1-100: 4 Levels: Level 1 ≤47.0 (not believing activation is important), Level 2 47.1–55.1 (a lack of knowledge and confidence to take action), Level 3 55.2–67.0 (beginning to take action), level 4 ≥67.1 (taking action)<br>**MID:** not specified<br>**Permissions:** licence agreement NOT needed for feasibility studies	Used by NHS England
Medication adherence	MARS-5	**Questions:** 6<br>**Scoring:** 5-point Likert scale, score= 5 to 25 (higher scores= higher self-reported adherence)<br>**MID:** not specified<br>**Permissions:** Free access (subject to adequate citation) for Academic users	Validated in stroke patients, widely used
Satisfaction with care	Satisfaction with care question	Not applicable- non-validated bespoke question	

EQ-5D-5L: 5-level EuroQol 5-Dimensions; FAS: Fatigue Assessment Scale; HADS: Hospital Anxiety and Depression Scale; MARS-5: Medication adherence: Medication Adherence Rating Scale -5; MID: Minimally Important Difference; NHS: National Health Service; PAM-13: Patient Activation Measure-13; PDF: Portable Document Format; TIA: Transient Ischaemic Attack; VAS: Visual Analogue Scale

For peer review only

Protocol: additional information

Trial Steering Committee, Data Monitoring Committee and Study Oversight Committee

There will be no Trial Steering Committee or Data Monitoring Committee, the Study Oversight Committee will take the role of a joint Trial Steering Committee and Data Monitoring Committee.

The role of the independent Study Oversight Committee is to provide overall oversight of the trial, including the practical aspects of the study, as well as ensuring that the study is run in a way which is both safe for the patients and provides appropriate feasibility data to the sponsor and investigators.

The Study Oversight Committee will receive only aggregate data and will be blinded to the treatment allocation until the final analysis is presented. Members are independent of the investigators, their employing organisations, funders and sponsors. The Study Oversight Committee will report directly to the Trial Management Group.

Adverse events

The collection and reporting of Adverse Events will be in accordance with the UK Policy Framework for Health and Social Care Research and the requirements of the Health Research Authority. Definitions of different types of Adverse Events are listed in the table of abbreviations and definitions.

No risks are expected to arise from taking part in the trial. There are no Investigational Medicinal Products being used as part of the SUPPORT TIA trial. The intervention is considered low risk and consists of a nurse/AHP-led follow-up appointment which has been used in other populations and settings without evidence of harm. No Serious Adverse Events are anticipated as a unique consequence of participation in the SUPPORT TIA trial, but reporting requirements are clearly outlined in this section.

Adverse Events: There may be certain Adverse Events which are commonly expected in participants who have suffered a TIA or minor stroke. However, as these events are well characterised, it is highly unlikely that this trial will reveal any new safety information relating to this intervention. Therefore, we will not be collecting non-serious Adverse Events for this trial.

Serious Adverse Advents: Investigators should only report Serious Adverse Events which are attributable to the trial intervention. The above events are not considered related to the trial intervention and are therefore excluded from notification to the SUPPORT TIA Trial Office as Serious Adverse Events. These events should continue to be recorded in the medical records according to local practice. We are only reporting Serious Adverse Events which are attributable to the trial intervention; therefore, the control group will not be monitored.

Participant Withdrawal

Participants will be made aware at the beginning that they can freely withdraw (discontinue participation) from the trial (or part of) at any time. Types of withdrawal as defined are:

- The participant would like to withdraw from trial treatment, but is willing to be followed up in accordance with the schedule of assessments and, if applicable, using any central UK NHS bodies for long-term outcomes (i.e. the participant has agreed that data can be collected and used in the trial analysis).

For peer review only - http://bmjopen.bmj.com/site/about/guidelines.xhtml

For peer review only

- The participant would like to withdraw from trial treatment and does not wish to attend trial visits in accordance with the schedule of assessments but is willing to be followed up at standard clinic visits and, if applicable, using any central UK NHS bodies for long-term outcomes (i.e. the participant has agreed that data can be collected at standard clinic visits and used in the trial analysis, including data collected as part of long-term outcomes).
- The participant would like to withdraw from trial treatment and is not willing to be followed up in any way for the purposes of the trial and for no further data to be collected (i.e. only data collected prior to the withdrawal can be used in the trial analysis).

The details of withdrawal (date, reason and type of withdrawal) will be clearly documented in the study discontinuation form.

Note: participants involved in the qualitative sub-study may only withdraw from this part of the study up to the point of data analysis (five working days following the interview). After this point, it will not be possible to extract an individuals' interview data from the analyses.

Monitoring

Monitoring will be conducted as required following a risk assessment by the trials unit. Given the low-risk nature of this trial, central monitoring will be routine and no onsite monitoring is planned.

Onsite Monitoring: For this trial, no onsite monitoring is planned due to the low risk of the intervention and nature of the outcome data.

Central Monitoring: Trials staff will be in regular contact with the site research team to check on progress and address any queries that they may have. Recruitment rates, per site, will be monitored on a monthly basis. Trials staff will check incoming Informed Consent Forms and Case Report Forms for compliance with the protocol, data consistency, missing data and timing. Sites will be sent Data Clarification Forms requesting missing data or clarification of inconsistencies or discrepancies. Sites will be requested to send in copies of signed Informed Consent Forms and other documentation for in-house review for all participants providing explicit consent. Structured observations will be conducted as part of the trial of recruitment and consent procedures; intervention appointments; and end of study clinic appointments. Reports from these observations will be used to monitor protocol compliance.

Audit and Inspection: The Investigator will permit trial-related monitoring, audits, ethical review, and regulatory inspection(s) at their site, providing direct access to source data/documents. The investigator will comply with these visits and any required follow up. Sites are also requested to notify Birmingham Clinical Trials Unit (BCTU) of any relevant inspections.

Protocol amendments

If the Chief Investigator wishes to make a substantial amendment to the Research Ethics Committee (REC) application or the supporting documents, the Chief Investigator will submit a valid notice of amendment to the REC for consideration. It is the Sponsor's responsibility to decide whether an amendment is substantial or non-substantial for the purposes of submission to the REC.

Amendments will be notified to the REC and Health Research Authority (HRA), and communicated to the participating organisations (R&D office and local research team) departments of participating sites to assess whether the amendment affects the NHS permission for that site. The amendment history will be tracked to identify the most recent protocol version.

For peer review only - http://bmjopen.bmj.com/site/about/guidelines.xhtml

Confidentiality

Personal data recorded on all documents will be regarded as strictly confidential and will be handled and stored in accordance with the Data Protection Act 2018.

Participants will be identified using their unique trial identification number in correspondence between the site and BCTU. Participants will give their explicit consent for the movement of their consent form, giving permission for BCTU to be sent a copy. This will be used to perform in-house monitoring of the consent process. Participants will provide their personal contact details to the central research team at BCTU so they are able to contact the participants for follow-up questionnaires.

The Investigator must maintain documents not for submission to BCTU in strict confidence. In the case of specific issues and/or queries from the regulatory authorities, it will be necessary to have access to the complete trial records, provided that participant confidentiality is protected.

BCTU will maintain the confidentiality of all participant's data and will not disclose information by which participants may be identified to any third party, with the exception of the transcription service. A professional transcription company that already works with the University of Birmingham will transcribe the audio files. This company will be required to sign a confidentiality agreement before any files are sent to them. A member of the research team will check the transcripts once received from the transcription company and remove any names/ identifiers from the documents. Once the accuracy of the transcriptions has been confirmed, the original recordings will be deleted.

Representatives of the SUPPORT TIA trial team and sponsor may be required to have access to participant's notes for quality assurance purposes but participants should be reassured that their confidentiality will be respected at all times.

For participants involved in the qualitative aspects of the study, we will ask their permission to audio record the study interview using a digital recording device. We will then ask a reputable company to produce a written version of the recording called a transcript. The transcript company will need to sign a confidentiality agreement before they do so. We will then anonymise the transcript, removing all identifying information. After this, we will delete the original recording. We will only use anonymised quotes from the transcript in any arising publications or reports.

The research team will hold personal contact data for participants wishing to receive a summary of the results of the study - we anticipate this will be made available within 12 months of completion of the study. We will delete participants' contact details when the data is archived, meaning no personal identifiable data, other than study consent forms, will be retained.

There is potential that participants may disclose information that either indicates a risk or harm to themselves or others, evidence of malpractice or criminality. Participants will be informed in the Participant Information Sheet that if they disclose any of these issues, this will be reported to appropriate authorities.

Dissemination: Authorship eligibility guidelines and any intended use of professional writers

Publication policy: Results of this trial will be submitted for publication in a peer reviewed journal. The manuscript will be prepared by the Chief Investigator and authorship will be determined by the trial publication policy. No professional writers will be used.

For peer review only - http://bmjopen.bmj.com/site/about/guidelines.xhtml

Informed consent materials: Model consent form

I have read and understood the participant information sheet (Version 3.0, Dated 06/09/2021).	
I have had the opportunity to take time to consider my involvement in the trial and I have had the chance to ask questions, all of which have been answered to my satisfaction.	
I understand that my involvement in the trial is voluntary, and I am free to withdraw at any time without the quality of my medical care or my legal rights being affected.	
I understand that if I decide to withdraw from the trial, any information that has already been collected and anonymised may be used for analysis and publication.	
I understand a copy of this consent form and my data collected during the trial will be transferred to the central trials office at the Birmingham Clinical Trials Unit (BCTU), part of the University of Birmingham.	
I understand that all information collected will be used for medical research only and that I will not be identified in any way in the analysis and reporting of results. The personal information will include name, gender, date of birth, contact details and NHS number as well as medical information and study outcome assessments. It will be held securely and confidentially at BCTU.<br>I give permission for the transfer and storage of this data.	
I understand that relevant sections of my medical notes, information related directly to my participation in this trial, and data collected during the trial may be looked at by individuals from the University BCTU trial team, representatives of the sponsor, regulatory authorities, and the NHS Trust/Health Board where it is relevant to my taking part in this research.<br>I give permission for these individuals to have access to my records.	
I understand that my data collected during the trial may be shared with academic collaborative third parties who will help with the study's analysis. Before sharing, my personal identifiers (e.g. name, date of birth, contact details) will be removed and replaced with a trial number.	
I understand that my name and contact details will be used by the study team to contact me regarding the study. This may include, but not limited to, request additional information such as missing data on questionnaires I have completed.	
I understand that the research team have a duty to inform appropriate authorities if I disclose information that either indicates a risk or harm to myself or others, evidence of malpractice or criminality. In this circumstance confidentiality may be broken.	
I give permission to my GP being informed about my participation in the SUPPORT TIA Trial	
I voluntarily agree to take part in this study.	
Optional: I agree to be contacted to be invited for an interview about my involvement in the study and agree to my contact details being passed on to the research team at the University of Birmingham for them to contact me about this interview	
Optional: I would like to be sent a summary of the findings from the study and consent to my contact details to be held until this summary has been sent.	

Name of Participant	Signature	Date (DD/MMM/YYYY)
Name of Person taking Consent	Signature	Date (DD/MMM/YYYY)

For peer review only - http://bmjopen.bmj.com/site/about/guidelines.xhtml

For peer review only

For peer review only - http://bmjopen.bmj.com/site/about/guidelines.xhtml

STANDARD PROTOCOL ITEMS: RECOMMENDATIONS FOR INTERVENTIONAL TRIALS

SPIRIT 2013 Checklist: Recommended items to address in a clinical trial protocol and related documents*

Section/item	Item No	Description	Page
**Administrative information**			
Title	1	Descriptive title identifying the study design, population, interventions, and, if applicable, trial acronym	1
Trial registration	2a	Trial identifier and registry name. If not yet registered, name of intended registry	3
	2b	All items from the World Health Organization Trial Registration Data Set	n/a
Protocol version	3	Date and version identifier	n/a
Funding	4	Sources and types of financial, material, and other support	15
Roles and responsibilities	5a	Names, affiliations, and roles of protocol contributors	1
	5b	Name and contact information for the trial sponsor	1
	5c	Role of study sponsor and funders, if any, in study design; collection, management, analysis, and interpretation of data; writing of the report; and the decision to submit the report for publication, including whether they will have ultimate authority over any of these activities	15
	5d	Composition, roles, and responsibilities of the coordinating centre, steering committee, endpoint adjudication committee, data management team, and other individuals or groups overseeing the trial, if applicable (see Item 21a for data monitoring committee)	~~REC protocol~~ Appendix
**Introduction**			
Background and rationale	6a	Description of research question and justification for undertaking the trial, including summary of relevant studies (published and unpublished) examining benefits and harms for each intervention	5
	6b	Explanation for choice of comparators	9
Objectives	7	Specific objectives or hypotheses	5

For peer review only - http://bmjopen.bmj.com/site/about/guidelines.xhtml

Trial design	8	Description of trial design including type of trial (eg, parallel group, crossover, factorial, single group), allocation ratio, and framework (eg, superiority, equivalence, noninferiority, exploratory)	5
**Methods: Participants, interventions, and outcomes**			
Study setting	9	Description of study settings (eg, community clinic, academic hospital) and list of countries where data will be collected. Reference to where list of study sites can be obtained	6
Eligibility criteria	10	Inclusion and exclusion criteria for participants. If applicable, eligibility criteria for study centres and individuals who will perform the interventions (eg, surgeons, psychotherapists)	6
Interventions	11a	Interventions for each group with sufficient detail to allow replication, including how and when they will be administered	7-9
	11b	Criteria for discontinuing or modifying allocated interventions for a given trial participant (eg, drug dose change in response to harms, participant request, or improving/worsening disease)	n/a
	11c	Strategies to improve adherence to intervention protocols, and any procedures for monitoring adherence (eg, drug tablet return, laboratory tests)	n/a
	11d	Relevant concomitant care and interventions that are permitted or prohibited during the trial	n/a
Outcomes	12	Primary, secondary, and other outcomes, including the specific measurement variable (eg, systolic blood pressure), analysis metric (eg, change from baseline, final value, time to event), method of aggregation (eg, median, proportion), and time point for each outcome. Explanation of the clinical relevance of chosen efficacy and harm outcomes is strongly recommended	10-12
Participant timeline	13	Time schedule of enrolment, interventions (including any run-ins and washouts), assessments, and visits for participants. A schematic diagram is highly recommended (see Figure)	5 (figure 1)
Sample size	14	Estimated number of participants needed to achieve study objectives and how it was determined, including clinical and statistical assumptions supporting any sample size calculations	6
Recruitment	15	Strategies for achieving adequate participant enrolment to reach target sample size	6
**Methods: Assignment of interventions (for controlled trials)**			
Allocation:			7

For peer review only - http://bmjopen.bmj.com/site/about/guidelines.xhtml

Sequence generation	16a	Method of generating the allocation sequence (eg, computer-generated random numbers), and list of any factors for stratification. To reduce predictability of a random sequence, details of any planned restriction (eg, blocking) should be provided in a separate document that is unavailable to those who enrol participants or assign interventions	7
Allocation concealment mechanism	16b	Mechanism of implementing the allocation sequence (eg, central telephone; sequentially numbered, opaque, sealed envelopes), describing any steps to conceal the sequence until interventions are assigned	7
Implementation	16c	Who will generate the allocation sequence, who will enrol participants, and who will assign participants to interventions	7
Blinding (masking)	17a	Who will be blinded after assignment to interventions (eg, trial participants, care providers, outcome assessors, data analysts), and how	7
	17b	If blinded, circumstances under which unblinding is permissible, and procedure for revealing a participant's allocated intervention during the trial	7
**Methods: Data collection, management, and analysis**			
Data collection methods	18a	Plans for assessment and collection of outcome, baseline, and other trial data, including any related processes to promote data quality (eg, duplicate measurements, training of assessors) and a description of study instruments (eg, questionnaires, laboratory tests) along with their reliability and validity, if known. Reference to where data collection forms can be found, if not in the protocol	10-12
	18b	Plans to promote participant retention and complete follow-up, including list of any outcome data to be collected for participants who discontinue or deviate from intervention protocols	n/a
Data management	19	Plans for data entry, coding, security, and storage, including any related processes to promote data quality (eg, double data entry; range checks for data values). Reference to where details of data management procedures can be found, if not in the protocol	
Statistical methods	20a	Statistical methods for analysing primary and secondary outcomes. Reference to where other details of the statistical analysis plan can be found, if not in the protocol	13
	20b	Methods for any additional analyses (eg, subgroup and adjusted analyses)	n/a

For peer review only - http://bmjopen.bmj.com/site/about/guidelines.xhtml

	20c	Definition of analysis population relating to protocol non-adherence (eg, as randomised analysis), and any statistical methods to handle missing data (eg, multiple imputation)	n/a
**Methods: Monitoring**			
Data monitoring	21a	Composition of data monitoring committee (DMC); summary of its role and reporting structure; statement of whether it is independent from the sponsor and competing interests; and reference to where further details about its charter can be found, if not in the protocol. Alternatively, an explanation of why a DMC is not needed	Appendix ~~REC protocol~~
	21b	Description of any interim analyses and stopping guidelines, including who will have access to these interim results and make the final decision to terminate the trial	n/a
Harms	22	Plans for collecting, assessing, reporting, and managing solicited and spontaneously reported adverse events and other unintended effects of trial interventions or trial conduct	Appendix ~~REC protocol~~
Auditing	23	Frequency and procedures for auditing trial conduct, if any, and whether the process will be independent from investigators and the sponsor	Appendix ~~REC protocol~~
**Ethics and dissemination**			
Research ethics approval	24	Plans for seeking research ethics committee/institutional review board (REC/IRB) approval	15
Protocol amendments	25	Plans for communicating important protocol modifications (eg, changes to eligibility criteria, outcomes, analyses) to relevant parties (eg, investigators, REC/IRBs, trial participants, trial registries, journals, regulators)	Appendix ~~REC protocol~~
Consent or assent	26a	Who will obtain informed consent or assent from potential trial participants or authorised surrogates, and how (see Item 32)	6
	26b	Additional consent provisions for collection and use of participant data and biological specimens in ancillary studies, if applicable	n/a
Confidentiality	27	How personal information about potential and enrolled participants will be collected, shared, and maintained in order to protect confidentiality before, during, and after the trial	Appendix ~~REC protocol~~
Declaration of interests	28	Financial and other competing interests for principal investigators for the overall trial and each study site	15
Access to data	29	Statement of who will have access to the final trial dataset, and disclosure of contractual agreements that limit such access for investigators	13

review only

For peer review only - http://bmjopen.bmj.com/site/about/guidelines.xhtml

Ancillary and post-trial care	30	Provisions, if any, for ancillary and post-trial care, and for compensation to those who suffer harm from trial participation	n/a
Dissemination policy	31a	Plans for investigators and sponsor to communicate trial results to participants, healthcare professionals, the public, and other relevant groups (eg, via publication, reporting in results databases, or other data sharing arrangements), including any publication restrictions	15
	31b	Authorship eligibility guidelines and any intended use of professional writers	Appendix ~~REC protocol~~
	31c	Plans, if any, for granting public access to the full protocol, participant-level dataset, and statistical code	n/a
**Appendices**			
Informed consent materials	32	Model consent form and other related documentation given to participants and authorised surrogates	Appendix ~~REC protocol~~
Biological specimens	33	Plans for collection, laboratory evaluation, and storage of biological specimens for genetic or molecular analysis in the current trial and for future use in ancillary studies, if applicable	n/a

*It is strongly recommended that this checklist be read in conjunction with the SPIRIT 2013 Explanation & Elaboration for important clarification on the items. Amendments to the protocol should be tracked and dated. The SPIRIT checklist is copyrighted by the SPIRIT Group under the Creative Commons "Attribution-NonCommercial-NoDerivs 3.0 Unported" license.

For peer review only
